# Sowing seeds for the future: Future time perspective and climate adaptation among farmers

**DOI:** 10.1111/bjso.12850

**Published:** 2025-02-06

**Authors:** C. Dale Shaffer‐Morrison, Naseem H. Dillman‐Hasso, Robyn S. Wilson

**Affiliations:** ^1^ Department of Psychology University of Essex Essex UK; ^2^ School of Environment and Natural Resources The Ohio State University Columbus Ohio USA

**Keywords:** climate adaptation, conservationist norms, future time perspectives

## Abstract

A future time perspective is critical to domains where outcomes of choices are delayed and potentially catastrophic: such as with agriculture where management decisions today are critical to the viability of multiple outcomes in the future. Farmers are on the front lines of climate change where shifts in rainfall and temperature threaten the viability of crop production. This reality is compounded for some farmers who lack the resources needed to adapt. Prior work has shown that farmers with strong injunctive norms towards conservation, and sufficient resources, are more likely to implement adaptation practices, but little research has explored the role of future time perspectives relative to these factors. We test whether future time perspective may lead US Midwestern farmers to develop injunctive norms towards conservation, and in turn, implement adaptation practices. We find support for this mechanism through both a correlational analysis (Study 1), and a manipulation of the salience of future impacts through a vignette experiment (Study 2). In addition, we see that some socioeconomic resources constrain adaptation. These results are relevant to regions where greater adaptation practices are needed to protect against climate impacts on operations that produce row crops like corn, soy and wheat.

## INTRODUCTION

It is well documented that agriculture is a contributor to climate change (Gil et al., 2019; Khatri‐Chhetri et al., 2022; Liao et al., 2023), and that farmers themselves are vulnerable. For example, shifts in rainfall, temperature and extreme weather associated with climate change impact crop yield (Owino et al., 2022). Farmers with lower socioeconomic resources are at greater risk given limited access to the resources needed to recover from the effects of climate‐exacerbated weather events (Below et al., 2012; Ojumu et al., 2020; Roesch‐McNally et al., 2018). A lack of resources can further strain a farmer's ability to invest in adaptive practices that increase resilience in the short‐ and long‐term (Berry et al., 2011). These adaptive practices are wide ranging, but include those already promoted through conservation agriculture programmes to increase soil health and promote greater diversity in the crop system (i.e. limited tillage, continuous living cover and diverse crop rotations) (Stagnari et al., 2010; Wezel et al., 2014).

Engaging in adaptive practices benefits not only the local community and environment (Deines et al., 2019; McLellan et al., 2018; Palm et al., 2014), but also lessens the impact of climate‐induced stress on crops (and by extension the farmer) over the long‐term. For example, reduced tillage allows for more flexible planting dates and soil moisture retention in the event of changing weather patterns (e.g. improved water holding capacity in the soil, reducing erosion and nutrient runoff), while crop diversity and reduced tillage together improve yield stability (Gaudin et al., 2015). Such practices are commonly supported by existing government programmes around the globe including in the United States (Knowler & Bradshaw, 2007), Canada (Agriculture and Agri‐Food Canada, 2022) and Brazil (Inter‐American Institute for Cooperation on Agriculture, 2021), and have been named by the United Nations as Sustainable Development goals (UN General Assembly, 2015). However, enrollment in voluntary programmes and associated adoption of these practices remains low in some developed contexts (Arbuckle, 2013; Kast et al., 2021; Ribaudo, 2015), as does concern about climate‐exacerbated impacts to the operation (Mase et al., 2017). Evidence that farmers are motivated to engage in conservation agriculture (Gbetibouo et al., 2010; Morris & Arbuckle, 2021; Sumaryanto Susilowati et al., 2022) begs the question as to why such programmes are not more popular, and how more farmers can be effectively engaged. Recent work has highlighted the need to focus research on high‐impact climate mitigation behaviours (Nielsen et al., 2021), but these calls often overlook industry sectors with high environmental impact, such as agriculture, which are also in need of adaptation. Due to the impact of adaptation behaviours on crop stability in the face of climate change, more work is needed to understand psychological factors that can contribute to adaptation among farmers (Wilson et al., 2020). Further, due to the future‐oriented nature of thinking about the effects of climate on agricultural production, and planning for how to adapt to it, the present studies aimed to test how a farmer's future time perspective can impact their climate adaptation strategies.

Two potential explanations are typically given for the relatively low engagement in such practices: a lack of the necessary socioeconomic resources, and a lack of motivation. Specifically, existing work indicates that farmers with greater resources (e.g. larger farms, more education) are more likely to engage in conservation agriculture (Farr et al., 2018; Gbetibouo et al., 2010; Lu et al., 2022). Other research shows that conservation agriculture is more common among farmers who believe that a ‘good farmer’ is concerned about soil health and water quality (Gao & Arbuckle, 2022; Lu et al., 2022; McGuire et al., 2015; Prokopy et al., 2019; Schoolman & Arbuckle, 2022), representing a potential motivational mechanism that is based in injunctive norms towards conservation (hereafter, conservationist norms). Future time perspective, or the tendency to orient oneself towards the future, remains a potentially understudied explanation (Zimbardo & Boyd, 1999). Individuals who tend to think more about the future tend to place more value on the future benefits generated by engaging in action today. Prior research indicates that farmers with a future time perspective tend to focus more on the impact of climate change on future operations (Morris & Arbuckle, 2021; Schattman et al., 2021), and as a result may be more motivated to engage in climate adaptation practices. The research reported here explores how engagement in climate adaptation in agriculture is impacted by: (1) future time perspective (both in general and through increasing the salience of the future consequences of climate change), (2) how future time perspective may increase conservationist norms and (3) the extent to which these effects hold for farmers with limited socioeconomic resources. These ideas were tested using a survey that measured future time perspectives (Study 1), as well as a vignette experiment that increased the salience of future climate impacts (Study 2), all while controlling for socioeconomic resources. To begin, we review the literature on future time perspectives, conservationist norms and socioeconomic resources and the potential role of such effects on adaptation behaviour.

It is challenging to promote behaviours that do not provide immediate benefits for the adopter (Du et al., 2002; Odum et al., 2020). However, prior research indicates that whether an individual is willing to take actions with future benefits is a function of how they think about and value the future (Göllner et al., 2018; Stolarski et al., 2011; Ye et al., 2022). This construct has been studied across a variety of contexts and cultures around the world (Kooij et al., 2018; Sircova et al., 2014). The association between future time perspective and behaviour may be driven by a reduced concern about immediate costs, and an increased focus on future benefits (Arnocky et al., 2014; Olsen & Tuu, 2021). Further, tending to think more about the future is associated with greater planning (Zimbardo & Boyd, 1999), as well as monitoring progress towards a goal (Baird et al., 2021). This work has also indicated an association between future time perspectives and more conservation‐focused behaviours (Milfont et al., 2012; Olsen & Tuu, 2021; Segev & Liu, 2022), including agriculturally relevant behaviours such as water conservation (Corral‐Verdugo et al., 2006), and low emissions practices (Morgan et al., 2015). In the context of agricultural practices, many conservation‐focused behaviours are also relevant to adapting to the pressures of climate change. For example, reducing soil tillage and planting small grains and cover crops help maintain soil nutrients, reduce erosion and lessen fertilizer runoff that can cause harmful algae blooms and other environmental issues (Kassam et al., 2019). Each of these can also be considered climate adaptation, as prior work has shown climate change increases the risk of nutrient loss, erosion and runoff through changing temperature and rainfall patterns (Mirzabaev et al., 2023).

### Future time perspectives and conservation behaviour

Existing future time perspective and conservation behaviour work largely focus on small behaviours with little impact on the rest of one's day‐to‐day activities. For example, studies focus on recycling (Milfont et al., 2012) or opting to use public transit rather than a personal vehicle (Joireman et al., 2004). Enacting such behaviours rarely requires meticulous planning, leading some to argue that the tendency to plan does *not* explain why having future time perspectives helps a person reach their goals (Baird et al., 2021). Rather, it is the tendency to monitor progress towards a goal that explains the relationship between future time perspective and successfully reaching a goal. However, in the case of on‐farm climate adaptation, enacting a new practice does involve meticulous planning *and* monitoring of progress.

Implementing an adaptation practice has whole‐farm implications during an entire growing season and beyond. Because the tendency to plan and keep track of progress are features of having a future time perspective (Zimbardo & Boyd, 1999), the present study tested whether having a future time perspective alone is sufficient to directly predict engagement in adaptation. Among farmers, having a specific plan predicts some adaptation behaviours (Morris & Arbuckle, 2021), and thinking about future needs predicts adaptation practices (Shariatzadeh & Bijani, 2022). Cross‐sectional studies also demonstrate that farmers who perceive future risks from climate change are more likely to support adaptation (Arbuckle et al., 2015; Houser et al., 2022; Mase et al., 2017; Roesch‐McNally et al., 2017), as do those farmers who believe the negative effects of climate change are closer in time (Azadi et al., 2019). However, unlike the present study, neither of these prior studies examined a farmer's general tendency towards planning and thinking about the future.

### Conservationist norms and conservation behaviour

In addition to engaging in greater planning, a mechanism by which future time perspective might increase on‐farm adaptations is through injunctive norms, or how a person thinks they and others ought to behave. This mechanism is well documented in the broader literature on sustainable behaviours where norms interact with other factors to influence behaviour (Klöckner, 2013; Stern, 2002). This has been found in agricultural contexts as well; farmers who believe that a ‘good farmer’ cares about conservation tend to engage in more conservation practices (Gao & Arbuckle, 2022; Lu et al., 2022; Prokopy et al., 2019; Schoolman & Arbuckle, 2022). Further, farmers who feel personally responsible towards addressing climate change take more adaptive actions (Zhang et al., 2020).

In the present studies, we make the prediction that orienting oneself towards the future helps to develop conservationist norms among farmers, which then leads to more adaptation behaviour. Conservation‐focused norms have been shown to predict more environmentally‐oriented behaviour (Niemiec et al., 2020). According to value‐belief‐norm theory, personal norms towards taking action are activated by perceived threats to individual values and an ascription of responsibility, and these norms then motivate behaviour (e.g. Stern et al., 1999). The role of injunctive norms in predicting behaviour is also supported by work on individual actions (Cialdini et al., 1990), in work settings (Mouro & Duarte, 2021) and in agricultural production (Zhang et al., 2020). According to Joireman and Liu's (2014) mediation awareness model, those who are more concerned with future consequences, generally, are more likely to be aware of threats posed by climate change and come to value conservation in order to protect from those threats. In the case of agriculture, perceived threats of climate change on the environment and growing crops, and by extension one's livelihood, could activate norms towards personally enacting climate adaptation practices. However, missing from this behavioural puzzle is an understanding of how these norms develop.

As mentioned above, climate adaptation strategies in the agricultural context require, sometimes extensive, planning for how to implement complex changes in the farming operation, such as how crops receive adequate nutrients and water, and when crops are planted and harvested. In addition, perceiving the benefits of climate adaptation requires farmers to think beyond the present growing season, to protect the viability of crops against future climate change‐driven fluctuations in temperature and precipitation. We extend existing work by hypothesizing that a future time perspective would allow farmers to think more about, and place higher value on, the benefits of climate adaptation practices for maintaining successful crop growing—which are needed to protect both the environment and a farmer's livelihood—therefore, leading to higher conservationist norms. In turn, higher conservationist norms would predict more adaptation actions (Kim & Seock, 2019).

### Socioeconomic considerations

Given prior research (Liu et al., 2018; e.g. Lu et al., 2022), it was expected that farmers with lower socioeconomic resources would be less likely to adapt, as many adaptations require significant on‐farm resources. For example, individuals with larger farms, benefitting from higher income, are better equipped to instal certain structural practices, like water flow management systems that help protect against floods or drought. Although many voluntary incentive‐based programmes pay farmers for such practices, these programmes are reimbursement‐oriented. This can be especially limiting for smaller farms with less farm income, who have less capital to invest in up‐front costs (Piñeiro et al., 2020). Education can also play a role; where formal education leads to greater exposure to technical information about adaptation, which instils confidence (Perry & Davenport, 2020). In a Midwestern US context, farmers with greater formal education were more likely to have repeated interactions with agriculture extension programmes, leading to greater trust and changes in practice (Anang et al., 2020; Morris & Arbuckle, 2021; Ranjan et al., 2019). Greater formal education is also correlated with concern for climate change (Hornsey et al., 2016), and both education and socioeconomic resources generally predict having a greater future time perspective (Padawer et al., 2007). Finally, even livestock can be viewed as an asset, as they provide income diversity, which can protect against the effects of climate change on crop stress. Livestock may also increase the benefits of certain practices, such as a reduced‐tillage management system, where animals can graze on the organic matter left on the untilled surface of fields. As a result, it was hypothesized that greater SES (operationalized as farmers with larger farms, more education and the presence of livestock) would significantly predict adaptation above and beyond these socio‐psychological effects.

### Present research

**FIGURE 1:**
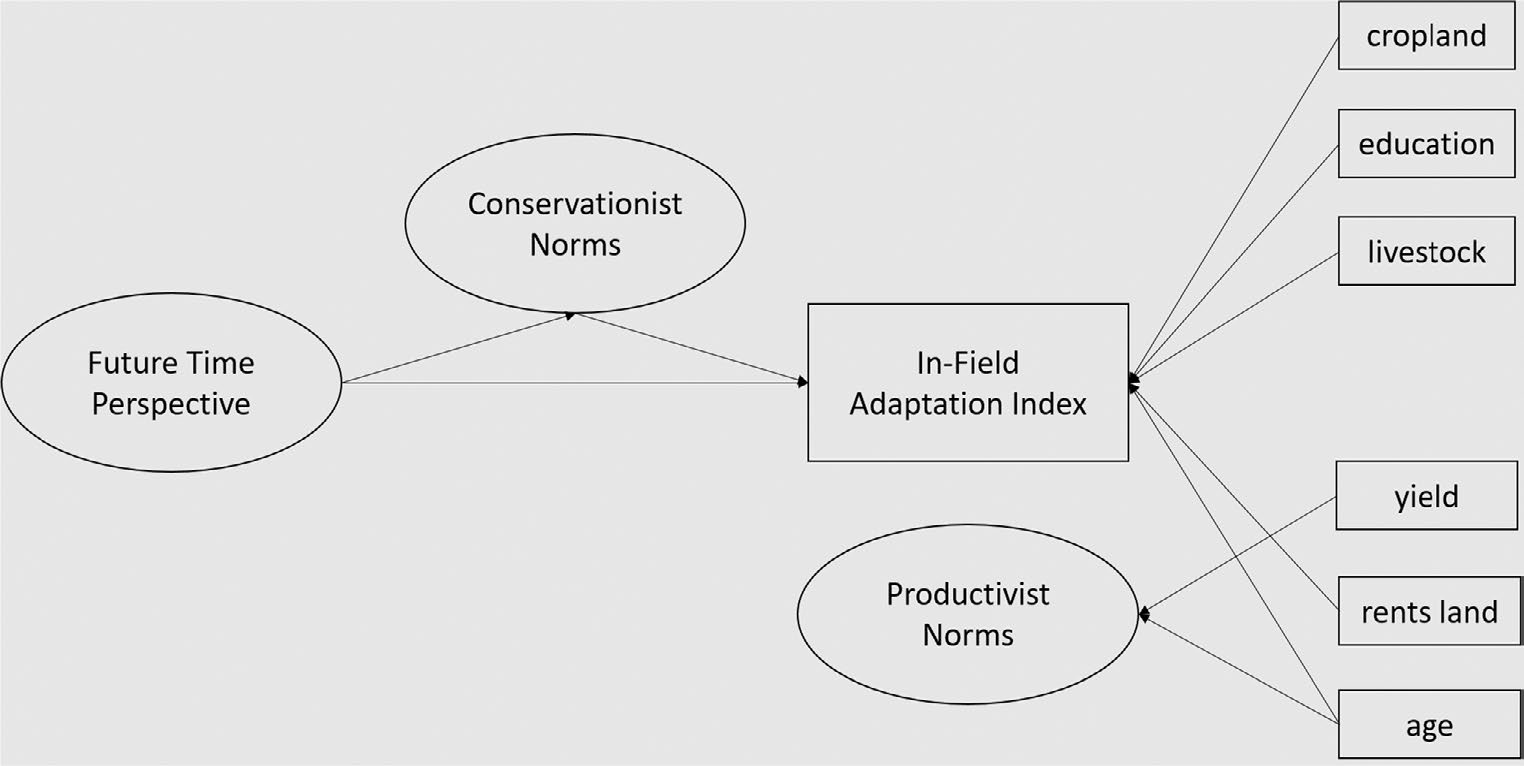
Study 1 Conceptual Model Predicting In‐Field Adaptations Index. The conceptual model pictured here reflects the hypothesis that conservationist norms, which predict engaging in actual in‐field adaptations, develop partly as a result of the tendency to think about and plan for the future.

The present studies were designed to assess the role of future time perspective and socioeconomics in explaining on‐farm climate adaptation, beyond the hypothesized effects of action‐relevant conservationist norms. Study 1 tested whether planning for the future (future time perspective) alone was sufficient to explain adaptation, or if a farmer's general tendency to think about the future explains conservationist norms—and in turn greater in‐field adaptation. This is one of the few known studies to quantitatively test predictors of the development of such norms among farmers. These effects are also examined while controlling for potential socioeconomic factors (see Figure 1).

Study 2 expands on the idea that thinking about the future can impact adaptation by experimentally testing how stated future changes in weather patterns impact adaptation plans, and whether baseline concern about future climate impacts and resource availability still affect adaptation plans in the face of changing conditions. One could reason that as changing weather patterns increase adaptation needs, all farmers will be similarly motivated to plan to adapt out of necessity, eclipsing any effect of resource availability or pre‐existing concerns about the impact of climate change on agriculture. Our vignette experiment was able to explicitly test these competing hypotheses. Limited research has used this paradigm to test the effects of thinking about future changes in weather patterns on adaptation. A few studies have shown farmers have greater intention to adapt in response to increased drought via irrigation management (Drugova et al., 2022; Krah et al., 2019; Wens et al., 2022), or adaptation intentions in response to climate change generally (Roesch‐Mcnally et al., 2017). However few, if any, studies have used vignette experiments (or choice experiments) to measure changes to in‐field adaptation plans.

Data, code, survey instruments and other supporting information for both studies can be found at https://osf.io/8hdpt/.

## STUDY 1

Building on existing literature, which finds that (1) planning for the future is an essential component of taking actions with future benefits and (2) those who think more about the future tend to take more conservation actions, we hypothesize:

1. Future time perspective will directly explain greater adaptation.
2. Future time perspective will directly explain higher conservationist norms.
3. Conservationist norms will mediate the relationship between future time perspective and adaptation.
4. Higher socioeconomic resources (e.g. more land, more education, more livestock) will directly explain greater adaptation.

### Materials and methods

#### Study procedure

To test these relationships, we collected data through a survey of farm operators in the Great Lakes region of the United States (i.e. the states of Ohio, Michigan, Indiana, Illinois and Wisconsin). This is a significant commodity crop region that also has great potential for carbon sequestration, a climate change mitigation strategy and other provisioning services like drinkable water, which is important for climate adaptation. As with many farming regions throughout the world, the Great Lakes region faces numerous challenges to growing crops due to climate change—warming temperatures, floods and precipitation changes (Melillo et al., 2014; Mirzabaev et al., 2023).

We stratified our sample by farm size and by state, to ensure a better representation of acres farmed (versus people farming) in the region. Prospective participants were contacted through a mailing sample obtained from Farm Market ID. Farmers were sent an invitation letter, followed by a paper‐and‐pencil survey 3 days after. A return envelope and a token incentive of a $2 bill were included with the survey. If no response was received, reminder postcards were also sent 2 and 3 weeks afterwards. The survey was administered in 2019 (see Data S1 for administration details).

A total of 1115 completed surveys were returned from 609 operators and 506 non‐operating landowners. Data from operators are presented in this study, as only operators were asked for the complete set of items used here. A full list of questions that were part of the survey, as well as exact wording, are available in the Data S1. After being presented with the informed consent information, operators first answered questions about their farming operation. Next, farmers answered the individual difference measures and demographic questions described below.

#### Measures

Descriptive statistics are shown in Table S1. The dependent variable, adaptation behaviour, was conceptualized using practices that are identified globally as conservation agriculture (Kassam et al., 2019): tillage (such as not tilling the soil or reduced tilling), use of cover crops and having small grains in rotation. Operators indicated the extent to which they had implemented each of these practices on their farm, and their responses were combined to form a weighted index of behaviour (Shaffer‐Morrison & Wilson, 2024). Detailed weighting procedure and code are available in the Data S1.

To measure future time perspective, we used a shortened version of the Future subscale of the Zimbardo Time Perspective Inventory (ZPTI) (Zimbardo & Boyd, 1999). Items were chosen based on the seven highest factor loadings from Zimbardo and Boyd (1999) and pertained to one's tendency to plan their days, pursue future goals and meet deadlines (*α* = .688). This measure was chosen because we reasoned that these themes capture elements needed to plan complex farming operations.

To measure conservationist and productivist injunctive norms we used the conservationist and productivist subscales of the Farmer Identity Scale (Arbuckle, 2013; McGuire et al., 2015). Although the present studies were primarily interested in conservationist norms, we controlled for productivist norms or the belief that a good farmer tends to maximize crop production and profit. The nine‐item conservationist subscale (*α* = .845) asks how important each feature of a farmer is, such as, ‘A good farmer is one who puts long‐term conservation of farm resources before short‐term profits’. The eight‐item productivist subscale (*α* = .827) includes items like, ‘A good farmer is one who has the highest yields per acre’.

We measured several socioeconomic resource variables, including formal education level, total cropland acres and presence of livestock (dichotomized), as they indicate greater farmer and on‐farm assets, and diversification of resources. Self‐reported formal education ranged from ‘some high school’ to ‘graduate or professional degree’. The self‐reported total number of cropland acres was log‐transformed to normalize its distribution. The presence of livestock was dichotomously indicated by participants. We also measured yield, tenure and age as it is possible that the motivation to engage in conservation would be lower for those with higher yields (Lu et al., 2022), who rent land (Ranjan et al., 2019), or who are older (Mase et al., 2017). For yield, farm operators reported typical corn and soy yields in bushels per acre in the previous growing season. To create an index of yield, these were combined by first z‐scoring each across the whole sample, then averaging both if applicable, thereby allowing for missing data if one crop had not been grown in the previous growing season. For tenure, farmers were asked whether they rent land from others, measured dichotomously. Participants also entered their age.

#### Statistical method

Structural equation models were estimated using the ‘sem’ command in Stata version 13.1 (StataCorp, 2013). First, we used confirmatory factor analysis to test the fit of future time perspective, conservationist norms and productivist norms. Separate structural equation models were fit for each scale, using single‐factor solutions. Details regarding model fit are available in the Data S1. Final models for future time perspective, conservationist norms and productivist norms all achieved acceptable levels of fit (Schreiber, 2008; West et al., 2012).

**FIGURE 2:**
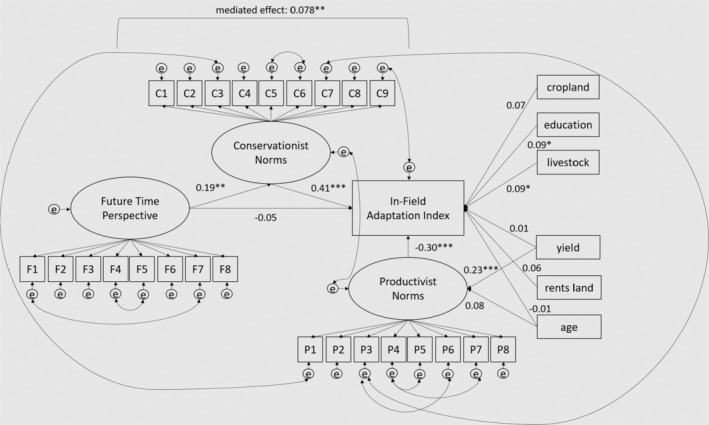
Structural equation model predicting in‐field adaptations index. The final model showed excellent fit, as measured by RMSEA and SRMR (West et al., 2012), whereas the CFI and TLI closely bordered acceptable fit (Schreiber et al., 2006) (see Table 1). Examination of the quantile plot of the adaptation behaviour index approximated a diagonal line. Thus, this model was deemed to be acceptable. The model predicted 73% of the overall variance in the data (*R* ^2^ = .734).

After fitting the measurement models, they were entered into the partially latent structural equation model predicting the index of in‐field adaptation (see Figure 2). All exogenous variables were allowed to covary, although arrows were excluded from Figure 2 to improve readability. Our dependent variable, the In‐Field Adaptation Index, was predicted by socioeconomic and control variables, future time perspective and conservationist and productivist norms. Because productivist norms tend to be higher among older farmers and those with greater yields (Conway et al., 2021; McGuire et al., 2013), these paths were also included in the model. All continuous exogenous predictors were *z*‐scored to standardize output. The initial model fit was not adequate but was greatly improved through the addition of theoretically sound error covariances. Details regarding fit and modifications for each model iteration are shown in the Data S1 and Table S3.

After fitting the final model predicting the index, we used the ‘medsem’ package (Mehmetoglu, 2018) to estimate the indirect effect of future time perspective on the index through conservationist norms. This package uses Zhao et al., (2010) mediation approach, estimating the size of the indirect effect using Monte Carlo simulation.

### Results

#### Descriptives

Farmers had a median farm size of 600 acres and average age of 60 years. The average formal education level among farmers was between ‘some college’ and ‘associate's degree’, and most (69%) did not own livestock, but most (68%) did rent land from others. Across the sample, agreement that one tends to orient towards the future was positively skewed, with most persons tending to agree that they have a future time perspective (*M* = 0.982, SD = 0.488, scale range − 2 to 2). Conservationist norms were also high in this sample (*M* = 3.112, SD = 0.520, scale range 0–4), whereas productivist norms were distributed around the scale mean (*M* = 2.333, SD = 0.715). Complete data for inclusion in the structural equation model was collected from 453 participants.^1^ A table of descriptive statistics is available in the Data S1.

**FIGURE 3:**
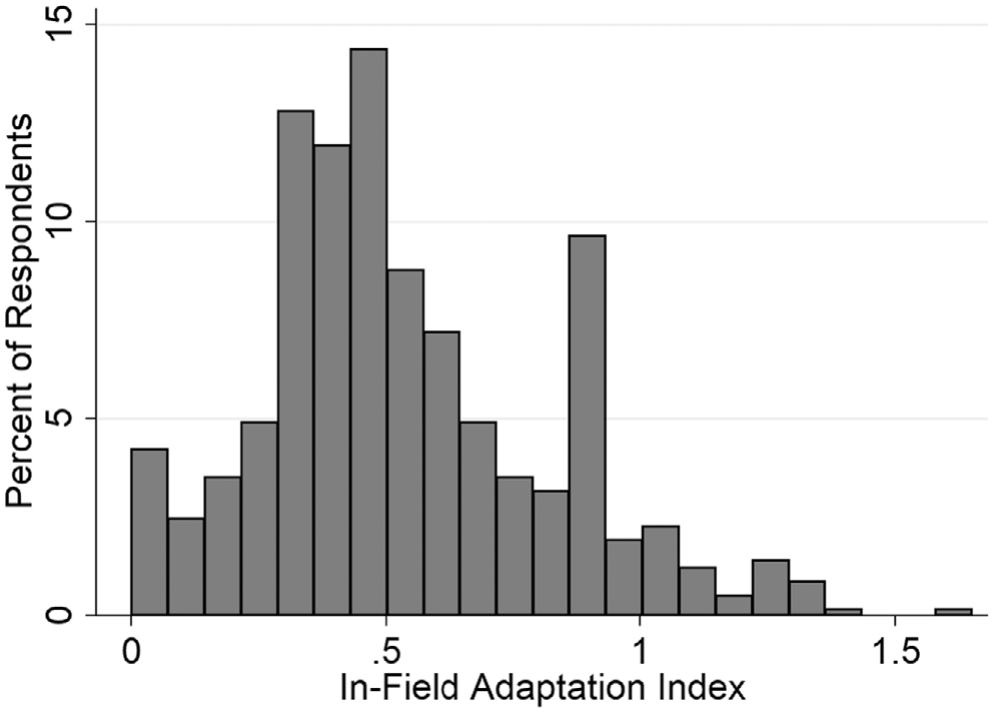
Histogram of distribution of in‐field adaptation index. For the index of behaviour, modal scores in the data correspond to the following practices: 0.33 = all acres in conservation tillage, but no other practices (*n* = 47, 8%); 0.47 = all acres in rotational no‐till, but no other practices (*n* = 24, 4%); 0.93 = all acres in no‐till, but no other practices (*n* = 37, 6%).

All but 12 farm operators in this sample had implemented at least some conservation behaviours (see Figure 3). Regarding tillage, 10% had implemented rotational no‐till on all acres, while 12% had implemented no‐till on all acres. Most of the sample had either no years of small grains (e.g. small grains) in rotation (58%), or 1 year (28%) of small grains in a five‐year rotation. Regarding cover crops, the majority had not planted any cover crops in the last growing season (68%), and only about 5% of the sample had planted cover crops on at least half of their cropland acres.

#### Structural equation model predicting in‐field adaptations index

The final model is shown in Figure 2, and fit statistics are shown in Table 1. Full model estimates (including factor loadings, variances and residual covariances) are included in Table S1.

**TABLE 1:** Fit of Final Model Predicting Behavioural Index.

	Chi‐square model versus saturated	RMSEA	CFI	TLI	SRMR	*R* ^2^	*n*
Final model	860.237, *p* < .001	0.047	0.883	0.868	0.058	0.734	453
Acceptable fit	(often failed with large samples)	<0.08	>0.90	>0.90	<0.10	‐	
Ideal fit	Ideally, n.s.	<0.06	>0.95	>0.95	<0.08	‐	

*Note*: Model fit statistics for the mediation model predicting the behavioural index. Acceptable and ideal fit metrics are taken from (Schreiber et al., 2006 Table 2) and (West et al., 2012).

The first hypothesis, that a future time perspective would have a direct relationship with adaptation behaviour, was not supported (*p* = .343). Thus, it appears that the components of future time perspective, which relate to the tendency to plan and think about future goals, are not sufficient to engender greater in‐field adaptation. However, consistent with Hypothesis 2, future time perspective was associated with higher conservationist norms (*p* = .001), which in turn was associated with greater adaptation (*p* < .001). In support of Hypothesis 3, this indirect effect of future time perspective on adaptation, through its effects on conservationist norms, was significant (*B* = 0.078, *p* = .003). This mediated effect was significant beyond other factors that are known to relate to adaptation (e.g. cropland yield, land tenure and farmer age). Hypothesis 4 was partially supported, as higher education and the presence of livestock were associated with greater adaptation, but a number of cropland acres had no association with adaptation.

### Study 1 discussion

The results of Study 1 are consistent with the hypothesis that orienting oneself towards the future may increase the injunctive norm, in the form of placing greater importance on conservation, which in turn begets greater adaptation. Further, farmers with greater socioeconomic resources may be more able to engage; those with livestock likely benefit from having their animals graze on organic matter from reduced tillage, and persons with higher formal education may be more equipped with the technical knowledge to implement such practices. Study 2 further tests the role of a future time perspective on behaviour, this time regarding the expected impact of climate change on agriculture. Unlike Study 1, Study 2 examined how *future* adaptation plans correspond to changes in weather patterns through a vignette experiment.

## STUDY 2

Having a future time perspective should help farmers plan for the effects of climate change by bringing possible futures into focus and increasing the salience of future outcomes. Study 2 used a repeated‐measures design to increase the salience of future weather conditions (i.e. rainfall is decreasing, increasing or staying the same), then measure how plans to adapt are affected, controlling for conservationist norms and socioeconomic factors. Further, we wanted to examine whether a more agriculture‐specific measure of future time perspective—concern for the effect of climate change on agriculture—could predict adaptation plans above and beyond conservationist norms, the presented changes in future weather conditions and socioeconomic resources.

Specific hypotheses were as follows:

1. Concern for the effect of climate change on agriculture will correlate with greater adaptation plans.
2. Socioeconomic factors correlate with greater adaptation plans.
3. Increased potential future conservation payments explain greater adaptation plans.
4. Increased salience of changes in rainfall explains greater adaptation plans.

### Materials and methods

#### Study procedure

This study focused on the same five US states as Study 1 (Ohio, Indiana, Illinois and the southern parts of Michigan and Wisconsin). A watershed was selected from each of these states located in agriculturally significant areas distributed along multiple land cover gradients (Figure S1). We then distributed the survey from August 2019 through October 2019 following the Tailored Design Method (Dillman et al., 2014) to farmers within the five watersheds, with two waves of surveys sent and three waves of reminders and the first full survey mailing included a $2 bill. After filtering out invalid contacts, there were 3452 farmers contacted and 918 returned usable surveys, for an adjusted response rate of about 27% (see Data S1 for a map of study region).

A full list of questions that were part of the survey is available in the Data S1. After being presented with the informed consent information, farmers answered questions about their farming operation. Next, they were presented with the vignette experiment. Following each scenario, participants indicated their plans to adapt, namely to increase their use of no‐till/conservation tillage (to promote soil health), increase their use of tile drainage (to manage too much water), increase their use of irrigation (to manage drought) and/or enrol more land in federal land conservation retirement programmes as a risk management or diversification strategy. After the vignette experiment, farmers answered the individual differences and demographic questions described below.

#### Materials

In the vignette experiment, rainfall levels were described to farmers as either decreasing, staying the same or increasing. Land conservation retirement payment levels either decreased or increased by $100 from baseline levels in the respondent's area. Details regarding the levels of each experiment parameter are summarized in Table 2.^2^ The full text of the vignette experiment is available in the Data S1.^3^

**TABLE 2:** Study 2 Vignette Experiment Parameters.

Parameter	Description	Coding
Conservation payment change	Change in conservation payment from baseline	0 = $100 less 1 = $100 more
Rain more	Change in rain. ‘10 inches more’ versus ‘no change’	*rain_more_* 1 = more 0 = no change, less rain
Rain less	Change in rain, ‘15 inches less’, versus ‘no change’	*rain_less_* 1 = less 0 = no change, more rain

*Note*: For the rainfall variables, because both are entered into the analyses simultaneously, the referent group is ‘no change’ in rainfall.

Operators were asked to ‘consider potential future weather conditions and indicate how you might adapt on your farm’. All participants were presented with three scenarios, which included a combination of the parameters shown in Table 2. The survey used a fixed orthogonal design to maximize efficiency for estimating the main effects of attributes and key interactions. After each, they were asked, ‘Under these conditions, what changes would you be likely to make to your farm operation?’. They then indicated what changes they would make from the list described earlier.^4^ The vignette experiment parameters were treated as categorical variables and coded as such for analyses (see Table 2). Scenario number was also controlled for in all analyses.

#### Measures

A full list of all measures included in the survey is in the Data S1. Farm operators indicated if they would engage in any adaptations in response to the conditions described in the vignette. Specifically, (1) enrolling more land in federal land retirement conservation programmes (2) increasing use of no‐till/conservation tillage (3) installing more tile drainage or (4) installing more irrigation. Unlike Study 1, data were not collected regarding the scale of adaptation, and thus forming the index variable was not possible.

Concern for the effects of changing weather conditions on one's farm operation was measured using 10 items developed for the survey (*α* = .931) that were designed to capture several changes that are likely to affect crop growing conditions. Operators were asked to indicate how concerned they were, from ‘Not at all concerned’ to ‘Very concerned’ about these potential impacts. Examples include more frequent flooding, fewer days for planting and increased soil erosion.

As in Study 1, formal education level, total cropland acres and presence of livestock assets (dichotomized) were examined as socioeconomic factors, as they indicate greater farmer and on‐farm socioeconomic resources.

For control variables, conservationist (*α* = .889) and productivist (*α* = .830) norms and yield, were measured identically as in Study 1. As in Study 1, farmers were also asked whether they rent land from others and their age. In addition, control variables also included soil type on the farmer's land, whether the farmer had insurance coverage, and baseline conservation payment amount in the farmer's state. These variables were included because clay soil tends to retain water during high rainfall events (Franzmeier et al., 2001), insurance coverage may be relied upon as an (albeit insufficient) risk management practice in itself (Fleckenstein et al., 2020) and because levels of conservation payments help explain programme enrollment (Lichtenberg, 2014).

#### Statistical method

Because of the repeated‐measures design, the vignette scenario observations were nested within participants, and participants were also nested within ‘group’, or the combination of vignette parameters that were observed. For each outcome variable of interest, a hierarchical mixed‐effects logistic regression was estimated. Fixed linear effects were estimated for all vignette experiment parameters, as well as vignette scenario number, to statistically control for order effects (e.g. the effect of repeated answering). Fixed effects were also estimated for concern for future weather effects, socioeconomic factors and all control variables. All continuous socioeconomic and control predictors were *z*‐scored to centre them at their mean and create meaningful zero‐values for the regression model. As a first step, all the variables were entered into the model. Then, to avoid model over‐identification and test the robustness of effects, non‐significant control variables were deleted from the model. In the reduced models, conclusions remained the same, except where noted in Tables 3, 4, 5, 6. Regression coefficients are reported in Tables 3, 4, 5, 6. All analyses were conducted using the ‘xtmelogit’ command in Stata version 13.1, which uses maximum likelihood estimation.

**TABLE 3:** Likelihood of Planning to Enrol More Land in Conservation Retirement Programme.

	Coefficient (log‐odds)	SE	*OR (*odds ratio)	*p*	95% C.I.	
					LL	UL
Increasing versus decreasing conservation payment	1.987***	0.202	7.295	<.001	1.591	2.383
Less rain versus baseline	0.817***	0.228	2.264	<.001	0.370	1.264
More rain versus baseline	0.418	0.226	1.519	.064	−0.024	0.860
Future weather concern	0.402***	0.088	1.495	<.001	0.229	0.574
Cropland acres	0.179	0.100	1.196	.074	−0.018	0.376
Formal education	0.312***	0.083	1.366	<.001	0.150	0.474
Livestock	−0.260	0.198	0.771	.190	−0.649	0.129
Intercept	−3.739***	0.415	0.024	<.001	−4.552	−2.926
Random intercept variability	0.246	0.126	1.134		0.090	0.671
Total observations	1547					

*Note*: *** indicates *p* < .001; ** indicates *p* < .01; * indicates *p* < .05. In the simplified model that includes only significant control variables as predictors, productivist norms drop below significance (*p* = .065). Area under receiver operating characteristic curve (ROC curve) = 0.829, SE = 0.014, 95% C.I. [0.812, 0.856].

**TABLE 4:** Likelihood of Planning to Increase No‐Till/Conservation Tillage.

	Coefficient (log‐odds)	SE	OR (odds ratio)	*p*	95% C.I.	
					LL	UL
Increasing versus decreasing conservation payment	−0.088	0.140	0.915	.528	−0.363	0.186
Less rain versus baseline	0.642***	0.174	1.901	<.001	0.301	0.983
More rain versus baseline	0.047	0.181	1.048	.793	−0.307	0.401
Future weather concern	0.395***	0.076	1.485	<.001	0.247	0.544
Cropland acres	0.221*	0.086	1.248	.010	0.054	0.389
Formal education	0.026	0.071	1.026	.714	−0.113	0.165
Livestock	0.435**	0.155	1.546	.005	0.132	0.739
Intercept	−1.491***	0.311	0.225	<.001	−2.099	−0.882
Random intercept variability	0.052	0.054	1.055		0.007	0.405
Total observations	1547					

*Note*: *** indicates *p* < .001; ** indicates *p* < .01; * indicates *p* < .05. Area under receiver operating characteristic curve (ROC curve) = 0.694, *SE* = 0.017, 95% C.I. [0.661, 0.726]. Because ROC bordered fair fit, a model that additionally included random intercept by the participant was estimated, which showed excellent fit. The conclusions from this model remain the same as those presented here, except for having livestock assets, which dropped below significance (*p* = .099) (area under ROC curve = 0.971, *SE* = 0.004, 95% C.I [0.964, 0.978]).

**TABLE 5:** Likelihood of Planning to Instal More Tile Drainage.

	Coefficient (log‐odds)	SE	*OR (*odds ratio)	*p*	95% C.I.	
					LL	UL
Increasing versus decreasing conservation payment	0.004	0.173	1.004	.979	−0.335	0.344
Less rain versus baseline	−1.034***	0.293	0.355	<.001	−1.609	−0.460
More rain versus baseline	0.983***	0.218	2.672	<.001	0.556	1.409
Future weather concern	0.180*	0.079	1.198	.022	0.026	0.335
Cropland acres	0.389***	0.092	1.475	<.001	0.209	0.568
Formal education	0.087	0.076	1.091	.249	−0.061	0.236
Livestock	0.143	0.171	1.154	.403	−0.192	0.478
Intercept	−3.776***	0.410	0.023	<.001	−4.580	−2.973
Random intercept variability	0.038	0.088	1.039		<0.001	3.478
Random slope variability: Less rain	0.614	0.446	1.847		0.148	2.551
Random slope variability: More rain	0.412	0.253	1.510		0.124	1.374
Total observations	1547					

*Note*: *** indicates *p* < .001; ** indicates *p* < .01; * indicates *p* < .05. In the simplified model that includes only significant control variables, age drops below significance (*p* = .051). Area under receiver operating characteristic curve (ROC curve) = 0.813, SE = 0.013, 95% C.I. [0.787, 0.838].

**TABLE 6:** Likelihood of Planning to Invest in Additional Irrigation.

	Coefficient (log‐odds)	SE	OR (odds ratio)	*p*	95% C.I.	
					LL	UL
Increasing versus decreasing conservation payment	−0.183	0.279	0.833	.512	−0.730	0.364
Less rain versus baseline	1.488***	0.367	4.426	<.001	0.768	2.207
More rain versus baseline	−0.264	0.472	0.768	.576	−1.190	0.662
Future weather concern	−0.040	0.144	0.961	.781	−0.322	0.242
Cropland acres	0.549**	0.170	1.732	.001	0.215	0.883
Formal education	0.602***	0.142	1.826	<.001	0.324	0.881
Livestock	0.467	0.295	1.595	.114	−0.112	1.046
Intercept	−3.756***	0.643	0.023	<.001	−5.015	−2.496
Random intercept variability	0.099	0.222	1.104		0.001	7.998
Total observations	1547					

*Note*: *** indicates *p* < .001; ** indicates *p* < .01, * indicates *p* < .05. Area under receiver operating characteristic curve (ROC curve) = 0.820, SE = 0.028, 95% C.I. [0.765, 0.874].

In each model, the random intercept of ‘group’ was estimated, as it was expected that the mean likelihood of engaging in each adaptation would differ based on the unique combination of factors presented across the three vignettes. A mixed‐effects regression analysis allows for the intercept of each group to be randomly estimated, thus accounting for variability in the intercept that would otherwise be attributed to error (Field & Wright, 2011).^5^ Likewise, it was reasoned that effects of the vignette experiment parameters (rainfall levels, conservation payments, etc.) may vary based on idiosyncrasies in the presented parameters to each group. Specifying random slopes estimates the slope of predictors separately for each group. In theory, one could estimate random slopes for all choice experiment parameters, but doing so created an estimating matrix that was computationally intractable. Thus, random slopes were estimated for choice experiment parameters with significant main effects. Notably, no significant random slopes were observed and are therefore omitted, except for the outcome variable of tile drainage.

### Results

#### Descriptive statistics

Farmers had a median farm size of 453 acres and average age of 63 years. The average formal education level among farmers was between ‘some college’ and ‘associate's degree’, and most (76%) did not own livestock, but most (66%) did rent land from others. Across the sample, mean concern for the impacts of changing weather patterns on local agricultural production was between ‘slightly concerned’ and ‘somewhat concerned’, with only 4% of the sample indicating that they were ‘not at all concerned’ (*M* = 1.762, SD = 0.938, scale range 0–4). Like Study 1, endorsement of conservationist norms was high in this sample (*M* = 2.945, SD = 0.639, scale range 0–4), whereas productivist norms were distributed around the scale mean (*M* = 2.120, SD = 0.743). A table of the descriptive statistics is available in the Data S1.

In the hierarchical mixed‐effects models, 1547 observations were included across the vignette experiment. These observations included data from 559 persons, who contributed an average of 2.8 observations per person (ranging from 1 to 3 observations per person).^6^

#### Model results

Consistent with Hypothesis 1, future weather concern was significantly correlated with the likelihood of multiple adaptations, except for irrigation (perhaps due to concerns being driven by too much rain as opposed to not enough). Relative to mean levels of future weather concern, operators with higher (1 SD above the mean) concern for the effects of future weather on agriculture were about 1.5 times as likely to plan to retire land and engage in more conservation tillage, and 1.2 times as likely to plan to install more tile drainage (see Tables 3, 4, 5, 6; for brevity, control parameters are not shown but are available in Data S1).

In partial support of Hypothesis 2, having more acres of cropland was associated with a higher likelihood of plans to adapt using more no‐till, tile drainage and irrigation. The largest impact was on irrigation, with larger farms 1.7 times as likely to plan to invest in additional irrigation. Operators with higher (1 SD above the mean) formal education were 1.4 times more likely to retire land, and 1.8 times more likely to plan to install more irrigation. Further, supporting Hypothesis 2, the likelihood of planning to increase conservation tillage was 1.5 times higher among operators with livestock.

Supporting Hypothesis 3, under conditions where a $100 increase in land conservation retirement payments from baseline was offered (as compared to a $100 decrease in payments), operators were 7.3 times as likely to plan to retire land.

Hypothesis 4 received limited support, as only *less* rainfall than usual explained plans to retire land and increase conservation tillage. Specifically, operators were 2.7 times more likely to plan to install tile drainage (which helps manage excess water) when rainfall increased. However, respondents were also 0.65 times less likely to plan to install tile drainage when rainfall decreased. Finally, farmers were 4.4 times more likely to plan to invest in additional irrigation when rainfall was lower compared to usual levels in their area.

### Study 2 discussion

In sum, having concern for the future effects of climate change on agriculture (our agriculture‐specific metric of future time perspective), and socioeconomic factors (farm size and operator education) were both relevant to adaptation plans. These effects remain significant above and beyond the effects of changing rainfall and land retirement payment conditions described in the vignette experiment. In addition, increasing payments, or the economic incentives, is a large driver of plans to adapt by retiring land and showed even larger effects than changing rainfall patterns. But importantly, even in the face of this strong economic effect, planning to adapt by retiring land was still impacted by concerns about the future, and to some degree, by resources (i.e. formal education level).

Thus, unlike in Study 1, thinking about the future predicted adaptation plans above and beyond conservationist norms. There are several possible explanations for this discrepancy. First, Study 2 used a measure of future thinking specific to agriculture, whereas Study 1 used a general measure of time perspective. Second, the measure used in Study 2 is one of *concern* about the future effects of climate change, which partly captures the risk perception of farmers in relation to climate change, in addition to future thinking. As a result, this measure is a more direct indicator of a lack of temporal discounting for the effects of climate change, which is the proximate means by which future time perspective impacts pro‐environmental behaviour (Keller et al., 2022; Milfont et al., 2012). Finally, it could be the case that thinking about the future is not sufficiently powerful to predict *actual* behaviours (measured in Study 1), only *planned* behaviours. Future work should explore whether domain‐specific tendencies to think about and plan for the future predict actual adaptation behaviours among farmers.

## GENERAL DISCUSSION

Whether socioeconomics is associated with adaptation, above and beyond the proposed socio‐psychological factors (e.g. future time perspective, conservationist norms), has important implications for designing policy and interventions to support adaptation. If it were the case that future time perspective (measured generally or specifically as future climate concern) completely explained the tendency to adapt, then policies should focus mostly on encouraging planning skills and drawing connections between adaptation practices and being prepared for future weather patterns. But if socioeconomic factors play an additional role, then policies would need to be designed that reduce current economic and other structural barriers to adoption.

Study 1 showed that there is no direct relationship between the tendency to plan for the future and adaptation among farmers. Instead, planners are more likely to believe that such practices are important for a good farmer to use. In turn, such conservationist norms predict more climate adaptation. Thus, encouraging farmers to develop adaptation plans for their farms must go beyond list‐making of actions to be taken. Instead, explicit language should be used that helps farmers develop a sense of the long‐term benefits of adaptation practices for their farm, and how specific practices can help reach crop production goals (e.g. increased water holding capacity for drought management).

The results of Study 1 also support that future time perspective may be an important contributor to how conservation norms might develop more generally, at least for contexts that require planning for future goals. It was reasoned that by tending to think about future goals and plan for how to achieve them, the impacts of climate change on one's crop production goals will be made more salient, as will the benefits of enacting conservation behaviours that adapt to changing climate. In turn, because these practices protect the growing operation, a farmer would come to value conservation, believe they have a personal responsibility to enact it and develop the injunctive norm that a ‘good farmer’ cares about conservation. The authors are aware of very few studies that have explored conservation norms as a mediator between future time perspective and behaviour. Recently, Olsen et al. (2023) found that placing greater importance on the future predicted higher biospheric *values*, and in turn, willingness to pay for environmentally‐friendly products. Similarly, Joireman and Liu (2014) found that concern for future consequences predicted environmental values, but indirectly through political orientation. However, Bruderer Enzler et al. (2019) found inconsistent support that environmental values mediate the relationship between concern for future consequences and energy use. Unlike these studies, the present study used a time perspective measure which focuses on taking planning steps for reaching goals (i.e. ‘When I want to achieve something, I set goals and consider specific means for reaching those goals’). As mentioned above, this was because climate adaptation requires thinking about how climate challenges may impact production goals, and how those challenges can be overcome by implementing complex changes to the farming operation. Future work should explore whether other aspects of future‐focused thinking, such as placing importance on the future, could also impact conservation norms.

Study 2 demonstrated that when the possible future impact of climate change on rainfall is made salient, adaptation plans increase. Expected changes in rainfall levels significantly altered plans to engage in all four adaptation practices examined. In addition, having baseline concern for the future impact of climate change on agricultural operations, a potential proxy for future time perspective was significantly associated with increased plans to engage in most adaptation strategies, even beyond conservationist norms.

Across both studies, the results also indicate that farmers with greater socioeconomic resources are more likely to adapt (controlling for the socio‐psychological factors). Having more cropland acres predicted adaptation plans for all but land retirement (i.e. no‐till, tile drainage and irrigation), while education was a significant predictor of land retirement and irrigation. Larger farms appear to have an advantage when it comes to no‐till practices, likely due to more favourable cost/benefit ratios for purchasing equipment, and greater time savings over a larger number of acres, compared to smaller farms. Larger farms also have an advantage in changing infrastructure, as there are more acres that can be used for drainage and irrigation. Finally, farmers with more formal education may have an advantage for practices that are less commonly used in their region, as was the case in the present studies regarding irrigation, as well as retiring land as part of an incentive programme. Implementing these adaptations would likely require engaging with local conservation districts and university‐run agricultural outreach offices, where more formally educated farmers are more connected (Drescher et al., 2017; Frisvold & Deva, 2012; Leib et al., 2002).

Accordingly, while increasing land retirement payments, communicating injunctive norms on conservation and focusing on the certainty of future impacts are likely to be necessary, they are also insufficient to engender adaptation planning. In addition, the resource‐related needs of farmers—whether that be technical or financial—must also be met. For example, the per acre cost to implement practices like tile drainage, irrigation and no‐till decreases as cropland increases (Prokopy et al., 2019). These costs can be offset through incentive reimbursement payments, but such programmes do not appeal to many operators with limited time and cognitive energy, due to paperwork burdens and the complexity of the reimbursement process (Reimer & Prokopy, 2014). In addition to incentive payments, subsidies for smaller farms that require no paperwork and more flexibility given the constraints of the operation, could be used. Current protests, in the UK and elsewhere, highlight how many farmers see the need for these programmes to help alleviate barriers as they plan for the future (Mason, 2024).

It is also important to consider the compounding effects of socioeconomic resources and climate stressors on the well‐being of farmers. The world over, many farmers already experience mental health challenges, and stress, depression and burnout are higher among farmers than non‐farmers (Hagen et al., 2019; O'Shaughnessy et al., 2022). Farmers report that climate stressors like drought and financial difficulties are major stressors to their mental health (Daghagh Yazd et al., 2019). These are factors that will likely worsen with climate change as changing weather patterns stress crops and yield. Mental health services can improve outcomes (Hagen et al., 2019), but alleviation or prevention of stressors is another crucial way forward.

### Limitations and future directions

In both studies, participation was voluntary, and participants were free to skip any questions they did not wish to answer. This resulted in missing data and possible self‐selection effects. In Study 1, participants were more likely to have missing data if they were higher in productivist norms. In Study 2, formal education predicted having participated in a greater number of vignettes. This means that, like with many survey studies (Zahl‐Thanem et al., 2021), persons with formal education are over‐represented in the data. Further, those who tend to focus more on production may have a lower interest in completing surveys. However, these concerns are mitigated somewhat by controlling for education and productivist norms when examining the significance of other predictors.

A possible mechanism by which future time perspective might increase on‐farm adaptations is through reduced temporal discounting. Temporal discounting is the tendency to downplay the benefits and consequences of actions or conditions that are further away in time (Baum & Easterling, 2010; Trope & Liberman, 2003). Research has shown that the greater the temporal distance of a problem, the more abstract the solutions seem, making it harder to act (Fujita et al., 2006; Liberman & Trope, 2008). But, when a person thinks about the future, the future is perceived to be less far away. This effect has been demonstrated in the context of climate change, where thinking about the future results in a decrease in temporal discounting, such that the perceived benefits of climate action are higher, as are the perceived consequences of climate change (Keller et al., 2022; Milfont et al., 2012). Study 2 did not directly measure temporal discounting, thus future research (including qualitative and mixed‐methods designs) should directly address whether this is a potential mechanism at play.

Although the scope of the present study examined a variety of adaptation practices, there are many other potential adaptation actions that a farmer could engage in to adapt to the impacts of climate change. For instance, investing in remote monitoring technology to track soil moisture, or planting more weather‐resistant seed varieties (Mase et al., 2017). We would expect that if farmers view a particular practice as relevant to adapting to changing weather patterns, and in turn believe they ought to be enacted, then the variables examined in this study (future time perspective, conservationist norms, concern about the future impacts of climate change) would be relevant predictors. Qualitative interviews could help identify which practices farmers do, and do not, believe have benefits for climate adaptation. Further, because many adaptations require the investment of capital resources, we would also expect that socioeconomic factors, in the absence of sufficient support for farmers, would lead to lower adaptation. Future research could be conducted to explicitly test these assumptions.

Finally, our test of statistical mediation in Study 1 was cross‐sectional in nature and thus is prone to the flaws of cross‐sectional designs, such as equivalent models (Pek & Hoyle, 2016). Regarding equivalent models, it was reasoned that it is implausible to hypothesize that domain‐specific injunctive norms towards conservation in farming could lead to the development of (domain non‐specific) future time perspective. Thus, this model was not examined. Nevertheless, a promising direction for future research would be to engage farmers in a direct manipulation of future time perspective. Such a study could test if future‐focused thinking can alter perceptions of what a ‘good farmer’ ought to do to protect their farm from the effects of climate change, and in turn, predict the implementation of those adaptations.

## CONCLUSION

Climate change will bring with it changes in temperature and rainfall that stress the resiliency of crops. Many stressors can be alleviated through adaptations that protect crops given increasing variability and weather extremes, as well as protect soil and water resources. The present study showed that having a future time perspective may increase injunctive norms that good farmers do conservation, which in turn predict adaptation through conservation behaviour. In a vignette experiment that described changes in weather patterns, the tendency to be concerned about future impacts of climate change on agriculture predicted greater adaptations above conservationist norms and the described changing weather patterns. Further, above and beyond these psychological factors, farmers of lower socioeconomic resources are especially vulnerable to these effects of climate change, as they are less likely to have already implemented adaptation strategies and less likely to plan to use them in the future, despite knowledge of changing rainfall conditions. The adaptation practices focused on in the present studies are important strategies in regions where climate change increases the risk of drought and flooding through rainfall extremes. Findings presented here show that practices should be encouraged through a multi‐method approach that: (1) clearly communicates the future benefits of farm management strategies for climate adaptation; (2) engages farmers by conveying the effects of changing weather patterns, especially rainfall, on agriculture on future growing seasons; (3) emphasize that by adapting to climate impacts, a ‘good farmer’ is protecting their growing operation; (4) support the financial, informational and technical resource needs of farmers in ways that minimize burdens to the farmer's time and effort, such as through subsidies or insurance rate reductions.

## AUTHOR CONTRIBUTIONS

**C. Dale Shaffer‐Morrison:** Conceptualization; data curation; formal analysis; software; visualization; writing – review and editing; writing – original draft. **Naseem H. Dillman‐Hasso:** Writing – review and editing; writing – original draft. **Robyn S. Wilson:** Conceptualization; funding acquisition; investigation; methodology; project administration; resources; writing – original draft; writing – review and editing; supervision.

## CONFLICT OF INTEREST STATEMENT

The authors declare no conflicts of interest.

## STATEMENT OF CONTRIBUTION

Conservationist norms are known to be related to the adoption of conservation practices, but how these norms develop among farmers requires more research. We find that, like other actions which target future concerns, future time perspective explains greater conservationist norms among farmers. A mediation model indicated that conservationist norms mediate the relationship between time perspective and actual adaptation practices. Manipulating future time perspective resulted in a greater willingness to adapt through conservation practices.

## Supporting information


**Data S1.** Supplementary Information.

## Data Availability

The data and analyses that support these findings, as well as Data S1, are available on the Open Science Framework at https://osf.io/8hdpt/.

## Footnotes

^1^
In a logistic regression analysis predicting whether participants were included in this analysis, only average productivist identity was significant; persons with higher productivist identity were more likely to have missing data (OR = 0.715, SE = 0.120, *p* = .045, 95% C.I. [0.516, 0.993]).

^2^
Table 2 includes parameters most relevant to the hypotheses of this manuscript. During the study, participants were randomly presented with one of 36 overall combinations in order to reduce the complexity (e.g. matrix size) of the hierarchical mixed‐effects regression. The composition of these combinations was determined using the software Sawtooth. The full list of conditions is available in the Data S1. The results presented are those of the full model, controlling for all parameters.

^3^
Also included in the vignette experiment parameters were planting and harvesting dates, as well as the likelihood of the weather conditions occurring over the next 10 years. While it is objectively true that it is difficult to predict how climate change will affect weather patterns in any given year, this latter parameter was intended to test how a farmer's *perceived surety* of weather changes affects adaptation intentions. However, this parameter exhibited little effect on outcomes and thus is not reported here. Growing times were included in order to examine the effects on crop insurance plans and plans to engage in double‐cropping. Because these outcomes are not of interest to the purpose of this manuscript, their results are not reported here, although they were controlled for in all analyses. Their results can be reproduced using the provided code file.

^4^
Because this survey was part of a larger sustainability modelling effort, additional management options were asked that relate to the food‐energy‐water nexus, which do not relate to soil conservation practices or water management, and are therefore not included here. These additional practices include double cropping, changing insurance coverage, and taking no action.

^5^
It is also possible in hierarchical mixed‐effects models to estimate random slopes and intercepts that vary randomly by participant. However, doing so creates a very large matrix that is computationally intractable for logistic distributions (e.g. when the dependent variable is binary).

^6^
In a Poisson regression predicting the number of vignette scenarios that the participant did not engage with, only the level of formal education predicted engagement. Those with higher formal education participated in a greater number of vignettes.

## References

[bjso12850-bib-0001] Agriculture and Agri‐Food Canada . (2022). Soil Health in Canada. https://agriculture.canada.ca/en/international‐trade/market‐intelligence/soil‐health‐canada

[bjso12850-bib-0002] Anang, B. T. , Bäckman, S. , & Sipiläinen, T. (2020). Adoption and income effects of agricultural extension in northern Ghana. Scientific African, 7, e00219. 10.1016/j.sciaf.2019.e00219

[bjso12850-bib-0003] Arbuckle, J. G. (2013). Farmer support for extending conservation compliance beyond soil erosion: Evidence from Iowa. Journal of Soil and Water Conservation, 68(2), 99–109. 10.2489/jswc.68.2.99

[bjso12850-bib-0004] Arbuckle, J. G. , Morton, L. W. , & Hobbs, J. (2015). Understanding farmer perspectives on climate change adaptation and mitigation: The roles of trust in sources of climate information, climate change beliefs, and perceived risk. Environment and Behavior, 47(2), 205–234. 10.1177/0013916513503832 25983336 PMC4359208

[bjso12850-bib-0005] Arnocky, S. , Milfont, T. L. , & Nicol, J. R. (2014). Time perspective and sustainable behavior: Evidence for the distinction between consideration of immediate and future consequences. Environment and Behavior, 46(5), 556–582. 10.1177/0013916512474987

[bjso12850-bib-0006] Azadi, Y. , Yazdanpanah, M. , & Mahmoudi, H. (2019). Understanding smallholder farmers' adaptation behaviors through climate change beliefs, risk perception, trust, and psychological distance: Evidence from wheat growers in Iran. Journal of Environmental Management, 250, 109456. 10.1016/j.jenvman.2019.109456 31513997

[bjso12850-bib-0007] Baird, H. M. , Webb, T. L. , Sirois, F. M. , & Gibson‐Miller, J. (2021). Understanding the effects of time perspective: A meta‐analysis testing a self‐regulatory framework. Psychological Bulletin, 147, 233–267. 10.1037/bul0000313 33180512

[bjso12850-bib-0008] Baum, S. D. , & Easterling, W. E. (2010). Space‐time discounting in climate change adaptation. Mitigation and Adaptation Strategies for Global Change, 15(6), 591–609. 10.1007/s11027-010-9239-9

[bjso12850-bib-0009] Below, T. B. , Mutabazi, K. D. , Kirschke, D. , Franke, C. , Sieber, S. , Siebert, R. , & Tscherning, K. (2012). Can farmers' adaptation to climate change be explained by socio‐economic household‐level variables? Global Environmental Change, 22(1), 223–235. 10.1016/j.gloenvcha.2011.11.012

[bjso12850-bib-0010] Berry, H. L. , Hogan, A. , Owen, J. , Rickwood, D. , & Fragar, L. (2011). Climate change and farmers' mental health: Risks and responses. Asia‐Pacific Journal of Public Health, 23, 119S–132S. 10.1177/1010539510392556 21447547

[bjso12850-bib-0011] Bruderer Enzler, H. , Diekmann, A. , & Liebe, U. (2019). Do environmental concern and future orientation predict metered household electricity use? Journal of Environmental Psychology, 62, 22–29. 10.1016/j.jenvp.2019.02.004

[bjso12850-bib-0012] Cialdini, R. B. , Reno, R. R. , & Kallgren, C. A. (1990). A focus theory of normative conduct: Recycling the concept of norms to reduce littering in public places. Journal of Personality and Social Psychology, 58, 1015–1026. 10.1037/0022-3514.58.6.1015

[bjso12850-bib-0013] Conway, S. F. , McDonagh, J. , Farrell, M. , & Kinsella, A. (2021). Going against the grain: Unravelling the habitus of older farmers to help facilitate generational renewal in agriculture. Sociologia Ruralis, 61(3), 602–622. 10.1111/soru.12355

[bjso12850-bib-0014] Corral‐Verdugo, V. , Fraijo‐Sing, B. , & Pinheiro, J. Q. (2006). Sustainable behavior and time perspective: Present, past, and future orientations and their relationship with water conservation behavior. Revista Interamericana de Psicología, 40, 139–147.

[bjso12850-bib-0015] Daghagh Yazd, S. , Wheeler, S. A. , & Zuo, A. (2019). Key risk factors affecting Farmers' mental health: A systematic review. International journal of environmental research and public health, 16(23), 4849. 10.3390/ijerph16234849 PMC6926562 31810320

[bjso12850-bib-0016] Deines, J. M. , Wang, S. , & Lobell, D. B. (2019). Satellites reveal a small positive yield effect from conservation tillage across the US Corn Belt. Environmental Research Letters, 14(12), 124038. 10.1088/1748-9326/ab503b

[bjso12850-bib-0017] Dillman, D. A. , Smyth, J. D. , & Christian, L. M. (2014). Internet, phone, mail, and mixed‐mode surveys: The tailored design method. John Wiley & Sons.

[bjso12850-bib-0018] Drescher, M. , Warriner, G. K. , Farmer, J. R. , & Larson, B. M. H. (2017). Private landowners and environmental conservation: A case study of socialpsychological determinants of conservation program participation in Ontario. Ecology and Society, 22(1), 44. 10.5751/ES-09118-220144

[bjso12850-bib-0019] Drugova, T. , Curtis, K. R. , & Ward, R. A. (2022). Producer preferences for drought management strategies in the arid west. Renewable Agriculture and Food Systems, 37(1), 14–23. 10.1017/S1742170521000259

[bjso12850-bib-0020] Du, W. , Green, L. , & Myerson, J. (2002). Cross‐cultural comparisons of discounting delayed and probabilistic rewards. The. Psychological Record, 52(4), 479–492.

[bjso12850-bib-0021] Farr, M. , Eagle, L. , & Hay, R. (2018). Key determinants of pro‐environmental behaviour of land managers in the agricultural sector: Literature review. Report preapred for National Environmental Science Program. Reef and Rainforest Research Centre Limited.

[bjso12850-bib-0022] Field, A. P. , & Wright, D. B. (2011). A primer on using multilevel models in clinical and experimental psychopathology research. Journal of Experimental Psychopathology, 2(2), 271–293. 10.5127/jep.013711

[bjso12850-bib-0023] Fleckenstein, M. , Lythgoe, A. , Lu, J. , Thompson, N. , Doering, O. , Harden, S. , Getson, J. M. , & Prokopy, L. (2020). Crop insurance: A barrier to conservation adoption? Journal of Environmental Management, 276, 111223. 10.1016/j.jenvman.2020.111223 32891982

[bjso12850-bib-0024] Franzmeier, D. , Kladivko, E. , & Jenkinson, B. (2001). Drainage and wet soil management: Wet soils of Indiana. Purdue University.

[bjso12850-bib-0025] Frisvold, G. B. , & Deva, S. (2012). Farm size, irrigation practices, and conservation program participation in the us southwest. Irrigation and Drainage, 61(5), 569–582. 10.1002/ird.1676

[bjso12850-bib-0026] Fujita, K. , Henderson, M. D. , Eng, J. , Trope, Y. , & Liberman, N. (2006). Spatial distance and mental construal of social events. Psychological Science, 17(4), 278–282. 10.1111/j.1467-9280.2006.01698.x 16623682

[bjso12850-bib-0027] Gao, L. , & Arbuckle, J. (2022). Examining farmers' adoption of nutrient management best management practices: A social cognitive framework. Agriculture and Human Values, 39(2), 535–553. 10.1007/s10460-021-10266-2

[bjso12850-bib-0028] Gaudin, A. C. , Tolhurst, T. N. , Ker, A. P. , Janovicek, K. , Tortora, C. , Martin, R. C. , & Deen, W. (2015). Increasing crop diversity mitigates weather variations and improves yield stability. PLoS One, 10(2), e0113261. 10.1371/journal.pone.0113261 25658914 PMC4320064

[bjso12850-bib-0029] Gbetibouo, G. A. , Hassan, R. M. , & Ringler, C. (2010). Modelling farmers' adaptation strategies for climate change and variability: The case of the Limpopo Basin, South Africa. Agrekon, 49(2), 217–234.

[bjso12850-bib-0030] Gil, J. D. , Daioglou, V. , van Ittersum, M. , Reidsma, P. , Doelman, J. C. , van Middelaar, C. E. , & van Vuuren, D. P. (2019). Reconciling global sustainability targets and local action for food production and climate change mitigation. Global Environmental Change, 59, 101983.

[bjso12850-bib-0031] Göllner, L. M. , Ballhausen, N. , Kliegel, M. , & Forstmeier, S. (2018). Delay of gratification, delay discounting and their associations with age, episodic future thinking, and future time perspective. Frontiers in Psychology, 8, 2304. 10.3389/fpsyg.2017.02304 29422875 PMC5788968

[bjso12850-bib-0032] Hagen, B. N. M. , Albright, A. , Sargeant, J. , Winder, C. B. , Harper, S. L. , O'Sullivan, T. L. , & Jones‐Bitton, A. (2019). Research trends in farmers' mental health: A scoping review of mental health outcomes and interventions among farming populations worldwide. PLoS One, 14(12), e0225661. 10.1371/journal.pone.0225661 31805070 PMC6894791

[bjso12850-bib-0033] Hornsey, M. J. , Harris, E. A. , Bain, P. G. , & Fielding, K. S. (2016). Meta‐analyses of the determinants and outcomes of belief in climate change. Nature Climate Change, 6, 622–626. 10.1038/nclimate2943

[bjso12850-bib-0034] Houser, M. , Gazley, B. , Reynolds, H. , Grennan Browning, E. , Sandweiss, E. , & Shanahan, J. (2022). Public support for local adaptation policy: The role of social‐psychological factors, perceived climatic stimuli, and social structural characteristics. Global Environmental Change, 72, 102424. 10.1016/j.gloenvcha.2021.102424

[bjso12850-bib-0035] Inter‐American Institute for Cooperation on Agriculture . (2021). Brazil launches the living soils of the Americas program, the restoration initiative promoted by IICA and renowned scientist Rattan Lal. IICA. https://iica.int/es/press/noticias/brasil‐lanza‐el‐programa‐suelos‐vivos‐de‐las‐americas‐la‐iniciativa‐de‐restauracion/

[bjso12850-bib-0036] Joireman, J. , & Liu, R. L. (2014). Future‐oriented women will pay to reduce global warming: Mediation via political orientation, environmental values, and belief in global warming. Journal of Environmental Psychology, 40, 391–400.

[bjso12850-bib-0037] Joireman, J. A. , Van Lange, P. A. M. , & Van Vugt, M. (2004). Who cares about the environmental impact of cars?: Those with an eye toward the future. Environment and Behavior, 36(2), 187–206.

[bjso12850-bib-0038] Kassam, A. , Friedrich, T. , & Derpsch, R. (2019). Global spread of conservation agriculture. International Journal of Environmental Studies, 76(1), 29–51. 10.1080/00207233.2018.1494927

[bjso12850-bib-0039] Kast, J. B. , Kalcic, M. , Wilson, R. , Jackson‐Smith, D. , Breyfogle, N. , & Martin, J. (2021). Evaluating the efficacy of targeting options for conservation practice adoption on watershed‐scale phosphorus reductions. Water Research, 201, 117375. 10.1016/j.watres.2021.117375 34218088

[bjso12850-bib-0040] Keller, E. , Marsh, J. E. , Richardson, B. H. , & Ball, L. J. (2022). A systematic review of the psychological distance of climate change: Towards the development of an evidence‐based construct. Journal of Environmental Psychology, 81, 101822. 10.1016/j.jenvp.2022.101822

[bjso12850-bib-0041] Khatri‐Chhetri, A. , Junior, C. C. , & Wollenberg, E. (2022). Greenhouse gas mitigation co‐benefits across the global agricultural development programs. Global Environmental Change, 76, 102586.

[bjso12850-bib-0042] Kim, S. H. , & Seock, Y.‐K. (2019). The roles of values and social norm on personal norms and pro‐environmentally friendly apparel product purchasing behavior: The mediating role of personal norms. Journal of Retailing and Consumer Services, 51, 83–90. 10.1016/j.jretconser.2019.05.023

[bjso12850-bib-0043] Klöckner, C. A. (2013). A comprehensive model of the psychology of environmental behaviour—A meta‐analysis. Global Environmental Change, 23(5), 1028–1038. 10.1016/j.gloenvcha.2013.05.014

[bjso12850-bib-0044] Knowler, D. , & Bradshaw, B. (2007). Farmers' adoption of conservation agriculture: A review and synthesis of recent research. Food Policy, 32(1), 25–48. 10.1016/j.foodpol.2006.01.003

[bjso12850-bib-0045] Kooij, D. T. , Kanfer, R. , Betts, M. , & Rudolph, C. W. (2018). Future time perspective: A systematic review and meta‐analysis. Journal of Applied Psychology, 103(8), 867–893. 29683685 10.1037/apl0000306

[bjso12850-bib-0046] Krah, K. , Michelson, H. , Perge, E. , & Jindal, R. (2019). Constraints to adopting soil fertility management practices in Malawi: A choice experiment approach. World Development, 124, 104651.

[bjso12850-bib-0047] Leib, B. G. , Hattendorf, M. , Elliott, T. , & Matthews, G. (2002). Adoption and adaptation of scientific irrigation scheduling: Trends from Washington, USA as of 1998. Agricultural Water Management, 55(2), 105–120. 10.1016/S0378-3774(01)00191-3

[bjso12850-bib-0048] Liao, C. , Nolte, K. , Brown, D. G. , Lay, J. , & Agrawal, A. (2023). The carbon cost of agricultural production in the global land rush. Global Environmental Change, 80, 102679.

[bjso12850-bib-0049] Liberman, N. , & Trope, Y. (2008). The psychology of transcending the here and now. Science, 322(5905), 1201–1205. 10.1126/science.1161958 19023074 PMC2643344

[bjso12850-bib-0050] Lichtenberg, E. (2014). Conservation, the farm bill, and US agri‐environmental policy. Choices, 29(3), 1–6.

[bjso12850-bib-0051] Liu, T. , Bruins, R. J. F. , & Heberling, M. T. (2018). Factors influencing Farmers' adoption of best management practices: A review and synthesis. Sustainability, 10(2), 432. 10.3390/su10020432 29682334 PMC5907504

[bjso12850-bib-0052] Lu, J. , Ranjan, P. , Floress, K. , Arbuckle, J. G. , Church, S. P. , Eanes, F. R. , Gao, Y. , Gramig, B. M. , Singh, A. S. , & Prokopy, L. S. (2022). A meta‐analysis of agricultural conservation intentions, behaviors, and practices: Insights from 35 years of quantitative literature in the United States. Journal of Environmental Management, 323, 116240. 10.1016/j.jenvman.2022.116240 36261983

[bjso12850-bib-0053] Mase, A. S. , Gramig, B. M. , & Prokopy, L. S. (2017). Climate change beliefs, risk perceptions, and adaptation behavior among Midwestern U.S. crop farmers. Climate Risk Management, 15, 8–17. 10.1016/j.crm.2016.11.004

[bjso12850-bib-0054] Mason, C. (2024). Farmers Protest against Inheritance Tax Changes. https://www.bbc.com/news/articles/c62jdz61j3yo

[bjso12850-bib-0055] McGuire, J. M. , Morton, L. W. , Arbuckle, J. G. , & Cast, A. D. (2015). Farmer identities and responses to the social–biophysical environment. Journal of Rural Studies, 39, 145–155.

[bjso12850-bib-0056] McGuire, J. M. , Morton, L. W. , & Cast, A. D. (2013). Reconstructing the good farmer identity: Shifts in farmer identities and farm management practices to improve water quality. Agriculture and Human Values, 30(1), 57–69.

[bjso12850-bib-0057] McLellan, E. L. , Schilling, K. E. , Wolter, C. F. , Tomer, M. D. , Porter, S. A. , Magner, J. A. , Smith, D. R. , & Prokopy, L. S. (2018). Right practice, right place: A conservation planning toolbox for meeting water quality goals in the Corn Belt. Journal of Soil and Water Conservation, 73(2), 29A–34A. 10.2489/jswc.73.2.29A

[bjso12850-bib-0058] Mehmetoglu, M. (2018). Medsem: A stata package for statistical mediation analysis. International Journal of Computational Economics and Econometrics, 8(1), 63–78.

[bjso12850-bib-0059] Melillo, J. M. , Richmond, T. T. , & Yohe, G. (2014). Climate change impacts in the United States: The third National Climate Assessment. Global Change Research Program. 10.7930/J0Z31WJ2

[bjso12850-bib-0060] Milfont, T. L. , Wilson, J. , & Diniz, P. (2012). Time perspective and environmental engagement: A meta‐analysis. International Journal of Psychology, 47(5), 325–334. 10.1080/00207594.2011.647029 22452746

[bjso12850-bib-0061] Mirzabaev, A. , Kerr, R. B. , Hasegawa, T. , Pradhan, P. , Wreford, A. , von der Pahlen, M. C. T. , & Gurney‐Smith, H. (2023). Severe climate change risks to food security and nutrition. Climate Risk Management, 39, 100473.

[bjso12850-bib-0062] Morgan, M. I. , Hine, D. W. , Bhullar, N. , & Loi, N. M. (2015). Landholder adoption of low emission agricultural practices: A profiling approach. Journal of Environmental Psychology, 41, 35–44. 10.1016/j.jenvp.2014.11.004

[bjso12850-bib-0063] Morris, C. , & Arbuckle, J. G. (2021). Conservation plans and soil and water conservation practice use: Evidence from Iowa. Journal of Soil and Water Conservation, 76, 457–471. 10.2489/jswc.2021.00166

[bjso12850-bib-0064] Mouro, C. , & Duarte, A. P. (2021). Organisational climate and pro‐environmental Behaviours at work: The mediating role of personal norms. Frontiers in Psychology, 12, 635739. 10.3389/fpsyg.2021.635739 34621204 PMC8490716

[bjso12850-bib-0065] Nielsen, K. S. , Cologna, V. , Lange, F. , Brick, C. , & Stern, P. C. (2021). The case for impact‐focused environmental psychology. Journal of Environmental Psychology, 74, 101559.

[bjso12850-bib-0066] Niemiec, R. M. , Champine, V. , Vaske, J. J. , & Mertens, A. (2020). Does the impact of norms vary by type of norm and type of conservation behavior? a meta‐analysis. Society and Natural Resources, 33(8), 1024–1040.

[bjso12850-bib-0067] Odum, A. L. , Becker, R. J. , Haynes, J. M. , Galizio, A. , Frye, C. C. , Downey, H. , Friedel, J. E. , & Perez, D. (2020). Delay discounting of different outcomes: Review and theory. Journal of the Experimental Analysis of Behavior, 113(3), 657–679. 32147840 10.1002/jeab.589 PMC7373228

[bjso12850-bib-0068] Ojumu, O. , Ojumu, M. F. , & Joonas, K. (2020). A theoretical framework for assessing the impact of climate change on crop yields. AIMS International Journal of Management, 14(2), 65–87.

[bjso12850-bib-0069] Olsen, S. O. , & Tuu, H. H. (2021). The relationships between core values, food‐specific future time perspective and sustainable food consumption. Sustainable Production and Consumption, 26, 469–479. 10.1016/j.spc.2020.12.019

[bjso12850-bib-0070] Olsen, S. O. , Tuu, H. H. , & Tudoran, A. A. (2023). Comparing time focus with time importance for measuring future time perspectives in the context of pro‐environmental values and outcomes. Frontiers in Psychology, 14, 945487. 37089737 10.3389/fpsyg.2023.945487 PMC10114413

[bjso12850-bib-0071] O'Shaughnessy, B. R. , O'Hagan, A. D. , Burke, A. , McNamara, J. , & O'Connor, S. (2022). The prevalence of farmer burnout: Systematic review and narrative synthesis. Journal of Rural Studies, 96, 282–292. 10.1016/j.jrurstud.2022.11.002

[bjso12850-bib-0072] Owino, V. , Kumwenda, C. , Ekesa, B. , Parker, M. E. , Ewoldt, L. , Roos, N. , Lee, W. T. , & Tome, D. (2022). The impact of climate change on food systems, diet quality, nutrition, and health outcomes: A narrative review. Frontiers in Climate, 4, 941842.

[bjso12850-bib-0073] Padawer, E. A. , Jacobs‐Lawson, J. M. , Hershey, D. A. , & Thomas, D. G. (2007). Demographic indicators as predictors of future time perspective. Current Psychology, 26(2), 102–108. 10.1007/s12144-007-9008-4

[bjso12850-bib-0074] Palm, C. , Blanco‐Canqui, H. , DeClerck, F. , Gatere, L. , & Grace, P. (2014). Conservation agriculture and ecosystem services: An overview. Agriculture, Ecosystems & Environment, 187, 87–105. 10.1016/j.agee.2013.10.010

[bjso12850-bib-0075] Pek, J. , & Hoyle, R. H. (2016). On the (in) validity of tests of simple mediation: Threats and solutions. Social and Personality Psychology Compass, 10(3), 150–163. 26985234 10.1111/spc3.12237 PMC4789289

[bjso12850-bib-0076] Perry, V. , & Davenport, M. A. (2020). An inductive framework of self‐efficacy to understand and support farmers in conservation agriculture. Journal of Soil and Water Conservation, 75(2), 198–208. 10.2489/jswc.75.2.198

[bjso12850-bib-0077] Piñeiro, V. , Arias, J. , Dürr, J. , Elverdin, P. , Ibáñez, A. M. , Kinengyere, A. , Opazo, C. M. , Owoo, N. , Page, J. R. , Prager, S. D. , & Torero, M. (2020). A scoping review on incentives for adoption of sustainable agricultural practices and their outcomes. Nature Sustainability, 3(10), 809–820. 10.1038/s41893-020-00617-y

[bjso12850-bib-0078] Prokopy, L. S. , Floress, K. , Arbuckle, J. G. , Church, S. P. , Eanes, F. R. , Gao, Y. , Gramig, B. M. , Ranjan, P. , & Singh, A. S. (2019). Adoption of agricultural conservation practices in the United States: Evidence from 35 years of quantitative literature. Journal of Soil and Water Conservation, 74(5), 520–534. 10.2489/jswc.74.5.520

[bjso12850-bib-0079] Ranjan, P. , Church, S. P. , Floress, K. , & Prokopy, L. S. (2019). Synthesizing conservation motivations and barriers: What have we learned from qualitative studies of Farmers' behaviors in the United States? Society & Natural Resources, 32(11), 1171–1199. 10.1080/08941920.2019.1648710

[bjso12850-bib-0080] Reimer, A. P. , & Prokopy, L. S. (2014). Farmer participation in U.S. farm bill conservation programs. Environmental Management, 53(2), 318–332. 10.1007/s00267-013-0184-8 24114348

[bjso12850-bib-0081] Ribaudo, M. (2015). The limits of voluntary conservation programs. Choices, 30(2), 1–5.

[bjso12850-bib-0082] Roesch‐McNally, G. E. , Arbuckle, J. , & Tyndall, J. C. (2018). Barriers to implementing climate resilient agricultural strategies: The case of crop diversification in the US Corn Belt. Global Environmental Change, 48, 206–215. 10.1016/j.gloenvcha.2017.12.002

[bjso12850-bib-0083] Roesch‐McNally, G. E. , Gordon Arbuckle, J. , & Tyndall, J. C. (2017). What would farmers do? Adaptation intentions under a Corn Belt climate change scenario. Agriculture and Human Values, 34(2), 333–346. 10.1007/s10460-016-9719-y

[bjso12850-bib-0084] Schattman, R. E. , Caswell, M. , & Faulkner, J. W. (2021). Eyes on the horizon: Temporal and social perspectives of climate risk and agricultural decision making among climate‐informed farmers. Society & Natural Resources, 34(6), 765–784.

[bjso12850-bib-0085] Schoolman, E. D. , & Arbuckle, J. G. (2022). Cover crops and specialty crop agriculture: Exploring cover crop use among vegetable and fruit growers in Michigan and Ohio. Journal of Soil and Water Conservation, 77(4), 403–417. 10.2489/jswc.2022.00006

[bjso12850-bib-0086] Schreiber, J. B. (2008). Core reporting practices in structural equation modeling. Research in Social and Administrative Pharmacy, 4(2), 83–97. 18555963 10.1016/j.sapharm.2007.04.003

[bjso12850-bib-0087] Schreiber, J. B. , Nora, A. , Stage, F. K. , Barlow, E. A. , & King, J. (2006). Reporting structural equation modeling and confirmatory factor analysis results: A review. The Journal of Educational Research, 99(6), 323–338. 10.3200/JOER.99.6.323-338

[bjso12850-bib-0088] Segev, S. , & Liu, Y. (2022). The effect of temporal orientation on Green purchase behavior: Comparing U.S. and Chinese consumers. Journal of International Consumer Marketing, 34(1), 95–109. 10.1080/08961530.2021.1917034

[bjso12850-bib-0089] Shaffer‐Morrison, C. D. , & Wilson, R. S. (2024). The nutrient reduction index: A minimalist and continuous measure of conservation practice adoption among farmers. Journal of Soil and Water Conservation, 79(1), 1–7.

[bjso12850-bib-0090] Shariatzadeh, M. , & Bijani, M. (2022). Towards farmers' adaptation to climate change: The effect of time perspective. Journal of Cleaner Production, 348, 131284. 10.1016/j.jclepro.2022.131284

[bjso12850-bib-0091] Sircova, A. , Van De Vijver, F. J. , Osin, E. , Milfont, T. L. , Fieulaine, N. , Kislali‐Erginbilgic, A. , & Boyd, J. N. (2014). A global look at time: A 24‐country study of the equivalence of the Zimbardo time perspective inventory. SAGE Open, 4(1), 2158244013515686.

[bjso12850-bib-0092] Stagnari, F. , Ramazzotti, S. , & Pisante, M. (2010). Conservation agriculture: A different approach for crop production through sustainable soil and water management: A review (pp. 55–83). Organic Farming, Pest Control and Remediation of Soil Pollutants.

[bjso12850-bib-0093] StataCorp . (2013). Stata statistical software: Release 13 (version 13) [Computer software]. StataCorp LP.

[bjso12850-bib-0094] Stern, P. C. (2002). New environmental theories: Toward a coherent theory of environmentally significant behavior. Journal of Social Issues, 56(3), 407–424. 10.1111/0022-4537.00175

[bjso12850-bib-0095] Stern, P. C. , Dietz, T. , Abel, T. , Guagnano, G. A. , & Kalof, L. (1999). A value‐belief‐norm theory of support for social movements: The case of environmentalism. Human Ecology Review, 6(2), 81–97.

[bjso12850-bib-0096] Stolarski, M. , Bitner, J. , & Zimbardo, P. G. (2011). Time perspective, emotional intelligence and discounting of delayed awards. Time & Society, 20(3), 346–363. 10.1177/0961463X11414296

[bjso12850-bib-0097] Sumaryanto Susilowati, S. H. , Nurfatriani, F. , Tarigan, H. , Erwidodo Sudaryanto, T. , & Perkasa, H. W. (2022). Determinants of Farmers' behavior towards land conservation practices in the upper Citarum watershed in West Java, Indonesia. Lands, 11(10), 1827.

[bjso12850-bib-0098] Trope, Y. , & Liberman, N. (2003). Temporal construal. Psychological Review, 110, 403–421. 10.1037/0033-295X.110.3.403 12885109

[bjso12850-bib-0099] UN General Assembly . (2015). Transforming our world: The 2030 agenda for sustainable development. (Resolution No. Resolution A/RES/70/1, Adopted by the General Assembly on 25 September 2015).

[bjso12850-bib-0100] Wens, M. L. K. , van Loon, A. F. , Veldkamp, T. I. E. , & Aerts, J. C. J. H. (2022). Education, financial aid, and awareness can reduce smallholder farmers' vulnerability to drought under climate change. Natural Hazards and Earth System Sciences, 22(4), 1201–1232. 10.5194/nhess-22-1201-2022

[bjso12850-bib-0101] West, S. G. , Taylor, A. B. , & Wu, W. (2012). Model fit and model selection in structural equation modeling. In Handbook of structural equation modeling (pp. 209–231). The Guilford Press.

[bjso12850-bib-0102] Wezel, A. , Casagrande, M. , Celette, F. , Vian, J.‐F. , Ferrer, A. , & Peigné, J. (2014). Agroecological practices for sustainable agriculture. a review. Agronomy for Sustainable Development, 34(1), 1–20. 10.1007/s13593-013-0180-7

[bjso12850-bib-0103] Wilson, R. S. , Herziger, A. , Hamilton, M. , & Brooks, J. S. (2020). From incremental to transformative adaptation in individual responses to climate‐exacerbated hazards. Nature Climate Change, 10(3), 200–208. 10.1038/s41558-020-0691-6

[bjso12850-bib-0104] Ye, J.‐Y. , Ding, Q.‐Y. , Cui, J.‐F. , Liu, Z. , Jia, L.‐X. , Qin, X.‐J. , Xu, H. , & Wang, Y. (2022). A meta‐analysis of the effects of episodic future thinking on delay discounting. Quarterly Journal of Experimental Psychology, 75(10), 1876–1891. 10.1177/17470218211066282 34841982

[bjso12850-bib-0105] Zahl‐Thanem, A. , Burton, R. J. F. , & Vik, J. (2021). Should we use email for farm surveys? A comparative study of email and postal survey response rate and non‐response bias. Journal of Rural Studies, 87, 352–360. 10.1016/j.jrurstud.2021.09.029

[bjso12850-bib-0106] Zhang, L. , Ruiz‐Menjivar, J. , Luo, B. , Liang, Z. , & Swisher, M. E. (2020). Predicting climate change mitigation and adaptation behaviors in agricultural production: A comparison of the theory of planned behavior and the value‐belief‐norm theory. Journal of Environmental Psychology, 68, 101408. 10.1016/j.jenvp.2020.101408

[bjso12850-bib-0107] Zhao, X. , Lynch, J. G., Jr. , & Chen, Q. (2010). Reconsidering baron and Kenny: Myths and truths about mediation analysis. Journal of Consumer Research, 37, 197–206. 10.1086/651257

[bjso12850-bib-0108] Zimbardo, P. G. , & Boyd, J. N. (1999). Putting time in perspective: A valid, reliable individual‐differences metric. Journal of Personality and Social Psychology, 77, 1271–1288.

