# Supplementary material for: Sowing seeds for the future: Future time perspective and climate adaptation among farmers
Source: Br J Soc Psychol. 2025 Feb 6;64(2):e12850. doi: 10.1111/bjso.12850 (PMC11800754; doi:10.1111/bjso.12850)
Supplement: Supplementary file 1 — Data S1. Supplementary Information. [file BJSO-64-0-s001.docx]

Supporting Information

Table of Contents

[1. Study 1 Supporting Information 3](#_Toc164948115)

[1.0 Survey Procedure and Measures 3](#_Toc164948116)

[1.1 Descriptive Statistics 4](#_Toc164948117)

[1.2 Confirmatory Factor Analyses 5](#_Toc164948118)

[1.2.1 Future Time Perspective 6](#_Toc164948119)

[1.2.2 Conservationist norms 7](#_Toc164948120)

[1.2.3 Productivist norms 8](#_Toc164948121)

[1.3 Study 1: SEM Model 9](#_Toc164948122)

[1.3.1 Full SEM Model Iterations 9](#_Toc164948123)

[1.3.2 Full SEM Model Results 11](#_Toc164948124)

[2. Study 2 Supporting Information 18](#_Toc164948125)

[2.0 Survey Procedure and Methods 18](#_Toc164948126)

[2.1 Study 2 Descriptive Statistics 19](#_Toc164948127)

[2.2 Vignette Experiment Results 20](#_Toc164948128)

[2.2.1 Model Output Predicting Retiring Land 21](#_Toc164948129)

[2.2.2 Model Output Predicting Reduced Tillage 22](#_Toc164948130)

[2.2.3 Model Output Predicting Tile Drainage 23](#_Toc164948131)

[2.2.4 Model Output Predicting Irrigation 24](#_Toc164948132)

[2.3 Vignette Experiment Parameters 25](#_Toc164948133)

[3. Survey Instruments 26](#_Toc164948134)

[3.1 Study 1 Full Survey Instrument 26](#_Toc164948135)

[3.2 Study 2 Full Survey Instrument 3](#_Toc164948136)

[4. Stata Code for All Analyses 13](#_Toc164948137)

[4.1 Study 1 Code 13](#_Toc164948138)

[4.1.1 Code for Computing Variables 13](#_Toc164948139)

[4.1.2 Code for Confirmatory Factor Analyses 15](#_Toc164948140)

[4.1.3 Code for Structural Equation Model Predicting In-Field Adaptation 16](#_Toc164948141)

[4.2 Study 2 Code 18](#_Toc164948142)

[4.2.1 Code for Computing Variables 18](#_Toc164948143)

[4.2.2 Code for Hierarchical Mixed-effects Logistic Regression Models 20](#_Toc164948144)

[5. Supporting Information References 22](#_Toc164948145)

# 1. Study 1 Supporting Information

## 1.0 Survey Procedure and Measures

Prospective participants were contacted through a mailing sample obtained from Farm Market ID. Farmers were sent an invitation letter, followed by a paper-and-pencil survey three days after. A return envelope and token incentive of a $2 bill were included with the survey. If no response was received, reminder post cards were also sent two and three weeks afterwards.

To measure cover crops, participants were asked if they had planted any cover crops (not including hay or winter wheat) during the past harvest, and on what proportion of their land they had planted cover crops. Because cover crop adoption is low in the study area (Wallander et al., 2021), missing data was coded as zero. To measure tillage, farm operators indicated the proportion of cropland on which they typically use conventional tillage, conservation tillage, rotational no-till, and continuous no-till. Participants were excluded if the indicated proportions exceeded 100% of cropland, and missing data was assumed to be zero. Next, the proportion of land in each type of non-conventional tillage was weighted by their estimated improvement in nutrient runoff reduction, relative to conventional tillage. These weights were then summed into an overall tillage score. To measure conservation crop rotation, farmers indicated their typical crop rotation over a five-year period. If any small grains or conservation crops were indicated in a given year (such as alfalfa, clover, or wheat), the presence of conservation crop was coded as “1”. The proportion of years with cover crops was then calculated for each participant, then weighted by estimated reduction in nitrogen runoff.

To measure future time perspective, a shortened version of the Future subscale of the Zimbardo Time Perspective Inventory (ZPTI) was used (Zimbardo & Boyd, 1999) in order to reduce participant burden. Items were chosen based on the seven highest factor loadings from Zimbardo & Boyd (1999). One item was excluded from this consideration (“I am able to resist temptations when I know that there is work to be done”), as it was determined that the wording of this item could be viewed as uncomfortable for respondents. Participants were asked to rate how characteristic each statement was of their approach to decision-making. Ratings were made on a five-point scale that ranged from “very uncharacteristic” to “very characteristic”. Items pertained to one’s tendency to plan their days, pursue future goals, and meet deadlines (*α* = .688).

To measure conservationist and productivist norms, the degree to which an operator believes that a “good” farmer tends to prioritize conservation and production was measured using the conservationist and productivist subscales of the Farmer Identity Scale (Arbuckle, 2013; McGuire et al., 2015). Although the present studies were primarily interested in conservationist norms, we controlled for productivist norms as the two constructs tend to correlate. The nine-item conservationist subscale (*α* = .845) includes items such as, “A good farmer is one who puts long-term conservation of farm resources before short-term profits”, whereas the eight-item productivist subscale (*α* =.827) includes items like, “A good farmer is one who has the highest yields per acre”. These scales have been shown in previous work to have adequate internal structure (Arbuckle, 2013; McGuire et al., 2015), and to correlate with agricultural conservation intentions and behavior, such as water conservation (Valizadeh et al., 2020), conservation tillage (Lavoie & Wardropper, 2021), and cover crops (Burnett et al., 2018). For each item, participants indicated how important they believed each feature to be, from “Not at all important” (0) to “Very important”.

## Descriptive Statistics

*Supplemental Table 1: Study 1 Descriptive Statistics of all Predictors*

|  | *M* | *SD* | Scale Range | Sample  Range | | *n* |
| --- | --- | --- | --- | --- | --- | --- |
|  |  |  |  | UL | LL |  |
| In-Field Adaptation Index | 0.540 | 0.292 | 0-1.65 | 0 | 1.65 | 570 |
| Future Time Perspective | 0.982 | 0.488 | -2 (“very characteristic”) – 2 (“very uncharacteristic”) | -1.500 | 2 | 555 |
| Cropland Size (acres) | Median = 600, M = 1045.222 | 1628.282 | - | 0 | 21000 | 609 |
| Education | 3.313 | 1.393 | 1 (some high school) – 6 (graduate or professional degree) | 1 | 6 | 587 |
| Livestock | No livestock: 405  Has livestock: 186 | | | | | 591 |
| Conservationist Norm | 3.112 | 0.520 | 0 (“not important at all”) – 4 (“very important”) | 1.444 | 4 | 572 |
| Productivist Norm | 2.333 | 0.715 | 0 (“not important at all”) – 4 (“very important”) | 0 | 4 | 573 |
| Rents land | Does not rent from others: 189  Rents from others: 404 | | | | | 593 |
| Age | 59.790 | 12.963 | - | 22 | 94 | 581 |

## 1.2 Confirmatory Factor Analyses

For each of the confirmatory factor analyses below, first a structural equation model was fit, which included no error covariances. To improve fit, modification indices of covaried measurement errors were examined. In each iteration, the largest modification index was allowed only if doing so was theoretically justifiable. This process was repeated until conventions of acceptable fit were achieved. See the tables below for an outline of this process. Standardized solutions were fit for each model.

### 1.2.1 Future Time Perspective

*Supplemental Table 2a: Fit Statistics of Single-Factor Future Time Perspective Model*

|  | Chi-square model versus saturated | RMSEA | CFI | TLI | SRMR | AIC | BIC | Suggested Modification | Justification |
| --- | --- | --- | --- | --- | --- | --- | --- | --- | --- |
| Model 1 | 164.273,  *p* < .001 | 0.099 | 0.796 | 0.727 | 0.064 | 11449.174 | 11564.131 | Removal of “I take each day as it is” | Little variance in item explained by model |
| Model 2 | 90.125,  *p* < .001 | 0.082 | 0.877 | 0.828 | 0.049 | 10021.719 | 10123.995 | Covary “day should be planned” and “I make lists” | Lists are a means of planning |
| Model 3 | 68.700,  *p* < .001 | 0.071 | 0.913 | 0.872 | 0.042 | 10002.294 | 10108.831 | Covary “It upsets me to be late”, “meet obligations on time” | Both relate to punctuality |
| Model 4 | 51.838,  *p* < .001 | 0.060 | 0.941 | 0.908 | 0.037 | 9987.432 | 10098.231 | - | - |
| Accept-able Fit | (often failed with large samples) | < 0.08 | >0.90 | >0.90 | < 0.10 | (smaller is better) | | - | - |
| Ideal Fit | Ideally, n.s. | < 0.06 | > 0.95 | > 0.95 | < 0.08 |  |  | - | - |

*Note*: Model fit statistics for the single-factor model of future time perspective. Acceptable and ideal fit metrics are taken from Schreiber et al. (2006, Table 2) and West et al. (2012).

### 1.2.2 Conservationist norms

*Supplemental Table 2b: Fit Statistics of Single-Factor Conservationist Norms Scale*

|  | Chi-square model versus saturated | RMSEA | CFI | TLI | SRMR | AIC | BIC | Suggested Modification | Justification |
| --- | --- | --- | --- | --- | --- | --- | --- | --- | --- |
| Model 1 | 169.153,  *p* < .001 | 0.097 | 0.919 | 0.892 | 0.041 | 9929.869 | 10046.481 | Covary “social ecological health of their watershed” and “minimizes soil erosion” | Soil erosion implicates runoff into watersheds |
| Model 2 | 71.256, *p* < .001 | 0.056 | 0.974 | 0.964 | 0.028 | 9833.972 | 9954.903 | - | - |
| Accept-able Fit | (often failed with large samples) | < 0.08 | >0.90 | >0.90 | < 0.10 | (smaller is better) | | - | - |
| Ideal Fit | Ideally, n.s. | < 0.06 | > 0.95 | > 0.95 | < 0.08 |  |  | - | - |

*Note*: Model fit statistics for the single-factor model of Conservationist norms scale. Acceptable and ideal fit metrics are taken from Schreiber et al. (2006, Table 2) and West et al. (2012).

### 1.2.3 Productivist norms

*Supplemental Table 2c: Fit Statistics of Single-Factor Productivist Norms Scale*

|  | Chi-square model versus saturated | RMSEA | CFI | TLI | SRMR | AIC | BIC | Suggested Modification | Justification |
| --- | --- | --- | --- | --- | --- | --- | --- | --- | --- |
| Model 1 | 242.852,  *p* < .001 | 0.142 | 0.842 | 0.779 | 0.063 | 11747.808 | 11851.334 | Covary “highest profit” and “highest yield” per acre | Higher yield begets higher profit |
| Model 2 | 157.982,  *p* < .001 | 0.115 | 0.901 | 0.855 | 0.053 | 11664.938 | 11772.777 | Covary “keeps fields clean” and “fencerows clear of brush” | Cleaning up fields for aesthetic appeal |
| Model 3 | 98.141,  *p* < .001 | 0.090 | 0.943 | 0.912 | 0.043 | 11607.097 | 11719.249 | - | - |
| Accept-able Fit | (often failed with large samples) | < 0.08 | >0.90 | >0.90 | < 0.10 | (smaller is better) | | - | - |
| Ideal Fit | Ideally, n.s. | < 0.06 | > 0.95 | > 0.95 | < 0.08 |  |  | - | - |

*Note*: Model fit statistics for the single-factor model of productivist norms scale. Acceptable and ideal fit metrics are taken from Schreiber et al. (2006, Table 2) and West et al. (2012).

## 1.3 Study 1: SEM Model

### 1.3.1 Full SEM Model Iterations

After the completion of the above confirmatory factor analyses, their structure was incorporated into a partially-latent structural equation model predicting the index of in-field adaptation practices. To improve fit, modification indices of measurement errors, and paths from exogenous measured variables, were examined. In each iteration, the largest modification index was allowed only if doing so was theoretically justifiable. This process was repeated until no further modification indices were suggested by the model. Standardized solutions were fit for each model.

*Supplemental Table 3: Fit Statistics of Mediation Model Predicting In-Field Adaptation Practices*

|  | Chi-square model v saturated | RMSEA | CFI | TLI | SRMR | AIC | BIC | Suggested Modification | Justification |
| --- | --- | --- | --- | --- | --- | --- | --- | --- | --- |
| Model 1 | 957.802,  *p* < .001 | 0.052 | 0.858 | 0.842 | 0.063 | 32301.910 | 32717.615 | Covary “manages profitability and environment”, “highest profit” | Both relate to prioritizing profit |
| Model 2 | 932.686,  *p* < .001 | 0.051 | 0.864 | 0.849 | 0.063 | 32278.795 | 32698.616 | Path from “fencrows clear” to “considers health of streams” (ignored);  path from corn/soy yield to Productivist norms | Producitivst identity includes maxing yield |
| Model 3 | 910.154,  *p* < .001 | 0.050 | 0.870 | 0.855 | 0.059 | 32258.262 | 32682.199 | Covary “keeps fields claen” and “up-to-date equipment” | Appearance concerns; up-to-date equipment can remove organic matter. |
| Model 5 | 889.074,  *p* < .001 | 0.049 | 0.876 | 0.861 | 0.058 | 32239.182 | 32667.235 | Covary “keeps fencerows clear”, considers health of streams” (ignored);  “latest seed and chemical technology”, “maintains soil organic matter” | Seed technology is a plausible means of increasing organic matter. |
| Model 6 | 874.479,  *p* < .001 | 0.048 | 0.879 | 0.864 | 0.058 | 32226.587 | 32658.756 | Covary “minimizes soil erosion” and “minimizes tillage” | Minimizing tillage is a mins of minimizing erosion |
| Model 7 | 860.237,  *p* < .001 | 0.047 | 0.883 | 0.868 | 0.058 | 32214.346 | 32650.630 | - | - |
| Accept-able Fit | (often failed with large samples) | < 0.08 | >0.90 | >0.90 | < 0.10 | (smaller is better) | | - | - |
| Ideal Fit | Ideally, n.s. | < 0.06 | > 0.95 | > 0.95 | < 0.08 |  |  | - | - |

*Note*: Model fit statistics for the mediation model predicting the in-field adaptation practices index. Acceptable and ideal fit metrics are taken from Schreiber et al. (2006, Table 2) and West et al. (2012).

## 1.3.2 Full SEM Model Results

All paths, factor loadings, and model-estimated variances and covariances are shown in the tables below. Estimates from the standardized solution are presented.

*Supplemental Table 4a: Path Coefficients of Mediation Model Predicting In-Field Adaptation*

|  | *β*  (standardized) | *SE* | *z* | *p* | 95% C.I. | |
| --- | --- | --- | --- | --- | --- | --- |
|  |  |  |  |  | *LL* | *UL* |
|  |  |  |  |  |  |  |
| Paths to:  In-Field Adaptation |  |  |  |  |  |  |
| Future | -0.048 | 0.050 | -0.950 | 0.343 | -0.147 | 0.051 |
| ConservationistNorms | 0.409 | 0.050 | 8.120 | < .001 | 0.311 | 0.508 |
| ProductivistNorms | -0.303 | 0.049 | -6.150 | < .001 | -0.400 | -0.207 |
| cropland | 0.072 | 0.040 | 1.790 | 0.073 | -0.007 | 0.151 |
| education | 0.091 | 0.040 | 2.290 | 0.022 | 0.013 | 0.170 |
| livestock | 0.087 | 0.039 | 2.220 | 0.027 | 0.010 | 0.164 |
| yield | 0.011 | 0.041 | 0.270 | 0.785 | -0.069 | 0.092 |
| rents | 0.061 | 0.040 | 1.520 | 0.129 | -0.018 | 0.139 |
| age | -0.008 | 0.041 | -0.210 | 0.836 | -0.088 | 0.071 |
|  |  |  |  |  |  |  |
| intercept | 1.714 | 0.107 | 16.050 | < .001 | 1.504 | 1.923 |
|  |  |  |  |  |  |  |
| Paths to:  ConservationistNorms |  |  |  |  |  |  |
| Future | 0.194 | 0.058 | 3.360 | 0.001 | 0.081 | 0.307 |
|  |  |  |  |  |  |  |
| Paths to:  ProductivistNorms |  |  |  |  |  |  |
| yield | 0.229 | 0.047 | 4.900 | < .001 | 0.137 | 0.321 |
| age | 0.085 | 0.048 | 1.780 | 0.075 | -0.009 | 0.178 |
|  |  |  |  |  |  |  |

*Note*: Total variance explained by model, *R*^2^ = 0.734

*Supplemental Table 4b: Mediation Results*

|  | *β* | *SE* | *z* | *p* | 95% C.I. | |
| --- | --- | --- | --- | --- | --- | --- |
|  |  |  |  |  | *LL* | *UL* |
|  |  |  |  |  |  |  |
| Delta Method | 0.079 | 0.026 | 3.072 | .002 | 0.029 | 0.130 |
| Sobel Test | 0.079 | 0.026 | 3.101 | .002 | 0.029 | 0.130 |
| Monte Carlo* | 0.078 | 0.026 | 3.022 | .003 | 0.031 | 0.135 |
|  |  |  |  |  |  |  |

*Note*: * Monte Carlo-estimated test of the indirect effect (Jose, 2013).

*Supplemental Table 5a:* *Factor Loadings and Marginal Means of Mediation Model Predicting In-Field Adaptation: Future Time Perspective*

|  | Estimate | *SE* | *z* | *p* | 95% C.I. | |
| --- | --- | --- | --- | --- | --- | --- |
|  |  |  |  |  | *LL* | *UL* |
|  |  |  |  |  |  |  |
| *Future Time Perspective* |  |  |  |  |  |  |
| future1 |  |  |  |  |  |  |
| Factor Loading | 0.431 | 0.050 | 8.600 | < .001 | 0.332 | 0.529 |
| *M* | 0.779 | 0.053 | 14.570 | < .001 | 0.674 | 0.884 |
|  |  |  |  |  |  |  |
| future2 |  |  |  |  |  |  |
| Factor Loading | 0.494 | 0.047 | 10.410 | < .001 | 0.401 | 0.587 |
| *M* | 1.332 | 0.064 | 20.700 | < .001 | 1.206 | 1.458 |
|  |  |  |  |  |  |  |
| future3 |  |  |  |  |  |  |
| Factor Loading | 0.417 | 0.050 | 8.370 | < .001 | 0.319 | 0.515 |
| *M* | 1.337 | 0.065 | 20.720 | < .001 | 1.211 | 1.463 |
|  |  |  |  |  |  |  |
| future4 |  |  |  |  |  |  |
| Factor Loading | 0.402 | 0.052 | 7.680 | < .001 | 0.299 | 0.504 |
| *M* | 1.151 | 0.060 | 19.040 | < .001 | 1.032 | 1.269 |
|  |  |  |  |  |  |  |
| future5 |  |  |  |  |  |  |
| Factor Loading | 0.584 | 0.045 | 12.970 | < .001 | 0.496 | 0.672 |
| *M* | 1.851 | 0.077 | 23.990 | < .001 | 1.700 | 2.002 |
|  |  |  |  |  |  |  |
| future6 |  |  |  |  |  |  |
| Factor Loading | 0.621 | 0.043 | 14.490 | < .001 | 0.537 | 0.705 |
| *M* | 1.410 | 0.066 | 21.340 | < .001 | 1.280 | 1.539 |
|  |  |  |  |  |  |  |
| future7 |  |  |  |  |  |  |
| Factor Loading | 0.397 | 0.052 | 7.680 | < .001 | 0.296 | 0.498 |
| *M* | 0.698 | 0.052 | 13.370 | < .001 | 0.596 | 0.801 |
|  |  |  |  |  |  |  |
| future8 |  |  |  |  |  |  |
| Factor Loading | 0.393 | 0.050 | 7.800 | < .001 | 0.294 | 0.491 |
| *M* | 1.033 | 0.058 | 17.800 | < .001 | 0.919 | 1.147 |
|  |  |  |  |  |  |  |

*Note*: Standardized factor loadings and model-estimated means are presented.

*Supplemental Table 5b:* *Factor Loadings and Marginal Means of Mediation Model Predicting In-Field Adaptation: Conservationist norms*

|  | Estimate | *SE* | *z* | *p* | 95% C.I. | |
| --- | --- | --- | --- | --- | --- | --- |
|  |  |  |  |  | *LL* | *UL* |
|  |  |  |  |  |  |  |
| *Conservationist norms* |  |  |  |  |  |  |
| cons1 |  |  |  |  |  |  |
| Factor Loading | 0.510 | 0.039 | 13.220 | < .001 | 0.434 | 0.585 |
| *M* | 5.071 | 0.174 | 29.110 | < .001 | 4.729 | 5.412 |
|  |  |  |  |  |  |  |
| cons2 |  |  |  |  |  |  |
| Factor Loading | 0.593 | 0.035 | 17.130 | < .001 | 0.525 | 0.661 |
| *M* | 3.609 | 0.128 | 28.170 | < .001 | 3.358 | 3.860 |
|  |  |  |  |  |  |  |
| cons3 |  |  |  |  |  |  |
| Factor Loading | 0.601 | 0.034 | 17.590 | < .001 | 0.534 | 0.668 |
| *M* | 4.513 | 0.155 | 29.200 | < .001 | 4.210 | 4.816 |
|  |  |  |  |  |  |  |
| cons4 |  |  |  |  |  |  |
| Factor Loading | 0.740 | 0.026 | 28.200 | < .001 | 0.688 | 0.791 |
| *M* | 3.808 | 0.134 | 28.440 | < .001 | 3.545 | 4.070 |
|  |  |  |  |  |  |  |
| cons5 |  |  |  |  |  |  |
| Factor Loading | 0.721 | 0.028 | 26.120 | < .001 | 0.667 | 0.775 |
| *M* | 4.395 | 0.152 | 28.880 | < .001 | 4.097 | 4.693 |
|  |  |  |  |  |  |  |
| cons6 |  |  |  |  |  |  |
| Factor Loading | 0.668 | 0.031 | 21.700 | < .001 | 0.608 | 0.729 |
| *M* | 5.320 | 0.182 | 29.300 | < .001 | 4.964 | 5.676 |
|  |  |  |  |  |  |  |
| cons7 |  |  |  |  |  |  |
| Factor Loading | 0.621 | 0.033 | 19.040 | < .001 | 0.557 | 0.685 |
| *M* | 3.925 | 0.137 | 28.710 | < .001 | 3.657 | 4.193 |
|  |  |  |  |  |  |  |
| cons8 |  |  |  |  |  |  |
| Factor Loading | 0.697 | 0.029 | 24.290 | < .001 | 0.641 | 0.754 |
| *M* | 4.064 | 0.142 | 28.630 | < .001 | 3.786 | 4.343 |
|  |  |  |  |  |  |  |
| cons9 |  |  |  |  |  |  |
| Factor Loading | 0.435 | 0.042 | 10.410 | < .001 | 0.353 | 0.517 |
| *M* | 2.555 | 0.096 | 26.580 | < .001 | 2.367 | 2.744 |
|  |  |  |  |  |  |  |

*Note*: Standardized factor loadings and model-estimated means are presented.

*Supplemental Table 5c:* *Factor Loadings and Marginal Means of Mediation Model Predicting In-Field Adaptation: Productivist norms*

|  | Estimate | *SE* | *z* | *p* | 95% C.I. | |
| --- | --- | --- | --- | --- | --- | --- |
|  |  |  |  |  | *LL* | *UL* |
|  |  |  |  |  |  |  |
| *Productivist norms* |  |  |  |  |  |  |
| prod1 |  |  |  |  |  |  |
| Factor Loading | 0.623 | 0.034 | 18.500 | < .001 | 0.557 | 0.690 |
| *M* | 2.974 | 0.107 | 27.740 | < .001 | 2.764 | 3.184 |
|  |  |  |  |  |  |  |
| prod2 |  |  |  |  |  |  |
| Factor Loading | 0.580 | 0.037 | 15.830 | < .001 | 0.508 | 0.652 |
| *M* | 1.201 | 0.061 | 19.690 | < .001 | 1.082 | 1.321 |
|  |  |  |  |  |  |  |
| prod3 |  |  |  |  |  |  |
| Factor Loading | 0.564 | 0.037 | 15.250 | < .001 | 0.492 | 0.637 |
| *M* | 2.337 | 0.089 | 26.260 | < .001 | 2.163 | 2.512 |
|  |  |  |  |  |  |  |
| prod4 |  |  |  |  |  |  |
| Factor Loading | 0.634 | 0.037 | 17.340 | < .001 | 0.562 | 0.706 |
| *M* | 3.691 | 0.129 | 28.550 | < .001 | 3.437 | 3.944 |
|  |  |  |  |  |  |  |
| prod5 |  |  |  |  |  |  |
| Factor Loading | 0.568 | 0.038 | 14.970 | < .001 | 0.494 | 0.642 |
| *M* | 2.388 | 0.091 | 26.140 | < .001 | 2.209 | 2.567 |
|  |  |  |  |  |  |  |
| prod6 |  |  |  |  |  |  |
| Factor Loading | 0.697 | 0.030 | 23.060 | < .001 | 0.638 | 0.757 |
| *M* | 2.037 | 0.081 | 25.070 | < .001 | 1.878 | 2.196 |
|  |  |  |  |  |  |  |
| prod7 |  |  |  |  |  |  |
| Factor Loading | 0.667 | 0.033 | 20.280 | < .001 | 0.603 | 0.732 |
| *M* | 1.285 | 0.063 | 20.520 | < .001 | 1.162 | 1.408 |
|  |  |  |  |  |  |  |
| prod8 |  |  |  |  |  |  |
| Factor Loading | 0.569 | 0.037 | 15.480 | < .001 | 0.497 | 0.641 |
| *M* | 2.694 | 0.100 | 26.900 | < .001 | 2.498 | 2.891 |
|  |  |  |  |  |  |  |

*Note*: Standardized factor loadings and model-estimated means are presented.

*Supplemental Table 6:* *Error Variances of Endogenous Variables in Mediation Model Predicting In-Field Adaptation*

|  | σ^2^ | *SE* | 95% C.I. | |
| --- | --- | --- | --- | --- |
|  |  |  | *LL* | *UL* |
| future1 | 0.815 | 0.043 | 0.734 | 0.904 |
| future2 | 0.756 | 0.047 | 0.670 | 0.854 |
| future3 | 0.826 | 0.042 | 0.749 | 0.912 |
| future4 | 0.839 | 0.042 | 0.760 | 0.925 |
| future5 | 0.659 | 0.053 | 0.564 | 0.771 |
| future6 | 0.614 | 0.053 | 0.518 | 0.728 |
| future7 | 0.842 | 0.041 | 0.766 | 0.927 |
| future8 | 0.846 | 0.040 | 0.772 | 0.927 |
| cons1 | 0.740 | 0.039 | 0.667 | 0.821 |
| cons2 | 0.649 | 0.041 | 0.573 | 0.734 |
| cons3 | 0.638 | 0.041 | 0.563 | 0.724 |
| cons4 | 0.453 | 0.039 | 0.383 | 0.535 |
| cons5 | 0.481 | 0.040 | 0.409 | 0.565 |
| cons6 | 0.553 | 0.041 | 0.478 | 0.640 |
| cons7 | 0.615 | 0.040 | 0.540 | 0.699 |
| cons8 | 0.514 | 0.040 | 0.441 | 0.598 |
| cons9 | 0.811 | 0.036 | 0.743 | 0.885 |
| prod1 | 0.611 | 0.042 | 0.534 | 0.699 |
| prod2 | 0.663 | 0.043 | 0.585 | 0.752 |
| prod3 | 0.681 | 0.042 | 0.604 | 0.768 |
| prod4 | 0.598 | 0.046 | 0.513 | 0.696 |
| prod5 | 0.677 | 0.043 | 0.598 | 0.767 |
| prod6 | 0.514 | 0.042 | 0.437 | 0.603 |
| prod7 | 0.555 | 0.044 | 0.475 | 0.648 |
| prod8 | 0.676 | 0.042 | 0.599 | 0.763 |
| adaptation_index | 0.808 | 0.036 | 0.741 | 0.881 |
| ConservationistNorms | 0.962 | 0.022 | 0.919 | 1.007 |
| ProductivistNorms | 0.941 | 0.023 | 0.898 | 0.987 |
| Future | 1.000 | . | . | . |
|  |  |  |  |  |

*Note*: The error variances of all endogenous variables are presented here. Item labels are as follows: “future” corresponds to items of the Zimbardo Time Perspective Inventory (Zimbardo & Boyd, 1999); “cons” corresponds to the conservationist norms subscale of the Farmer Identity Scale, and “prod” the productivist norms subscale of the same (Arbuckle, 2013; McGuire et al., 2015).

*Supplemental Table 7a:* *Model-Estimated Error Covariances in Mediation Model Predicting In-Field Adaptation*

|  | cov(e.x,e.y) | *SE* | *z* | *p* | 95% C.I. | |
| --- | --- | --- | --- | --- | --- | --- |
|  |  |  |  |  | *LL* | *UL* |
|  |  |  |  |  |  |  |
| e.future1, e.future7 | 0.154 | 0.051 | 3.030 | 0.002 | 0.055 | 0.254 |
| e.future4, e.future5 | 0.209 | 0.054 | 3.900 | < .001 | 0.104 | 0.314 |
| e.cons3, e.prod1 | 0.197 | 0.050 | 3.930 | < .001 | 0.099 | 0.296 |
| e.cons5, e.cons6 | 0.492 | 0.040 | 12.290 | < .001 | 0.414 | 0.571 |
| e.cons6, e.cons9 | 0.143 | 0.037 | 3.820 | < .001 | 0.070 | 0.216 |
| e.cons7, e.prod3 | 0.215 | 0.043 | 5.030 | < .001 | 0.131 | 0.298 |
| e.cons9, e.adaptation_index | 0.437 | 0.040 | 10.870 | < .001 | 0.359 | 0.516 |
| e.prod3, e.prod6 | 0.458 | 0.042 | 10.940 | < .001 | 0.376 | 0.540 |
| e.prod4, e.prod5 | 0.260 | 0.054 | 4.840 | < .001 | 0.155 | 0.365 |
| e.prod4, e.prod7 | -0.257 | 0.059 | -4.380 | < .001 | -0.372 | -0.142 |
| e.ConservationistNorms, e.ProductivistNorms | 0.392 | 0.051 | 7.730 | < .001 | 0.292 | 0.491 |
|  |  |  |  |  |  |  |

*Note*: The model-estimated error covariances are presented here. Item labels are as follows: “future” corresponds to items of the Zimbardo Time Perspective Inventory (Zimbardo & Boyd, 1999); “cons” corresponds to the conservationist norms subscale of the Farmer Identity Scale, and “prod” the productivist norms subscale of the same (Arbuckle, 2013; McGuire et al., 2015).

*Supplemental Table 7b:* *Model-Estimated Exogenous Variable Covariances in Mediation Model Predicting In-Field Adaptation*

|  | cov(x,y) | *SE* | *z* | *p* | 95% C.I. | |
| --- | --- | --- | --- | --- | --- | --- |
|  |  |  |  |  | *LL* | *UL* |
|  |  |  |  |  |  |  |
| cropland, Future | 0.110 | 0.055 | 1.990 | 0.046 | 0.002 | 0.218 |
| education, Future | 0.125 | 0.055 | 2.250 | 0.024 | 0.016 | 0.234 |
| livestock, Future | 0.001 | 0.056 | 0.020 | 0.981 | -0.108 | 0.111 |
| yield, Future | 0.054 | 0.055 | 0.970 | 0.334 | -0.055 | 0.162 |
| rents, Future | -0.099 | 0.055 | -1.800 | 0.072 | -0.207 | 0.009 |
| age, Future | 0.034 | 0.055 | 0.620 | 0.534 | -0.074 | 0.143 |
|  |  |  |  |  |  |  |

*Supplemental Table 8: Covariance Residuals Matrix*

|  |  |  |  |  |  |  |  |  |  |  |  |  |  |  |  |  |  |  |  |  |  |  |  |  |  |  |  |  |  |  |  |  |
| --- | --- | --- | --- | --- | --- | --- | --- | --- | --- | --- | --- | --- | --- | --- | --- | --- | --- | --- | --- | --- | --- | --- | --- | --- | --- | --- | --- | --- | --- | --- | --- | --- |
|  | future1 | future2 | future3 | future4 | future5 | future6 | future7 | future8 | Cons1 | Cons2 | Cons3 | Cons4 | Cons5 | Cons6 | Cons7 | Cons8 | Cons9 | Prod1 | Prod2 | Prod3 | Prod4 | Prod5 | Prod6 | Prod7 | Prod8 | Adapta-  tion Index | Crop-land | Edu-cation | Live-stock | Yield | Rents | Age |
|  | | | | | | | | | | | | | | | | |  |  |  |  |  |  |  |  |  |  |  |  |  |  |  |  |
| future1 | 0 |  |  |  |  |  |  |  |  |  |  |  |  |  |  |  |  |  |  |  |  |  |  |  |  |  |  |  |  |  |  |  |
| future2 | 0.066 | 0 |  |  |  |  |  |  |  |  |  |  |  |  |  |  |  |  |  |  |  |  |  |  |  |  |  |  |  |  |  |  |
| future3 | -0.015 | -0.035 | 0 |  |  |  |  |  |  |  |  |  |  |  |  |  |  |  |  |  |  |  |  |  |  |  |  |  |  |  |  |  |
| future4 | 0.034 | -0.026 | 0.042 | 0 |  |  |  |  |  |  |  |  |  |  |  |  |  |  |  |  |  |  |  |  |  |  |  |  |  |  |  |  |
| future5 | -0.013 | -0.028 | 0.035 | 0 | 0 |  |  |  |  |  |  |  |  |  |  |  |  |  |  |  |  |  |  |  |  |  |  |  |  |  |  |  |
| future6 | -0.008 | 0.008 | -0.015 | -0.021 | 0.023 | 0 |  |  |  |  |  |  |  |  |  |  |  |  |  |  |  |  |  |  |  |  |  |  |  |  |  |  |
| future7 | 0 | 0.025 | -0.074 | 0.019 | -0.065 | 0.034 | 0 |  |  |  |  |  |  |  |  |  |  |  |  |  |  |  |  |  |  |  |  |  |  |  |  |  |
| future8 | -0.071 | -0.005 | 0.068 | -0.013 | 0.007 | -0.011 | 0.017 | 0 |  |  |  |  |  |  |  |  |  |  |  |  |  |  |  |  |  |  |  |  |  |  |  |  |
| Cons1 | -0.014 | 0.008 | -0.039 | 0.038 | 0.001 | 0.014 | 0.002 | 0.057 | 0.002 |  |  |  |  |  |  |  |  |  |  |  |  |  |  |  |  |  |  |  |  |  |  |  |
| Cons2 | 0.026 | 0.052 | 0.048 | 0.062 | 0.018 | 0.014 | 0.042 | 0.108 | 0.019 | 0.004 |  |  |  |  |  |  |  |  |  |  |  |  |  |  |  |  |  |  |  |  |  |  |
| Cons3 | -0.029 | 0.047 | 0.059 | 0.012 | 0.01 | 0.013 | -0.027 | 0.047 | 0.011 | 0.049 | 0.009 |  |  |  |  |  |  |  |  |  |  |  |  |  |  |  |  |  |  |  |  |  |
| Cons4 | 0.015 | 0.055 | -0.014 | 0.051 | -0.011 | -0.012 | 0.028 | 0 | 0.002 | -0.002 | 0.02 | 0.006 |  |  |  |  |  |  |  |  |  |  |  |  |  |  |  |  |  |  |  |  |
| Cons5 | -0.048 | -0.015 | -0.014 | 0.051 | -0.042 | -0.033 | -0.007 | 0.033 | 0.016 | -0.017 | -0.014 | 0.015 | 0.005 |  |  |  |  |  |  |  |  |  |  |  |  |  |  |  |  |  |  |  |
| Cons6 | -0.029 | -0.004 | 0.007 | 0.02 | -0.03 | -0.006 | -0.019 | 0.03 | 0.013 | 0.001 | 0.009 | -0.005 | 0.003 | 0.002 |  |  |  |  |  |  |  |  |  |  |  |  |  |  |  |  |  |  |
| Cons7 | 0.029 | 0.072 | 0.062 | 0.078 | 0.017 | -0.002 | 0.065 | 0.063 | -0.024 | 0.012 | -0.019 | 0.028 | 0.007 | -0.019 | 0.005 |  |  |  |  |  |  |  |  |  |  |  |  |  |  |  |  |  |
| Cons8 | 0.002 | 0.019 | -0.002 | 0.048 | -0.027 | 0.04 | 0.03 | 0.032 | 0.006 | -0.015 | -0.013 | 0.007 | 0.029 | 0.022 | 0 | 0.005 |  |  |  |  |  |  |  |  |  |  |  |  |  |  |  |  |
| Cons9 | 0.052 | 0.081 | -0.012 | 0.1 | 0.045 | 0.088 | 0.049 | 0.021 | -0.028 | 0.038 | 0.035 | -0.041 | -0.011 | 0.011 | 0.053 | -0.029 | 0.014 |  |  |  |  |  |  |  |  |  |  |  |  |  |  |  |
| Prod1 | 0.082 | 0.06 | 0.056 | 0.055 | 0.066 | 0.078 | 0.04 | 0.063 | 0.091 | 0.029 | 0.052 | 0.042 | 0.032 | 0.015 | 0.005 | 0.043 | 0.09 | 0.017 |  |  |  |  |  |  |  |  |  |  |  |  |  |  |
| Prod2 | 0.189 | 0.079 | 0.052 | 0.051 | -0.037 | 0.086 | 0.128 | 0.009 | 0.009 | -0.084 | -0.031 | -0.056 | -0.016 | -0.008 | -0.004 | 0.003 | 0.099 | -0.038 | 0.01 |  |  |  |  |  |  |  |  |  |  |  |  |  |
| Prod3 | 0.06 | 0.129 | 0.029 | 0.035 | 0.024 | 0.048 | 0.056 | 0.086 | 0.056 | -0.109 | 0.044 | -0.056 | 0.014 | -0.038 | 0.01 | 0.043 | 0.046 | -0.038 | 0.136 | 0.01 |  |  |  |  |  |  |  |  |  |  |  |  |
| Prod4 | 0.059 | 0.058 | 0.057 | 0.084 | 0.057 | 0.088 | 0.013 | 0.067 | 0.058 | -0.02 | 0.078 | 0.008 | 0.023 | 0.018 | 0.001 | 0.088 | 0.006 | 0.026 | -0.084 | -0.023 | 0.012 |  |  |  |  |  |  |  |  |  |  |  |
| Prod5 | 0.047 | 0.116 | 0.023 | 0.063 | 0.062 | 0.076 | 0.04 | 0.041 | 0.031 | 0.001 | 0.08 | 0.012 | 0.017 | 0.028 | -0.011 | 0.166 | 0.034 | 0.008 | -0.036 | 0.034 | 0.025 | 0.007 |  |  |  |  |  |  |  |  |  |  |
| Prod6 | 0.068 | 0.085 | 0.042 | 0.048 | -0.007 | 0.042 | 0.069 | 0.065 | 0.052 | -0.135 | 0.014 | -0.078 | 0.029 | 0.02 | 0.014 | 0.013 | 0.042 | 0 | 0.149 | 0.014 | -0.003 | 0.005 | 0.011 |  |  |  |  |  |  |  |  |  |
| Prod7 | 0.107 | 0.09 | -0.053 | -0.014 | -0.052 | -0.002 | 0.069 | -0.053 | 0.017 | -0.043 | 0.024 | -0.017 | -0.015 | -0.042 | -0.018 | -0.014 | 0.106 | 0.026 | 0.093 | 0.071 | -0.01 | -0.081 | 0.046 | 0.009 |  |  |  |  |  |  |  |  |
| Prod8 | 0.149 | 0.11 | 0.036 | 0.064 | 0.032 | 0.134 | 0.163 | 0.034 | 0.039 | -0.011 | 0.064 | 0.082 | 0.02 | 0.015 | 0.066 | 0.147 | 0.161 | 0.015 | -0.044 | -0.06 | 0.055 | 0.099 | -0.08 | -0.015 | 0.006 |  |  |  |  |  |  |  |
| In-Field Adaptations Index | -0.01 | -0.004 | -0.008 | 0.009 | 0.007 | 0.019 | -0.004 | -0.009 | -0.023 | 0.018 | 0.014 | -0.011 | 0 | 0.011 | 0.011 | -0.01 | -0.009 | 0.018 | 0.005 | 0.027 | -0.019 | 0.002 | 0.008 | 0.026 | 0.018 | -0.003 |  |  |  |  |  |  |
| Cropland | 0.04 | 0.023 | 0.017 | -0.084 | 0.019 | -0.043 | 0.027 | 0.018 | -0.005 | 0.023 | 0.004 | 0.021 | 0.007 | -0.01 | 0.04 | 0.009 | -0.036 | 0.023 | 0.1 | 0.134 | -0.011 | 0.029 | 0.074 | 0.162 | 0.064 | -0.01 | 0 |  |  |  |  |  |
| Education | 0.046 | -0.002 | -0.031 | -0.012 | -0.015 | -0.043 | 0.203 | -0.004 | -0.049 | 0.054 | -0.015 | -0.001 | 0.006 | -0.009 | 0.07 | -0.071 | 0.015 | 0 | -0.058 | 0.037 | -0.117 | -0.109 | -0.058 | -0.099 | -0.044 | 0.009 | 0 | 0 |  |  |  |  |
| Livestock | 0.017 | 0.015 | 0.041 | -0.05 | -0.023 | 0.004 | -0.028 | -0.012 | -0.015 | 0.018 | 0.019 | 0.015 | 0.023 | 0.028 | 0.015 | 0.017 | -0.026 | -0.053 | 0.005 | -0.023 | -0.044 | -0.012 | -0.037 | -0.011 | 0.041 | 0 | 0 | 0 | 0 |  |  |  |
| Yield | -0.01 | -0.046 | -0.015 | 0.046 | 0.035 | 0.01 | 0.018 | -0.075 | 0.031 | 0.024 | 0.075 | 0.046 | 0.076 | 0.036 | 0.01 | 0.13 | -0.041 | 0.075 | -0.111 | -0.029 | 0.048 | 0.13 | 0.032 | -0.019 | 0.049 | -0.002 | 0 | 0 | 0 | 0 |  |  |
| Rents | -0.017 | 0.002 | 0.005 | -0.022 | -0.003 | 0.002 | 0.017 | 0.009 | -0.007 | 0.008 | 0 | 0.003 | 0.019 | -0.005 | 0.023 | -0.005 | -0.04 | 0.003 | -0.018 | 0.04 | 0.009 | -0.019 | 0.011 | -0.004 | -0.017 | -0.004 | 0 | 0 | 0 | 0 | 0 |  |
| Age | 0.013 | 0.007 | -0.06 | 0.017 | 0.012 | 0.006 | -0.001 | -0.02 | 0.018 | 0.008 | 0.005 | 0.01 | 0.019 | 0.018 | -0.035 | 0.026 | 0.161 | 0.093 | 0.039 | -0.097 | -0.062 | 0.031 | -0.076 | 0.044 | 0.052 | 0.021 | 0 | 0 | 0 | 0 | 0 | 0 |
|  | | | | | | | | | | | | | | | | |  |  |  |  |  |  |  |  |  |  |  |  |  |  |  |  |

*Note*: The full model-estimated covariance residual matrix is presented here. Item labels are as follows: “future” corresponds to items of the Zimbardo Time Perspective Inventory (Zimbardo & Boyd, 1999); “cons” corresponds to the conservationist norms subscale of the Farmer Identity Scale, and “prod” the productivist norms subscale of the same (Arbuckle, 2013; McGuire et al., 2015).

# 2. Study 2 Supporting Information

## 2.0 Survey Procedure and Methods

*Supplemental Figure 1: Eastern Corn Belt Region and Selected Watersheds*


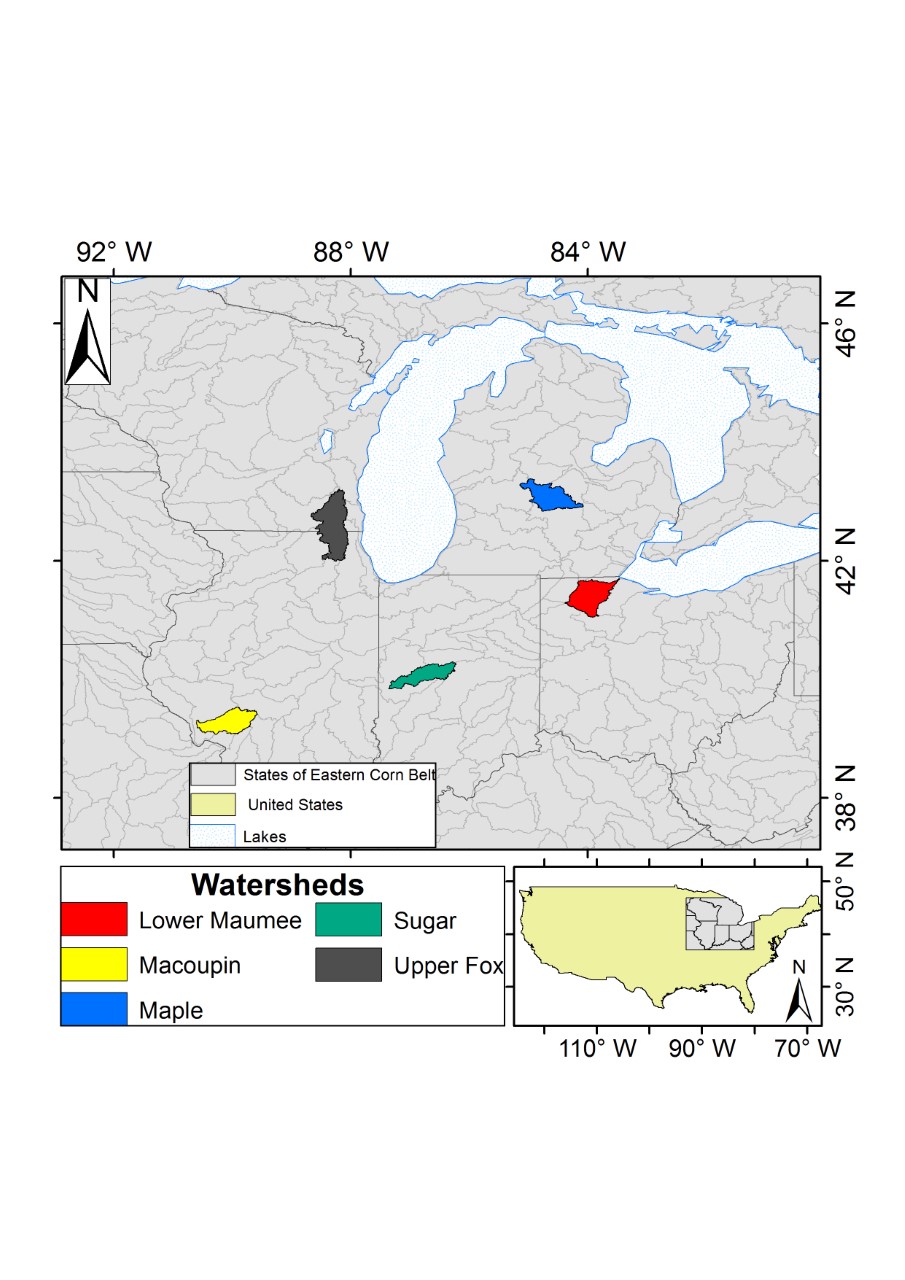


## 2.1 Study 2 Descriptive Statistics

*Supplemental Table 9: Study 2 Descriptive Statistics*

|  | *M* | *SD* | Scale Range | Sample  Range | | *n* |
| --- | --- | --- | --- | --- | --- | --- |
|  |  |  |  | LL | UL |  |
| Concern for Future Weather Pattern Change on Local Agriculture | 1.762 | 0.938 | 0 (“not concerned at all”) –  4(“very concerned”) | 0.00 | 4.00 | 871 |
| Cropland Size (acres) | 879.011 | 1213.422 | - | 15.00 | 10600.00 | 752 |
| Education | 3.349 | 1.394 | 1 (some high school) –  6 (graduate or professional degree) | 1.00 | 6.00 | 897 |
| Livestock | No livestock: 674  Has livestock: 218 | | | | | 892 |
| Conservationist Identity | 2.945 | 0.639 | 0 (“not important at all”) –  4 (“very important”) | 0.00 | 4.00 | 871 |
| Productivist Identity | 2.120 | 0.743 | 0 (“not important at all”) –  4 (“very important”) | 0.00 | 4.00 | 871 |
| Insurance | No crop insurance: 240  Has crop insurance: 642 | | | | | 882 |
| Rents land | Does not rent from others: 300  Rents from others: 593 | | | | | 893 |
| Age | 63.205 | 12.654 | - | 25.00 | 94.00 | 887 |
| Corn Yield (bushels/acre) | 185.196 | 34.136 | - | 0.00 | 280.00 | 800 |
| Soy Yield (bushels/acre) | 58.620 | 12.712 | - | 0.00 | 150.00 | 815 |

### 2.2 Vignette Experiment Results

In the vignette experiment, rainfall levels were described to farmers as either decreasing, staying the same, or increasing, while conservation payment levels either decreased or increased by $100 from their baseline levels in the respondent’s area. Details regarding the levels of each vignette experiment parameter are summarized in table 10.

Because of the repeated-measures design, data were analyzed in long form, such that the vignette scenario observations were nested within participants, and participants were also nested within “group”, or the combination of vignette parameters that were observed. For each outcome variable of interest, a hierarchical mixed-effects logistic regression was estimated.

### 2.2.1 Model Output Predicting Retiring Land

*Supplemental Table 10a: Likelihood of Planning to Enroll More Land in Conservation Retirement Program*

|  | Coefficient (log-odds) | *SE* | *OR (*odds ratio) | *p* | 95% C.I. | |
| --- | --- | --- | --- | --- | --- | --- |
|  |  |  |  |  | *LL* | *UL* |
| Increasing vs. Decreasing conservation payment | 1.987*** | 0.202 | 7.295 | < .001 | 1.591 | 2.383 |
| Less rain vs baseline | 0.817*** | 0.228 | 2.264 | < .001 | 0.370 | 1.264 |
| More rain vs baseline | 0.418 | 0.226 | 1.519 | .064 | -0.024 | 0.860 |
|  |  |  |  |  |  |  |
| Future weather concern | 0.402*** | 0.088 | 1.495 | < .001 | 0.229 | 0.574 |
|  |  |  |  |  |  |  |
| Cropland acres | 0.179 | 0.100 | 1.196 | .074 | -0.018 | 0.376 |
| Formal education | 0.312*** | 0.083 | 1.366 | < .001 | 0.150 | 0.474 |
| Livestock | -0.260 | 0.198 | 0.771 | .190 | -0.649 | 0.129 |
|  |  |  |  |  |  |  |
| Conservationist norms | 0.345*** | 0.097 | 1.412 | < .001 | 0.155 | 0.534 |
| Productivist norms | -0.279** | 0.092 | 0.756 | .002 | -0.460 | -0.099 |
| Yield | -0.154 | 0.106 | 0.857 | .147 | -0.363 | 0.054 |
| Clay soil percent | 0.033 | 0.085 | 1.033 | .702 | -0.134 | 0.199 |
| Rents land | 0.007 | 0.205 | 1.008 | .971 | -0.395 | 0.410 |
| Crop insurance | -0.227 | 0.223 | 0.797 | .310 | -0.664 | 0.211 |
| Age | 0.018 | 0.087 | 1.019 | .834 | -0.153 | 0.190 |
| Baseline conservation payment | 0.404*** | 0.096 | 1.498 | < .001 | 0.217 | 0.592 |
|  |  |  |  |  |  |  |
| Intercept | -3.739*** | 0.415 | 0.024 | < .001 | -4.552 | -2.926 |
|  |  |  |  |  |  |  |
| Random intercept variability | 0.246 | 0.126 | 1.134 |  | 0.090 | 0.671 |
|  |  |  |  |  |  |  |
| Total observations | 1547 |  |  |  |  |  |
|  |  |  |  |  |  |  |

*Note*: *** indicates *p* < .001; ** indicates *p* < .01 , * indicates *p* < .05. In the simplified model that includes only significant control variables as predictors, productivist norms drops below significance (*p* = .065). Area under receiver operating characteristic curve (ROC curve) = 0.829, *SE* = 0.014, 95% C.I. [0.812, 0.856].

### 2.2.2 Model Output Predicting Reduced Tillage

*Supplemental Table 10b: Likelihood of Planning to Increase No-Till/Conservation Tillage*

|  | Coefficient (log-odds) | *SE* | *OR (*odds ratio) | *p* | 95% C.I. | |
| --- | --- | --- | --- | --- | --- | --- |
|  |  |  |  |  | *LL* | *UL* |
| Increasing vs. Decreasing conservation payment | -0.088 | 0.140 | 0.915 | .528 | -0.363 | 0.186 |
| Less rain vs baseline | 0.642*** | 0.174 | 1.901 | < .001 | 0.301 | 0.983 |
| More rain vs baseline | 0.047 | 0.181 | 1.048 | .793 | -0.307 | 0.401 |
|  |  |  |  |  |  |  |
| Future weather concern | 0.395*** | 0.076 | 1.485 | < .001 | 0.247 | 0.544 |
|  |  |  |  |  |  |  |
| Cropland acres | 0.221* | 0.086 | 1.248 | .010 | 0.054 | 0.389 |
| Formal education | 0.026 | 0.071 | 1.026 | .714 | -0.113 | 0.165 |
| Livestock | 0.435** | 0.155 | 1.546 | .005 | 0.132 | 0.739 |
|  |  |  |  |  |  |  |
| Conservationist norms | 0.043 | 0.079 | 1.044 | .584 | -0.111 | 0.198 |
| Productivist norms | -0.164* | 0.079 | 0.849 | .037 | -0.318 | -0.010 |
| Yield | -0.074 | 0.090 | 0.929 | .411 | -0.250 | 0.102 |
| Clay soil percent | 0.087 | 0.071 | 1.091 | .218 | -0.052 | 0.226 |
| Rents land | -0.038 | 0.173 | 0.962 | .825 | -0.378 | 0.301 |
| Crop insurance | 0.248 | 0.194 | 1.281 | .202 | -0.133 | 0.629 |
| Age | -0.013 | 0.074 | 0.987 | .858 | -0.159 | 0.132 |
| Baseline conservation payment | -0.012 | 0.079 | 0.988 | .878 | -0.168 | 0.143 |
|  |  |  |  |  |  |  |
| Intercept | -1.491*** | 0.311 | 0.225 | < .001 | -2.099 | -0.882 |
|  |  |  |  |  |  |  |
| Random intercept variability | 0.052 | 0.054 | 1.055 |  | 0.007 | 0.405 |
|  |  |  |  |  |  |  |
| Total observations | 1547 |  |  |  |  |  |
|  |  |  |  |  |  |  |

*Note*: *** indicates *p* < .001; ** indicates *p* < .01 , * indicates *p* < .05. Area under receiver operating characteristic curve (ROC curve) = 0.694, *SE* = 0.017, 95% C.I. [0.661, 0.726]. Because ROC bordered fair fit, a model that additionally included random intercept by participant ID was estimated. The conclusions from this model remain the same as those presented here, except for livestock, which dropped below significance (*p* = .099) (area under ROC curve = 0.971, *SE* = 0.004, 95% C.I [0.964, 0.978]).

### 2.2.3 Model Output Predicting Tile Drainage

*Supplemental Table 10c: Likelihood of Planning to Install More Tile Drainage*

|  | Coefficient (log-odds) | *SE* | *OR (*odds ratio) | *p* | 95% C.I. | |
| --- | --- | --- | --- | --- | --- | --- |
|  |  |  |  |  | *LL* | *UL* |
| Increasing vs. Decreasing conservation payment | 0.004 | 0.173 | 1.004 | .979 | -0.335 | 0.344 |
| Less rain vs baseline | -1.034*** | 0.293 | 0.355 | < .001 | -1.609 | -0.460 |
| More rain vs baseline | 0.983*** | 0.218 | 2.672 | < .001 | 0.556 | 1.409 |
|  |  |  |  |  |  |  |
| Future weather concern | 0.180* | 0.079 | 1.198 | .022 | 0.026 | 0.335 |
|  |  |  |  |  |  |  |
| Cropland acres | 0.389*** | 0.092 | 1.475 | < .001 | 0.209 | 0.568 |
| Formal education | 0.087 | 0.076 | 1.091 | .249 | -0.061 | 0.236 |
| Livestock | 0.143 | 0.171 | 1.154 | .403 | -0.192 | 0.478 |
|  |  |  |  |  |  |  |
| Conservationist norms | -0.039 | 0.084 | 0.962 | .644 | -0.204 | 0.126 |
| Productivist norms | 0.006 | 0.084 | 1.006 | .946 | -0.159 | 0.170 |
| Yield | -0.129 | 0.096 | 0.879 | .181 | -0.318 | 0.060 |
| Clay soil percent | 0.218** | 0.075 | 1.243 | 0.004 | 0.071 | 0.364 |
| Rents land | -0.211 | 0.183 | 0.810 | .248 | -0.569 | 0.147 |
| Crop insurance | 0.333 | 0.206 | 1.395 | .107 | -0.072 | 0.737 |
| Age | -0.159* | 0.078 | 0.853 | .042 | -0.312 | -0.005 |
| Baseline conservation payment | 0.089 | 0.086 | 1.093 | .299 | -0.079 | 0.257 |
|  |  |  |  |  |  |  |
| Intercept | -3.776*** | 0.410 | 0.023 | < .001 | -4.580 | -2.973 |
|  |  |  |  |  |  |  |
| Random intercept variability | 0.038 | 0.088 | 1.039 |  | < 0.001 | 3.478 |
| Random slope variability: Less rain | 0.614 | 0.446 | 1.847 |  | 0.148 | 2.551 |
| Random slope variability: More rain | 0.412 | 0.253 | 1.510 |  | 0.124 | 1.374 |
|  |  |  |  |  |  |  |
| Total observations | 1547 |  |  |  |  |  |
|  |  |  |  |  |  |  |

*Note*: *** indicates *p* < .001; ** indicates *p* < .01 , * indicates *p* < .05. In the simplified model that includes only significant control variables, age drops below significance (*p* = .051). Area under receiver operating characteristic curve (ROC curve) = 0.813, *SE* = 0.013, 95% C.I. [0.787, 0.838].

### 2.2.4 Model Output Predicting Irrigation

*Supplemental Table 10d: Likelihood of Planning to Invest in Additional Irrigation*

|  | Coefficient (log-odds) | *SE* | *OR (*odds ratio) | *p* | 95% C.I. | |
| --- | --- | --- | --- | --- | --- | --- |
|  |  |  |  |  | *LL* | *UL* |
| Increasing vs. Decreasing conservation payment | -0.183 | 0.279 | 0.833 | 0.512 | -0.730 | 0.364 |
| Less rain vs baseline | 1.488*** | 0.367 | 4.426 | < .001 | 0.768 | 2.207 |
| More rain vs baseline | -0.264 | 0.472 | 0.768 | 0.576 | -1.190 | 0.662 |
|  |  |  |  |  |  |  |
| Future weather concern | -0.040 | 0.144 | 0.961 | 0.781 | -0.322 | 0.242 |
|  |  |  |  |  |  |  |
| Cropland acres | 0.549** | 0.170 | 1.732 | 0.001 | 0.215 | 0.883 |
| Formal education | 0.602*** | 0.142 | 1.826 | < .001 | 0.324 | 0.881 |
| Livestock | 0.467 | 0.295 | 1.595 | 0.114 | -0.112 | 1.046 |
|  |  |  |  |  |  |  |
| Conservationist norms | 0.173 | 0.171 | 1.189 | 0.310 | -0.162 | 0.508 |
| Productivist norms | 0.036 | 0.155 | 1.036 | 0.818 | -0.269 | 0.340 |
| Yield | 0.219 | 0.188 | 1.245 | 0.245 | -0.150 | 0.589 |
| Clay soil percent | -0.217 | 0.160 | 0.805 | 0.175 | -0.532 | 0.097 |
| Rents land | -0.602 | 0.326 | 0.548 | 0.065 | -1.240 | 0.036 |
| Crop insurance | -0.430 | 0.376 | 0.650 | 0.252 | -1.167 | 0.306 |
| Age | 0.100 | 0.151 | 1.105 | 0.508 | -0.195 | 0.395 |
| Baseline conservation payment | -0.516** | 0.164 | 0.597 | 0.002 | -0.837 | -0.194 |
|  |  |  |  |  |  |  |
| Intercept | -3.756*** | 0.643 | 0.023 | < .001 | -5.015 | -2.496 |
|  |  |  |  |  |  |  |
| Random intercept variability | 0.099 | 0.222 | 1.104 |  | 0.001 | 7.998 |
|  |  |  |  |  |  |  |
| Total observations | 1547 |  |  |  |  |  |
|  |  |  |  |  |  |  |

*Note*: *** indicates *p* < .001; ** indicates *p* < .01 , * indicates *p* < .05. Area under receiver operating characteristic curve (ROC curve) = 0.820, *SE* = 0.028, 95% C.I. [0.765, 0.874].

## 2.3 Vignette Experiment Parameters

Also included in the vignette experiment parameters were planting and harvesting dates, as well as the likelihood of the weather conditions occurring over the next ten years. While it is objectively true that it is difficult to predict how climate change will affect weather patterns in any given year, this latter parameter was intended to test how a farmer’s perceived surety of changing weather patterns affects adaptation intentions. However, this parameter exhibited little effect on outcomes, and thus is not reported here. Growing times were included in order to examine effects on crop insurance plans and plans to engage in double-cropping. Because these outcomes and associated parameters are not of interest to the purpose of this manuscript, their results are not reported here. Importantly, all parameters of the vignette experiment were controlled for in all analyses. Supplemental Table 11 below outlines all choice experiment parameters.

*Supplemental Table 11: All Vignette Experiment Parameters*

| Parameter | Description | Coding |
| --- | --- | --- |
|  |  |  |
| Conservation Payment Change | Change in conservation payment from baseline | 0 = $100 less  1 = $100 more |
| Rain More  Rain Less | Change in rain.  Three conditions:  15 inches less,  no change,  10 inches more. Note that referent group is ‘no change’. | *rain_more_*  1= more  0 = no change, early  *rain_less_*  1=less  0= no change, more |
| Scenario | Scenario number (1, 2, or 3); represents linear effect of repeatedly asking. | 1 = 1^st^ scenario  2 = 2^nd^ scenario  3 = 3^rd^ scenario |
|  |  |  |
| Plant Early  Plant Late | Change in planting date. Three conditions:  30 days early,  no change, 45 days late.  Note that referent group is ‘no change’. | *plant_early_*  1 = early  0 = no change, late  *plant_late_*  1=late  0 = no change, early |
| Harvest Late  Harvest Early | Change in harvest date.  Three conditions:  30 days early,  no change,  50 days late.  Note that referent group is ‘no change’. | *harvest_late_*  1= late  0 = no change, early  *harvest_early_*  1=early  0 = no change, late |
| Probability | Probability or surety of changing weather patterns in the future. | 0 = 50% likelihood  1 = 80% likelihood |
|  |  |  |

# 3. Survey Instruments

## 3.1 Study 1 Full Survey Instrument

**Section 1 – Farm Characteristics**

The following questions have to do with your farm operation, including how your land is allocated to different uses, how you manage your land in production, and how rental agreements are arranged. We use this information to understand how the characteristics of a farm influence land use decisions.

1. How large was your total farm operation (owned and rented land) **this past growing season***? For total acres, include cropland, woodland, pasture, wasteland, land in farmsteads, and land in government programs.*

Total farm size: acres


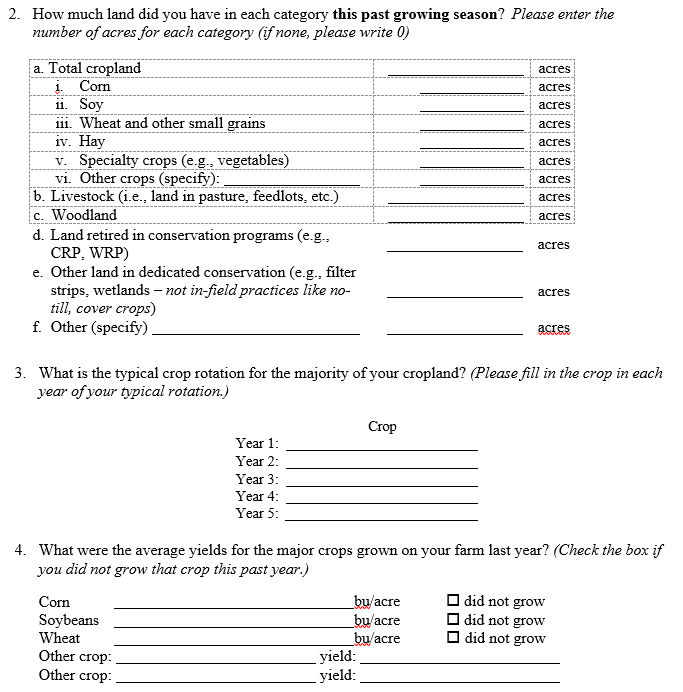


1. Did you plant any cover crops (not including hay or winter wheat) after the 2018 harvest?

 Yes → *please list the crop(s) or mix cover crop(s)*:

 No → *skip to Question 7*

1. On what proportion of your land, approximately, did you plant cover crops after the 2018 harvest?

% of all harvested cropland acres

1. Did you use any filtering or trapping practices (e.g., filter strips, riparian buffers, wetlands) to reduce erosion or capture nutrient runoff from your land in 2018?

 Yes → *please specify*  No → *skip to Question 9*

1. What % of your total harvested cropland acres drain into or across filtering or trapping practice?

% of all harvested cropland acres

1. On approximately what proportion of your cropland do you typically use each type of tillage in your typical rotation, based on the definitions below?

**Conventional**

(inversion tillage and/or system that leaves < 30% residue after planting) %

**Conservation**

(discing/chiseling system that leaves > 30% residue after planting) %

**Rotational No-Till**

(no soil tillage disturbance or full pass tillage in yearly rotation with other

forms of tillage and leaves > 50-60% residue after planting) %

**Continuous No-Till**  % (never tilled, planting directly into crop residue every year)

1. Do you currently lease or use any farmland for alternative energy (e.g., wind, solar) production?

 Yes, wind energy → *Number of turbines:*

 Yes, solar energy → *Number of acres:*

 No

1. Did you receive any income from livestock or poultry raised on your farm in 2018?

 Yes→ How many of each did you raise or manage in 2018? *(Please fill in the number below)*

Dairy cows

Beef cows

Calves, heifers and/or feeders

Swine (one-time capacity)

Poultry (one-time capacity)

Other

 No → *skip to Question 13*

1. Is your farm registered with your state as a permitted livestock operation (Illinois- CAFO, Indiana- CAFO/CFO, Michigan- CAFO, Ohio- CAFF, Wisconsin- CAFO)?

 Yes  No

1. Do you own any land that you rented out to others that *they* had under production in 2018?

 Yes: Total number of acres rented to others: acres  No

1. Was any of the land you had under production in 2018 rented from others?

 Yes: Total number of acres rented: acres → *Please answer Questions 15 to 21*

 No → *Please skip to Section 2 (next page)*

1. How many different landlords do you rent land from? landlords
2. What is the largest number of acres you rented from a single landlord? acres
3. Thinking of this largest rental contract, do you have a formal written lease agreement with the landlord?

 Yes  No

1. How long have you rented this land? years
2. For how many more years are you confident in your ability to keep renting this land?

 2 years or less

 3-5 years

 More than 5 years

1. What are the terms of this particular rental agreement?

 Cash rent, fixed

 Cash rent, flexible

 Cash rent plus a share of crop production

 Share of crop production only

1. Who is primarily responsible for day-to-day management decisions on this rented land?

 Me alone

 Primarily me, with landlord input

 Equally me and my landlord

 Primarily my landlord, with my input

 My landlord alone

 Other

*Note: This vignette experiment included varying levels of net revenue per acre, land conservation payments, wind payments, and duration of change.*

This section asks about how you might allocate your farmland if economic conditions were to change for an extended period of time. You will be asked about how you would allocate your farmland between three uses: commodity production, wind energy production, and land retired under conservation programs.

In each scenario, you will be presented with different combinations of three economic conditions:

- Farm profitability (defined as average per-acre net revenue),
- The rate of payments to set aside farmland for conservation purposes, and
- Lease rates for using farmland for wind energy production.

These economic conditions will last either 5 or 10 years, depending on the political climate.

In answering the following questions, assume that:

- Net revenue per acre is what you receive from crop production, less variable costs such as seeds/fertilizer and does not account for land, management, machinery, and equipment costs
- There is no limit to the amount of land you can enroll in the federal land retirement conservation program
- You will receive a **yearly** lease payment from a private wind energy company for each turbine installed on your land, guaranteed for the duration in the scenario
  - You cannot install more than one wind turbine per 200 acres of your land
  - You must take 1.5 acres out of production for each turbine installed, for access roads and the turbine footpad
- All prices and costs not specified in the scenario are unchanged from current levels

***Choice 1:*** Historically, net revenue, conservation payments, and wind lease payments have been roughly as shown in the table below. Suppose that you are faced with the following combination of net revenue, conservation payment, and wind turbine lease rate.

| Net revenue per acre | $215/acre |
| --- | --- |
| Conservation yearly lease payment | $120/acre |
| Wind turbine yearly lease payment | $6,000/turbine |

Given this combination of prices, how would you allocate land to production and conservation on your farm?

**acres allocated** Land in commodity production: Land set aside for conservation:

How many wind turbines would you would install, based on the rule of no more than one turbine per 200 acres and each taking 1.5 acres out of agricultural production?

| None | 1 | 2 | 3 | 4 | 5 | 6 | 7 | 8 | 9 | 10+ |  |
| --- | --- | --- | --- | --- | --- | --- | --- | --- | --- | --- | --- |
|  |  |  |  |  |  |  |  |  |  |  | «ID» |

***Choice 2:*** Now, suppose that you are faced with the following combination of net revenue,

conservation payment, wind energy lease rate, and duration of change.

| Net revenue per acre | $325/acre |
| --- | --- |
| Conservation yearly lease payment | $180/acre |
| Wind energy yearly lease payment | $7,500/turbine |
| Duration of change | 5 years |

Given this combination of prices, how would you allocate land to production and conservation on your farm?  Same as Choice 1 → *Skip to Choice 3*

**acres allocated** Land in commodity production: Land set aside for conservation:

How many wind turbines would you would install, based on the rule of no more than one turbine per 200 acres and each taking 1.5 acres out of agricultural production?

| None | 1 | 2 | 3 | 4 | 5 | 6 | 7 | 8 | 9 | 10+ |
| --- | --- | --- | --- | --- | --- | --- | --- | --- | --- | --- |
|  |  |  |  |  |  |  |  |  |  |  |

***Choice 3:*** Now, suppose that you are faced with the following combination of net revenue, conservation payments, wind energy lease rates, and duration of change.

| Net revenue per acre | $100/acre |
| --- | --- |
| Conservation yearly lease payment | $90/acre |
| Wind energy yearly lease payment | $4,500/turbine |
| Duration of change | 10 years |

Given this combination of prices, how would you allocate land to production and conservation on your farm?  Same as Choice 1 → *Skip to Choice 4*

How would you allocate your land between land in production and land in conservation?

**acres allocated** Land in commodity production: Land set aside for conservation:

How many wind turbines would you would install, based on the rule of no more than one turbine per 200 acres and each taking 1.5 acres out of agricultural production?

| None | 1 | 2 | 3 | 4 | 5 | 6 | 7 | 8 | 9 | 10+ |
| --- | --- | --- | --- | --- | --- | --- | --- | --- | --- | --- |
|  |  |  |  |  |  |  |  |  |  |  |

***Choice 4:*** Now, suppose that you are faced with the following combination of net revenue,

conservation payments, wind energy lease rates, and duration of change.

| Net revenue per acre | $0/acre |
| --- | --- |
| Conservation yearly lease payment | $60/acre |
| Wind energy yearly lease payment | $3,000/turbine |
| Duration of change | 5 years |

Given this combination of prices, how would you allocate land to production and conservation on your farm?  Same as Choice 1 → *Skip to Choice 5*

How would you allocate your land between land in production and land in conservation?

**acres allocated** Land in commodity production: Land set aside for conservation:

How many wind turbines would you would install, based on the rule of no more than one turbine per 200 acres and each taking 1.5 acres out of agricultural production?

| None | 1 | 2 | 3 | 4 | 5 | 6 | 7 | 8 | 9 | 10+ |
| --- | --- | --- | --- | --- | --- | --- | --- | --- | --- | --- |
|  |  |  |  |  |  |  |  |  |  |  |

***Choice 5:*** Now, suppose that you are faced with the following combination of net revenue, conservation payments, wind energy lease rates, and duration of change.

| Net revenue per acre | $430/acre |
| --- | --- |
| Conservation yearly lease payment | $240/acre |
| Wind energy yearly lease payment | $7,500/turbine |
| Duration of change | 10 years |

Given this combination of prices, how would you allocate land to production and conservation on your farm?  Same as Choice 1 → *Skip to Section 3*

How would you allocate your land between land in production and land in conservation?

**acres allocated** Land in commodity production: Land set aside for conservation:

How many wind turbines would you would install, based on the rule of no more than one turbine per 200 acres and each taking 1.5 acres out of agricultural production?

| None | 1 | 2 | 3 | 4 | 5 | 6 | 7 | 8 | 9 | 10+ |
| --- | --- | --- | --- | --- | --- | --- | --- | --- | --- | --- |
|  |  |  |  |  |  |  |  |  |  |  |

We are interested in how different types of farmers might respond to the same economic pressures or opportunities. The questions in this section help us understand how you approach decision-making on your farm and how you see yourself as an agricultural producer.

1. Risk management is an everyday part of farming. How would you rate your willingness to take risks in the following areas? Please *circle the number* that best represents your willingness to take risks for each.

**Better safe than sorry**


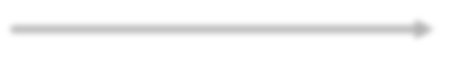


**Nothing ventured, nothing gained**

| a. generally, in day-to-day life | 0 | 1 | 2 | 3 | 4 | 5 | 6 | 7 | 8 | 9 | 10 |
| --- | --- | --- | --- | --- | --- | --- | --- | --- | --- | --- | --- |
| b. with non-farm investments | 0 | 1 | 2 | 3 | 4 | 5 | 6 | 7 | 8 | 9 | 10 |
| c. in your occupation as a farmer | 0 | 1 | 2 | 3 | 4 | 5 | 6 | 7 | 8 | 9 | 10 |

1. This next set of questions has to do with how you deal with the future. *Please circle the response that best reflects how characteristic each statement is of your approach to decision-making.*

**Very uncharacteristic**

**Uncharacteristic**

**Neutral**

**Characteristic**

**Very characteristic**

| a. I believe that a person’s day should be planned ahead each morning. | -2 | -1 | 0 | 1 | 2 |
| --- | --- | --- | --- | --- | --- |
| b. Fate determines much in my life. | -2 | -1 | 0 | 1 | 2 |
| c. When I want to achieve something, I set goals and consider specific means for reaching those goals. | -2 | -1 | 0 | 1 | 2 |
| d. Since whatever will be will be, it doesn’t really matter what I do. | -2 | -1 | 0 | 1 | 2 |
| e. Meeting tomorrow’s deadlines and doing other necessary work comes before  tonight’s play or relaxation. | -2 | -1 | 0 | 1 | 2 |
| f. You can’t really plan for the future because things change so much. | -2 | -1 | 0 | 1 | 2 |
| g. It upsets me to be late for appointments. | -2 | -1 | 0 | 1 | 2 |
| h. My life path is controlled by forces I cannot influence. | -2 | -1 | 0 | 1 | 2 |
| i. I meet my obligations to friends and authorities on time. | -2 | -1 | 0 | 1 | 2 |
| j. It doesn’t make sense to worry about the future since there is nothing I can do about it anyway. | -2 | -1 | 0 | 1 | 2 |
| k. I take each day as it is rather than try to plan it out. | -2 | -1 | 0 | 1 | 2 |
| l. Life today is too complicated; I would prefer the simpler life of the past. | -2 | -1 | 0 | 1 | 2 |
| m. I complete projects on time by making steady progress. | -2 | -1 | 0 | 1 | 2 |
| n. I make lists of things to do. | -2 | -1 | 0 | 1 | 2 |
| o. Often luck pays off better than hard work. | -2 | -1 | 0 | 1 | 2 |
| p. I keep working at difficult, uninteresting tasks if they will help me get ahead. | -2 | -1 | 0 | 1 | 2 |
|  |  |  |  | «ID» |  |

1. People have different opinions about what makes a “good farmer”. *Please circle the number that best represents how important each of the following items is to your definition of a good farmer.*


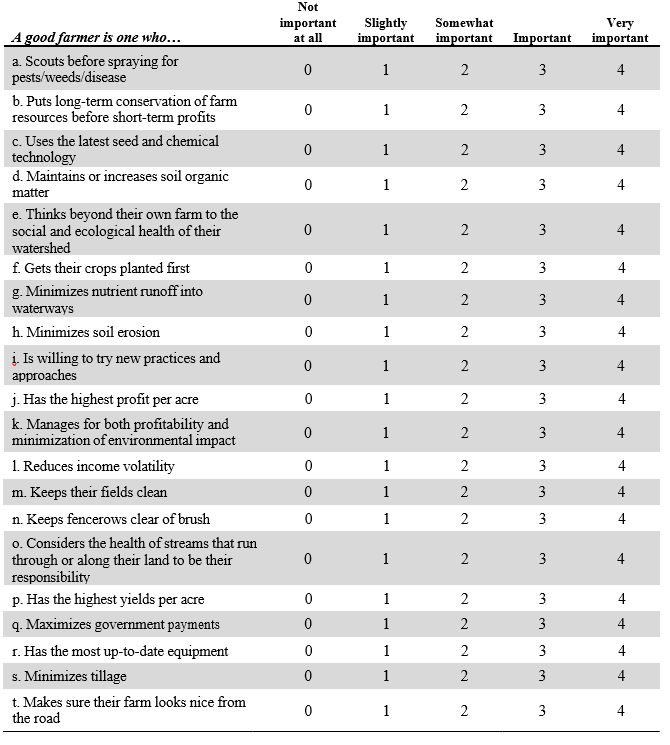


To help us understand how different farmers make decisions, please tell us a few things about yourself.

1. Are you:  Male  Female
2. How old are you? years
3. How much formal education have you completed?

 Some high school

 High school diploma or equivalent

 Some college, no degree

 Associate’s degree

 Bachelor’s degree

 Graduate or professional degree

1. How many years have you been farming? years
2. How many generations of your family farmed before you? generations
3. How many more years do you expect to personally operate your farm? years
4. When you retire, do you expect you will pass your farm down to a member of your immediate family?

 Yes  No  Not sure

1. Which of the following best describes your primary role in the farm operation? *(Check all that apply)*

 Sole manager/decision maker

 Co-manager (with my child)

 Co-manager (with my parent)

 Co-manager (with my spouse)

 Owner (but not involved w/day-to-day farm decisions)

 Other:

1. What is the ratio of total liabilities (debts) to the value of total assets for your farming operation?

 0% *(no debts)*

 1 - 20%

 21-40%

 41 – 60%

 60 – 80%

 80% or more

 Not sure

1. Dd you or your spouse receive off-farm income in 2018?

 Yes  No *(if no, skip to #12)*

1. If you or your spouse received off-farm income, approximately what proportion of your total household income came from off-farm sources in 2018?

 Less than 25%

 26-50%

 51-75%

 76% or more

1. Are you retired from an occupation other than farming?

 Yes  No

1. How much do you oppose or support wind energy development in your community?

 Strongly opposed to it

 Somewhat opposed to it

 Neither opposed nor support it

 Somewhat support it

 Strongly support it

THANK YOU! Please let us know of any other comments you have about how you currently allocate land to different uses on your farm, or how those decisions might be made in the future.

## 3.2 Study 2 Full Survey Instrument

The first set of questions have to do with your farm operation, including how your land is allocated to different uses, and how you manage your land in production. We use this information to understand how the characteristics of a farm may influence adaptation.

1. How large is your current farm operation (owned and rented land)*? Please include cropland, woodland, pasture, wasteland, land in farmsteads, and land in government programs.*

Total farm size: acres

1. How much land do you have in each category **in a typical year**? *Please enter the number of acres for each category (if none, please write 0).*

| a. Total cropland |  | acres |
| --- | --- | --- |
| i. Corn |  | acres |
| ii. Soy |  | acres |
| iii. Wheat and other small grains |  | acres |
| iv. Hay |  | acres |
| b. Livestock (e.g., land in pasture, feedlots, etc.) |  | acres |
| c. Woodland |  | acres |
| d. Land retired in conservation programs (e.g., CRP, WRP) |  | acres |
| e. Other land in dedicated conservation (e.g., filter strips,  wetlands – *not in-field practices like no-till, cover crops*)  f. Other (specify): |  | acres  acres |

1. What is the typical crop rotation for the majority of your cropland? *Please fill in the crop in each year of your typical rotation.*

Crop

Year 1:

Year 2:

Year 3:

Year 4:

Year 5:

Year 6:

1. What were the average yields for the major crops grown on your farm in 2018? *Please write-in the yield for reach crop or check the box if you did not grow that crop this past year.*

| Corn | bu/acre |  did not grow |
| --- | --- | --- |
| Soybeans | bu/acre. |  did not grow |
| Wheat | bu/acre |  did not grow |

1. Did you plant cover crops after the 2018 harvest (*not including hay or winter wheat*)? *Check one.*

 No → *skip to Question 7*

 Yes → *please list the crop(s) or mix*:

1. Approximately how many acres were planted with a cover crop after the 2018 harvest?

acres

1. On what proportion of your cropland do you use each type of tillage in your rotation?

**Conventional**: *inversion tillage and/or system that leaves less than 30% residue*  %

**Conservation**: *discing/chiseling and/or system that leaves more than 30% residue*  %

**Rotational No-Till:** *no soil tillage disturbance or full pass tillage in yearly*

*rotation with other forms of tillage leaving more than 50-60% residue*  %

**Continuous No-Till:** *never tilled, planting directly into crop residue every year*  %

1. Did you receive any income from livestock or poultry raised on your farm in 2018? *Check one.*

 No

 Yes→ How many of each did you raise or manage in 2018? *(Please fill in the # below)*

Beef cows, calves, heifers and/or feeders (total) Swine (one-time capacity)

Other (fill-in): Poultry (one-time capacity)

1. Is any of the land you currently have under production rented from others? *Check one.*

 No → *Please skip to Question 14, next page*

 Yes: Total # of acres rented: acres → *Please answer Questions 10 to 13*

1. For how many more years are you confident in your ability to keep renting this land? *Check one.*

 less than 5  5-10 years  10 + years

1. To what extent is your landlord involved in cropping decisions (e.g., fertilizer rates, crops grown)? *Check one.*

 Not at all  Very little  Somewhat  To a great extent

1. To what extent is your landlord involved in conservation decisions (e.g., cover crops, buffers)?

 Not at all  Very little  Somewhat  To a great extent

1. Are your management decisions more constrained on rented land than on the land you own?

 Not at all  Very little  Somewhat  To a great extent

1. The weather this past spring may have delayed your planting. When do you typically complete planting in the spring, and when did you complete your planting this past spring?

Month/day *typical year* Month/day *this year* Corn Soybeans

1. Do you have crop insurance for any of your planted acres? *Check one.*

 No → *Please skip to Question 18*

 Yes → *Please answer Questions 16 & 17*

1. What type of insurance do you have for the majority of your acres?

*Check one:*  Yield insurance  Revenue insurance  No insurance

*Check one:*  Basic units  Optional units  Enterprise units  Whole farm units

1. What coverage level is the majority of your crop insurance? *Check one.*

 50% coverage (CAT)  70% coverage

 55% coverage  75% coverage

 60% coverage  80% coverage

 65% coverage  85% coverage

1. Approximately how many acres of your land in production are artificially drained by tile?

acres

1. Approximately how many of your planted acres (owned and rented) is in each soil type below?

Predominantly clay: acres Predominantly sand: acres Predominantly loam: acres Other (specify): acres

1. Is any of your land highly erodible (HEL)? *Check one.*

 No  Yes → (*fill in approximate acres*)

 Unsure

*Note: In the vignette experiment, participants were randomly presented with one of 36 combinations of parameters. Parameters were also customized to the typical rainfall, planting/harvest dates, and state conservation retirement payments in their state.*

In this section, you will be asked to consider potential future weather conditions and indicate how you might adapt on your farm. Each scenario will include different combinations of planting dates, average growing season rainfall, and harvesting dates that are expected to occur with a particular likelihood over the next 10 years. Each scenario will also include a proposed federal land retirement conservation payment that would be available during that same 10 year period.

In considering the following scenarios, assume that:

- - All prices and costs not specified in the scenario (e.g., commodity and input prices) are unchanged from current levels
  - There is no limit to the amount of land you can enroll in the federal land retirement conservation program, but the land would be in contract for the full 10 years.
  - The chance of a frost is the same as in a typical growing year
  - Years not characterized by the scenario will be the same as you’ve experienced in the past ten years.

Finally, know that over the past 10 years, the normal planting time for corn in Illinois was April 21, and harvesting was typically completed by September 23. During this same time period, rainfall during a typical growing season (April 1^st^ to November 1^st^) was 30 inches, and federal land retirement conservation payments were typically $220 per acre.

***Choice 1. Please read the following scenario and indicate what changes (if any) you would make to your operation.*** Suppose that instead of current payment rates, you would receive **$320** per acre enrolled in a land retirement program. Additionally, suppose that **five out of the next ten** years were characterized by the following weather conditions:

| **Planting date** | March 22 (*30 days earlier than usual*) |
| --- | --- |
| **Average rainfall during the growing season** | 15” (*15" less than usual*) |
| **Harvesting date** | November 12 (*50 days later than usual*) |

Under these conditions, what changes would you be likely to make to your farm operation?

*Please check all that apply or select the last option if you would not make any of these changes.*

 Change my rotation to allow for double cropping

 Install more tile drainage on my farm

 Change my crop insurance coverage (*select one option in each row if applicable*)

 Yield insurance  Revenue insurance

 Increase coverage  Decrease coverage

 Increase my use of no-till/conservation tillage

 Enroll more of my land in conservation programs  acres

 Invest in new or additional irrigation on my farm

 I would not make any of these changes

***Choice 2:*** Now, suppose that instead of current payment rates, you would receive **$120** per acre enrolled in a land retirement program. Additionally, suppose that **eight out of the next ten** years were characterized by the following weather conditions:

| **Planting date** | March 22 (*30 days earlier than usual*) |
| --- | --- |
| **Average rainfall during the growing season** | 15” (*15" less than usual*) |
| **Harvesting date** | November 12 (*50 days later than usual*) |

1. Under these conditions, what changes would you be likely to make to your farm operation?

*Please check all that apply or select the last option if you would not make any of these changes.*

 Change my rotation to allow for double cropping

 Install more tile drainage on my farm

 Change my crop insurance coverage (*select one option in each row if applicable*)

 Yield insurance  Revenue insurance

 Increase coverage  Decrease coverage

 Increase my use of no-till/conservation tillage

 Enroll more of my land in conservation programs  acres

 Invest in new or additional irrigation on my farm

 I would not make any of these changes

***Choice 3:*** Now, suppose that instead of current payment rates, you would receive **$320** per acre enrolled in a land retirement program. Additionally, suppose that **eight out of the next ten** years were characterized by the following weather conditions:

| **Planting date** | March 22 (*30 days earlier than usual*) |
| --- | --- |
| **Average rainfall during the growing season** | 30” (*No change from baseline*) |
| **Harvesting date** | September 23 (*No change from baseline*) |

1. Under these conditions, what changes would you be likely to make to your farm operation?

*Please check all that apply or select the last option if you would not make any of these changes.*

 Change my rotation to allow for double cropping

 Install more tile drainage on my farm

 Change my crop insurance coverage (*select one option in each row if applicable*)

 Yield insurance  Revenue insurance

 Increase coverage  Decrease coverage

 Increase my use of no-till/conservation tillage

 Enroll more of my land in conservation programs  acres

 Invest in new or additional irrigation on my farm

 I would not make any of these changes


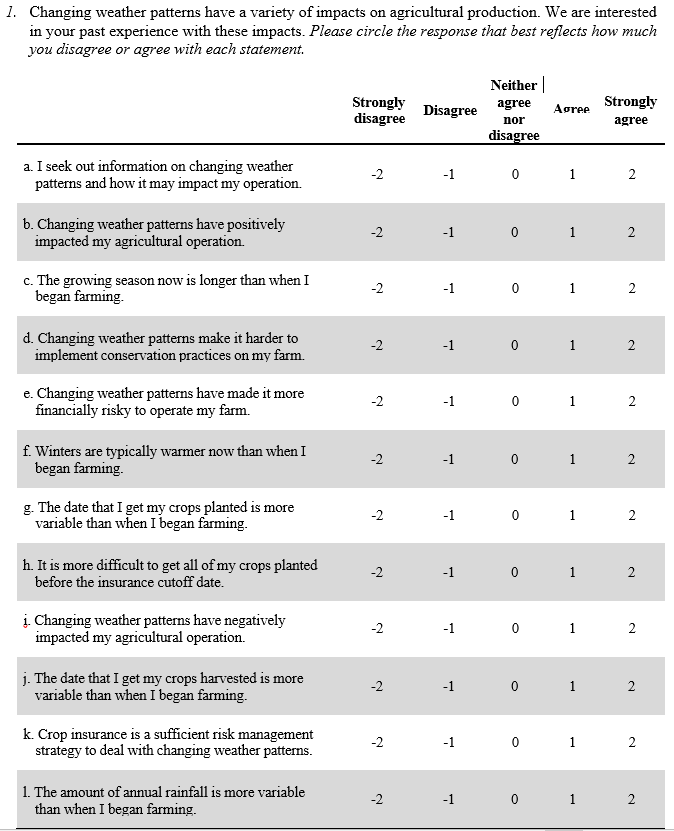


1. Changing weather patterns are expected to continue in the future. *Please circle the response that best indicates how concerned you are about each of the following potential impacts to agricultural production in your state over the next 10 to 20 years.*

| ***I am concerned about…*** | **Not**  **concerned at all** | **Slightly concerned** | **Somewhat concerned** | **Concerned** | **Very concerned** |
| --- | --- | --- | --- | --- | --- |
| a. More frequent extreme rainfall events | 0 | 1 | 2 | 3 | 4 |
| b. Increasing pressure on crops from pests | 0 | 1 | 2 | 3 | 4 |
| c. More frequent droughts | 0 | 1 | 2 | 3 | 4 |
| d. More frequent flooding | 0 | 1 | 2 | 3 | 4 |
| e. Increased heat stress on crops | 0 | 1 | 2 | 3 | 4 |
| f. Fewer days for planting | 0 | 1 | 2 | 3 | 4 |
| g. Fewer days for harvesting | 0 | 1 | 2 | 3 | 4 |
| h. Higher nighttime temperatures | 0 | 1 | 2 | 3 | 4 |
| i. Fewer days below freezing in the winter | 0 | 1 | 2 | 3 | 4 |
| j. Increasing crop insurance premiums | 0 | 1 | 2 | 3 | 4 |
| k. Increased soil erosion | 0 | 1 | 2 | 3 | 4 |

1. During a typical year, how often do you receive information from the following sources about changing weather patterns, their impacts and/or strategies for adaptation? *Please circle the number that best reflects how frequently you receive information from each source, and check the box if this information has been useful to your decision making.*

| **I receive information from …** | **Never** | **Rarely** | **Sometimes** | **Frequently** | **Very**  **frequently** | **Useful**  **source** |
| --- | --- | --- | --- | --- | --- | --- |
| a. The land grant Extension service | 0 | 1 | 2 | 3 | 4 |  |
| c. Farm bureau | 0 | 1 | 2 | 3 | 4 |  |
| d. Your local conservation district | 0 | 1 | 2 | 3 | 4 |  |
| e. Your crop adviser/consultant | 0 | 1 | 2 | 3 | 4 |  |
| f. Your fertilizer applicator or retailer | 0 | 1 | 2 | 3 | 4 |  |
| g. USDA NRCS | 0 | 1 | 2 | 3 | 4 |  |
| h. Professional/industry magazines | 0 | 1 | 2 | 3 | 4 |  |
| i. Commodity groups | 0 | 1 | 2 | 3 | 4 |  |
| j. A family member or farm partner | 0 | 1 | 2 | 3 | 4 |  |
| k. Other farmers in your community | 0 | 1 | 2 | 3 | 4 |  |
| l. Other: | 0 | 1 | 2 | 3 | 4 |  |

1. Changing weather conditions may require you to change the way you do some things on your farm. *First, indicate whether you have already done the following on your farm* ***in an attempt to adapt to changing weather conditions***. *Whether or not you have already done these activities in response to the changing weather,* ***please indicate how likely you are to do each of the following in the next ten years to minimize the expected impacts****.*

| **I have already done this** | | **Not likely at all** | **Not likely** | **Some what likely** | **Likely** | **Very likely** |
| --- | --- | --- | --- | --- | --- | --- |
| a. Plant more resilient varieties of crops that I already grow |  | 0 | 1 | 2 | 3 | 4 |
| b. Hire additional labor on my farm |  | 0 | 1 | 2 | 3 | 4 |
| c. Change the type of crop insurance I buy (e.g., from yield to revenue insurance or vice versa) |  | 0 | 1 | 2 | 3 | 4 |
| d. Install more tile drainage on my fields |  | 0 | 1 | 2 | 3 | 4 |
| e. Plant cover crops to improve soil health |  | 0 | 1 | 2 | 3 | 4 |
| f. Sell my farm |  | 0 | 1 | 2 | 3 | 4 |
| g. Apply more fertilizer on my crops |  | 0 | 1 | 2 | 3 | 4 |
| h. Rent out my land |  | 0 | 1 | 2 | 3 | 4 |
| i. Buy new equipment or technology to reduce planting time |  | 0 | 1 | 2 | 3 | 4 |
| j. Buy new equipment or technology to reduce harvesting time |  | 0 | 1 | 2 | 3 | 4 |
| k. Increase my crop insurance coverage level |  | 0 | 1 | 2 | 3 | 4 |
| l. Change my crop rotation to allow for double cropping |  | 0 | 1 | 2 | 3 | 4 |
| m. Install filtering practices on my fields |  | 0 | 1 | 2 | 3 | 4 |
| n. Apply more pesticides on my crops |  | 0 | 1 | 2 | 3 | 4 |
| o. Rent more acres |  | 0 | 1 | 2 | 3 | 4 |
| p. Outsource some activities on my farm (e.g., fertilizer application, etc.) |  | 0 | 1 | 2 | 3 | 4 |
| q. Look for work off my farm |  | 0 | 1 | 2 | 3 | 4 |
| r. Change the terms of my rental contract (e.g., from cash to a crop share contract) |  | 0 | 1 | 2 | 3 | 4 |
| s. Change my tillage practices (e.g., adopt no-till or conservation tillage on my farm) |  | 0 | 1 | 2 | 3 | 4 |


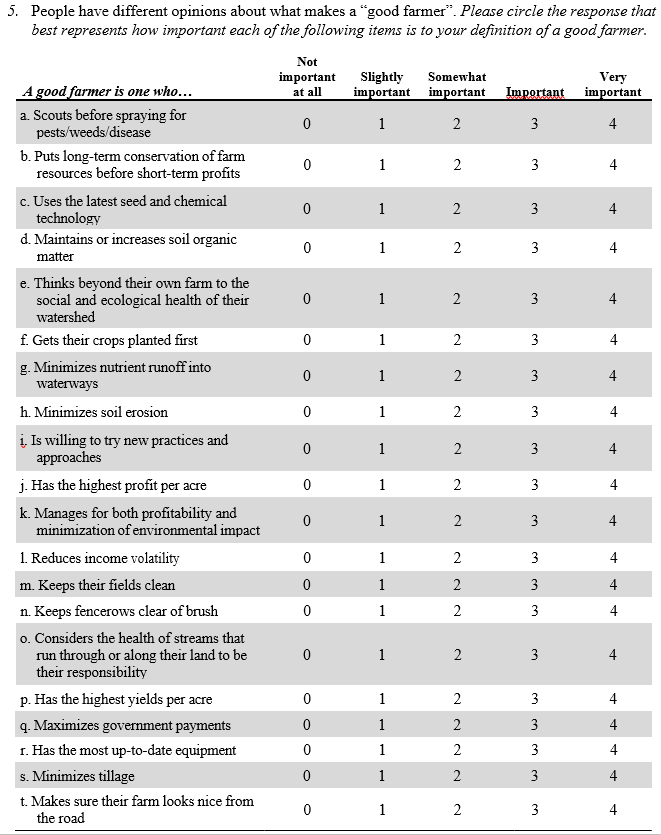


To help us understand how different farmers make decisions, please tell us a few things about yourself.

1. Are you:  Male  Female
2. How old are you? years
3. How much formal education have you completed? *Please check one.*

 Some high school

 High school diploma or equivalent

 Some college, no degree

 Associate’s degree

 Bachelor’s degree

 Graduate or professional degree

1. How many years have you been farming? years
2. How many generations of your family farmed before you? generations
3. How many more years do you expect to personally operate your farm? years
4. When you retire, do you expect you will pass your farm down to a member of your immediate or extended family? *Please check one.*

 No  Yes  Not sure

1. Which of the following best describes your primary role in the farm operation? *Check all that apply*

 Sole manager/decision maker

 Co-manager (with my child)

 Co-manager (with my parent)

 Co-manager (with my spouse)

 Owner (but not involved w/day-to-day farm decisions)

 Other:

1. Did you or your spouse receive off-farm income in 2018? *Please check one.*

 No *(if no, skip to #11)*  Yes

1. Approximately what proportion of your total household income came from off-farm sources in 2018? *Please check one.*

 Less than 25%

 26-50%

 51-75%

 76% or more

1. Which of the following statements about a changing climate comes closest to your view? *Please check one.*

 The climate is not changing

 The climate is changing, and is mostly caused by natural changes in the environment

 The climate is changing, and is mostly caused by human activities

 Unsure

1. When considering scientific research about whether the climate is changing or not, which of the following statements comes closest to your own view? *Please check one.*

 Most scientists think the climate is changing.

 Most scientists think the climate is not changing.

 There is a lot of disagreement among scientists about whether the climate is changing.

 Not sure

THANK YOU! Please let us know of any other comments you have about the impacts of changing weather patterns on your agricultural operation, or how changing weather patterns might impact your operation in the future.

# 4. Stata Code for All Analyses

## 4.1 Study 1 Code

### 4.1.1 Code for Computing Variables

****In-Field Adaptation Index****

***1. Tillage weights

**First, create variables that represent percent of each type of tillage

gen conventional_till = conventional_till_percent/100

gen consv_till = conservation_till_percent/100

gen rotate_till = rotation_no_till_percent/100

gen no_till = no_till_percent/100

*Determine who did not answer properly based on proportion of land in each till exceeding 1.0 (which is impossible)

egen include_soilindex = rowtotal( conventional_till consv_till rotate_till no_till), missing

*Note that missing on all values stays missing

*If zero percent across all tillage types, then do not include

recode include_soilindex (0=0) (.25/1 = 1) (2=0)

*Now let's make the till weights and sum, if including in index

gen consv_till_weight= (consv_till*0.33)

gen rotate_till_weight = (rotate_till*0.47)

gen no_till_weight= (no_till*0.93)

egen till_weight = rowtotal(consv_till_weight rotate_till_weight no_till_weight) if include_soilindex==1

***2. Weight presence of small grains. “year_#_grains” variables were coded the presence of conservation crops/small grains being planted in the farmer’s fields each year. If small grain or a conservation crop was present that year, coded as “1”, if absent, coded as “0”.

*Add up the number of years a small grain was used to get the variable “conscroprot_totyrs”.

egen cons_crop_totalyrs = rowtotal( year_1_grains year_2_grains year_3_grains year_4_grains year_5_grains), missing

*Total is then divided by total years (n) for total proportion of years that a small grain was used.

gen propyrs_conscrop = cons_crop_totalyrs/5

*Weight proportion of years with small grains by impact on nutrient reduction.
gen smallgrains_weight = (propyrs_conscrop *0.42) if include_soilindex==1

***3. Weight proportion of land in cover crops.
**If indicated that they did not plant cover crop in the previous question, missing was coded as zero

gen prop_covercrop = cvrcrop_land_pct

recode prop_covercrop (.=0) if cvrcrop==0

**Divide by 100 to reach proportion, then weight by estimated reduction in Nitrogen.

gen cvrcrops_weight = ((prop_covercrop/100)*0.30) if include_soilindex==1

***4. Add up weight of till practices, small grains, and cover crops. Note that if missing on all values, total is set to missing (not zero).

egen nutrientreduct_index = rowtotal( till_weight smallgrains_weight cvrcrops_weight), missing

**Exclude folks who are not to be included, as per above calculation

recode nutrientreduct_index (0/*max* *value*=.) if include_soilindex==0

****Scale variables****

**Future Time Perspective

egen ftp = rowmean(future_a future_c future_e future_g future_i future_m future_n future_p)

egen zftp = std(ftp)

**Conservationist norms

egen cons_id = rowmean(good_farmer_a good_farmer_b good_farmer_d good_farmer_e good_farmer_g good_farmer_h good_farmer_k good_farmer_o good_farmer_s)

egen zcons_id = std( cons_id)

**Productivist norms

gen prod_id = rowmean(good_farmer_c good_farmer_f good_farmer_j good_farmer_m good_farmer_n good_farmer_p good_farmer_r good_farmer_t )

egen zprod_id = std( prod_id)

****SES Variables****

egen zedu = std(edu)

gen log_cropland= log(total_cropland)

egen zlog_cropland= std(log_cropland)

****Control Variables****

**Yield

*Index of corn and soy yield by taking average z-score; so yield average is combination of yields relative to others in the sample.

egen zcorn_yield = std( corn_yield)

egen zsoy_yield = std( soy_yield)

egen cornsoy_yield= rowmean( zcorn_yield zsoy_yield)

egen zcornsoy_yield = std( cornsoy_yield)

**Age

egen zage = std(age)

### 4.1.2 Code for Confirmatory Factor Analyses

1. Future Time Perspective

**Model 1

sem Future-> future_a future_c future_e future_g future_i future_k future_m future_n future_p, nocapslatent latent(Future) standardized

estat gof, stats(all)

estat eqgof

estat mindices

**Model 2

sem Future-> future_a future_c future_e future_g future_i future_m future_n future_p, nocapslatent latent(Future) standardized

**Model 3

sem Future-> future_a future_c future_e future_g future_i future_m future_n future_p, nocapslatent latent(Future) standardized cov(e.future_a*e.future_n)

**Model 4, accepted as final model

sem Future-> future_a future_c future_e future_g future_i future_m future_n future_p, nocapslatent latent(Future) standardized cov(e.future_a*e.future_n) cov(e.future_g*e.future_i)

2. Conservationist norms

**Model 1:

sem Cons_ID -> good_farmer_a good_farmer_b good_farmer_d good_farmer_e good_farmer_g good_farmer_h good_farmer_k good_farmer_o good_farmer_s, nocapslatent latent(Cons_ID) standardized

**Model 2, accepted as final model:

sem Cons_ID -> good_farmer_a good_farmer_b good_farmer_d good_farmer_e good_farmer_g good_farmer_h good_farmer_k good_farmer_o good_farmer_s, nocapslatent latent(Cons_ID) standardized cov(e.good_farmer_g*e.good_farmer_h)

3. Productivist norms

**Model 1

sem Prod_ID -> good_farmer_c good_farmer_f good_farmer_j good_farmer_m good_farmer_n good_farmer_p good_farmer_r good_farmer_t , nocapslatent latent(Prod_ID) standardized

**Model 2

sem Prod_ID -> good_farmer_c good_farmer_f good_farmer_j good_farmer_m good_farmer_n good_farmer_p good_farmer_r good_farmer_t , nocapslatent latent(Prod_ID) standardized cov(e.good_farmer_j*e.good_farmer_p)

**Model 3, accepted as final model:

sem Prod_ID -> good_farmer_c good_farmer_f good_farmer_j good_farmer_m good_farmer_n good_farmer_p good_farmer_r good_farmer_t , nocapslatent latent(Prod_ID) standardized cov(e.good_farmer_j*e.good_farmer_p) cov(e.good_farmer_m*e.good_farmer_n)

### 4.1.3 Code for Structural Equation Model Predicting In-Field Adaptation

****Notes****
***SEM predicting in-field adaptation practices from future time perspective, socioeconomic factors, and identity. All exogenous variables allowed to correlate.

***Allowed error covariance between “a good farmer minimizes tillage” and the index of in-field adaptation practices, as this outcome variable includes tillage in the index.

***Error terms of Productivist and Conservationist indentity were allowed to covary, as these constructs are known to correlate.

***Because older persons tend to have higher productivist norms, a path was added from age to productivist norms.

***Medsem used to test for mediation. The indirect effect of future time perspective on in-field adapation through Conservationist norms was tested.

***Model 1:

sem (Future-> future_a future_c future_e future_g future_i future_m future_n future_p) (Cons_ID -> good_farmer_a good_farmer_b good_farmer_d good_farmer_e good_farmer_g good_farmer_h good_farmer_k good_farmer_o good_farmer_s) (Prod_ID -> good_farmer_c good_farmer_f good_farmer_j good_farmer_m good_farmer_n good_farmer_p good_farmer_r good_farmer_t) (nutrientreduct_index<- Future zlog_cropland zedu livestock_yn Cons_ID Prod_ID zcornsoy_yield rents_yes zage ) (Cons_ID<-Future) (Prod_ID<-zage) , nocapslatent latent(Future Cons_ID Prod_ID) standardized cov(e.future_a*e.future_n) cov(e.future_g*e.future_i) cov(e.good_farmer_g*e.good_farmer_h) cov(e.good_farmer_j*e.good_farmer_p) cov(e.good_farmer_m*e.good_farmer_n) cov(e.good_farmer_s*e.nutrientreduct_index) cov(e.Cons_ID*e.Prod_ID)

**This model fit was not adequate. Therefore, modification indices were examined. The largest modification index was examined, and if appropriate, allowed. If not, the next-largest MI was examined, and so forth.

estat gof, stats(all)

estat eqgof

estat mindices

***Model 2:

sem (Future-> future_a future_c future_e future_g future_i future_m future_n future_p) (Cons_ID -> good_farmer_a good_farmer_b good_farmer_d good_farmer_e good_farmer_g good_farmer_h good_farmer_k good_farmer_o good_farmer_s) (Prod_ID -> good_farmer_c good_farmer_f good_farmer_j good_farmer_m good_farmer_n good_farmer_p good_farmer_r good_farmer_t) (nutrientreduct_index<- Future zlog_cropland zedu livestock_yn Cons_ID Prod_ID zcornsoy_yield rents_yes zage ) (Cons_ID<-Future) (Prod_ID<-zage) , nocapslatent latent(Future Cons_ID Prod_ID) standardized cov(e.future_a*e.future_n) cov(e.future_g*e.future_i) cov(e.good_farmer_g*e.good_farmer_h) cov(e.good_farmer_j*e.good_farmer_p) cov(e.good_farmer_m*e.good_farmer_n) cov(e.good_farmer_s*e.nutrientreduct_index) cov(e.Cons_ID*e.Prod_ID) cov(e.good_farmer_k*e.good_farmer_j)

***Model 3:

sem (Future-> future_a future_c future_e future_g future_i future_m future_n future_p) (Cons_ID -> good_farmer_a good_farmer_b good_farmer_d good_farmer_e good_farmer_g good_farmer_h good_farmer_k good_farmer_o good_farmer_s) (Prod_ID -> good_farmer_c good_farmer_f good_farmer_j good_farmer_m good_farmer_n good_farmer_p good_farmer_r good_farmer_t) (nutrientreduct_index<- Future zlog_cropland zedu livestock_yn Cons_ID Prod_ID zcornsoy_yield rents_yes zage ) (Cons_ID<-Future) (Prod_ID<-zage zcornsoy_yield ) , nocapslatent latent(Future Cons_ID Prod_ID) standardized cov(e.future_a*e.future_n) cov(e.future_g*e.future_i) cov(e.good_farmer_g*e.good_farmer_h) cov(e.good_farmer_j*e.good_farmer_p) cov(e.good_farmer_m*e.good_farmer_n) cov(e.good_farmer_s*e.nutrientreduct_index) cov(e.Cons_ID*e.Prod_ID) cov(e.good_farmer_k*e.good_farmer_j)

***Model 4:

sem (Future-> future_a future_c future_e future_g future_i future_m future_n future_p) (Cons_ID -> good_farmer_a good_farmer_b good_farmer_d good_farmer_e good_farmer_g good_farmer_h good_farmer_k good_farmer_o good_farmer_s) (Prod_ID -> good_farmer_c good_farmer_f good_farmer_j good_farmer_m good_farmer_n good_farmer_p good_farmer_r good_farmer_t) (nutrientreduct_index<- Future zlog_cropland zedu livestock_yn Cons_ID Prod_ID zcornsoy_yield rents_yes zage ) (Cons_ID<-Future) (Prod_ID<-zage zcornsoy_yield ) , nocapslatent latent(Future Cons_ID Prod_ID) standardized cov(e.future_a*e.future_n) cov(e.future_g*e.future_i) cov(e.good_farmer_g*e.good_farmer_h) cov(e.good_farmer_j*e.good_farmer_p) cov(e.good_farmer_m*e.good_farmer_n) cov(e.good_farmer_s*e.nutrientreduct_index) cov(e.Cons_ID*e.Prod_ID) cov(e.good_farmer_k*e.good_farmer_j) cov(e.good_farmer_m*e.good_farmer_r)

***Model 5:

sem (Future-> future_a future_c future_e future_g future_i future_m future_n future_p) (Cons_ID -> good_farmer_a good_farmer_b good_farmer_d good_farmer_e good_farmer_g good_farmer_h good_farmer_k good_farmer_o good_farmer_s) (Prod_ID -> good_farmer_c good_farmer_f good_farmer_j good_farmer_m good_farmer_n good_farmer_p good_farmer_r good_farmer_t) (nutrientreduct_index<- Future zlog_cropland zedu livestock_yn Cons_ID Prod_ID zcornsoy_yield rents_yes zage ) (Cons_ID<-Future) (Prod_ID<-zage zcornsoy_yield ) , nocapslatent latent(Future Cons_ID Prod_ID) standardized cov(e.future_a*e.future_n) cov(e.future_g*e.future_i) cov(e.good_farmer_g*e.good_farmer_h) cov(e.good_farmer_j*e.good_farmer_p) cov(e.good_farmer_m*e.good_farmer_n) cov(e.good_farmer_s*e.nutrientreduct_index) cov(e.Cons_ID*e.Prod_ID) cov(e.good_farmer_k*e.good_farmer_j) cov(e.good_farmer_m*e.good_farmer_r)

***Model 6:

sem (Future-> future_a future_c future_e future_g future_i future_m future_n future_p) (Cons_ID -> good_farmer_a good_farmer_b good_farmer_d good_farmer_e good_farmer_g good_farmer_h good_farmer_k good_farmer_o good_farmer_s) (Prod_ID -> good_farmer_c good_farmer_f good_farmer_j good_farmer_m good_farmer_n good_farmer_p good_farmer_r good_farmer_t) (nutrientreduct_index<- Future zlog_cropland zedu livestock_yn Cons_ID Prod_ID zcornsoy_yield rents_yes zage ) (Cons_ID<-Future) (Prod_ID<-zage zcornsoy_yield ) , nocapslatent latent(Future Cons_ID Prod_ID) standardized cov(e.future_a*e.future_n) cov(e.future_g*e.future_i) cov(e.good_farmer_g*e.good_farmer_h) cov(e.good_farmer_j*e.good_farmer_p) cov(e.good_farmer_m*e.good_farmer_n) cov(e.good_farmer_s*e.nutrientreduct_index) cov(e.Cons_ID*e.Prod_ID) cov(e.good_farmer_k*e.good_farmer_j) cov(e.good_farmer_m*e.good_farmer_r) cov(e.good_farmer_d*e.good_farmer_c)

***Model 7, accepted as final model:

sem (Future-> future_a future_c future_e future_g future_i future_m future_n future_p) (Cons_ID -> good_farmer_a good_farmer_b good_farmer_d good_farmer_e good_farmer_g good_farmer_h good_farmer_k good_farmer_o good_farmer_s) (Prod_ID -> good_farmer_c good_farmer_f good_farmer_j good_farmer_m good_farmer_n good_farmer_p good_farmer_r good_farmer_t) (nutrientreduct_index<- Future zlog_cropland zedu livestock_yn Cons_ID Prod_ID zcornsoy_yield rents_yes zage ) (Cons_ID<-Future) (Prod_ID<-zage zcornsoy_yield ) , nocapslatent latent(Future Cons_ID Prod_ID) standardized cov(e.future_a*e.future_n) cov(e.future_g*e.future_i) cov(e.good_farmer_g*e.good_farmer_h) cov(e.good_farmer_j*e.good_farmer_p) cov(e.good_farmer_m*e.good_farmer_n) cov(e.good_farmer_s*e.nutrientreduct_index) cov(e.Cons_ID*e.Prod_ID) cov(e.good_farmer_k*e.good_farmer_j) cov(e.good_farmer_m*e.good_farmer_r) cov(e.good_farmer_d*e.good_farmer_c) cov(e.good_farmer_h*e.good_farmer_s)

****Testing mediation model using medsem

medsem, indep(Future) med(Cons_ID) dep( nutrientreduct_index) mcreps(500) rit rid zlc stand

## 4.2 Study 2 Code

### 4.2.1 Code for Computing Variables

*Coding clay acres such that if the person answered any of the soil type questions, missing on clay is coded as zero

egen answered_soil_yes = rowtotal( clay_acres loam_acres sand_acres othersoil_acres ), missing

recode answered_soil_yes (.=0) (20/10750=1)

gen clay_acres_recode = clay_acres

recode clay_acres_recode (.=0) if answered_soil_yes==1

*Coding percent of cropland acres that are clay; values over 1 are impossible and therefore coded as missing.

gen clay_pct = clay_acres_recode/ cropland_acres

gen clay_pct_recode = clay_pct

recode clay_pct_recode (1.005/10=.)

*Index of corn and soy yield by taking average z-score; so yield average is combination of yields relative to others in the sample.

egen zcorn_yield = std( corn_yield)

egen zsoy_yield = std( soy_yield)

egen cornsoy_yield= rowmean( zcorn_yield zsoy_yield)

egen zcornsoy_yield = std( cornsoy_yield)

*Z-scoring other variables of interest

egen zage = std(age)

egen zedu = std(edu)

egen ztotal_cropland= std(total_cropland)

egen zclay_pct = std( clay_pct_recode)

egen zcons_pmt_baseline = std(cons_pmt_baseline)

**Scale variables

*Future Weather Concern

egen future_wx_conc = rowmean(q32a q32b q32c q32d q32e q32f q32g q32h q32i q32k)

egen zfuture_wx_conc = std( future_wx_conc)

*Conservationist norms

egen cons_id = rowmean( q35a q35b q35d q35e q35g q35h q35k q35o q35s)

egen zcons_id = std( cons_id)

*Productivist norms

egen prod_id = rowmean( q35c q35f q35j q35m q35n q35p q35r q35t)

egen zprod_id = std( prod_id)

**Vignette DVs

*Coding for whether the person participated in each choice experiment round

egen participate_c1 = rowtotal( double_yes_c1 tile_yes_c1 upins_yes_c1 changeins_c1 changeinstype_c1 notill_yes_c1 cons_yes_c1 cons_acres_c1 irrigate_yes_c1 donothing_c1), missing

egen participate_c2 = rowtotal( double_yes_c2 tile_yes_c2 upins_yes_c2 changeins_c2 changeinstype_c2 notill_yes_c2 cons_yes_c2 cons_acres_c2 irrigate_yes_c2 donothing_c2), missing

egen participate_c3 = rowtotal( double_yes_c3 tile_yes_c3 upins_yes_c3 changeins_c3 changeinstype_c3 notill_yes_c3 cons_yes_c3 cons_acres_c3 irrigate_yes_c3 donothing_c3), missing

recode participate_c1 participate_c2 participate_c3 (.=0) (0/6504=1)

*Recoding the DVs such that if a person participated in that round, missing is coded as zero.

recode double_yes_c1 tile_yes_c1 changeins_c1 notill_yes_c1 cons_yes_c1 cons_acres_c1 irrigate_yes_c1 donothing_c1 (.=0) if participate_c1==1

recode double_yes_c2 tile_yes_c2 changeins_c2 notill_yes_c2 cons_yes_c2 cons_acres_c2 irrigate_yes_c2 donothing_c2 (.=0) if participate_c2==1

recode double_yes_c3 tile_yes_c3 changeins_c3 notill_yes_c3 cons_yes_c3 cons_acres_c3 irrigate_yes_c3 donothing_c3 (.=0) if participate_c3==1

*Determining Group

egen group = group(prob_n1 cons_pmt_change_1 plant_change_1 rain_change_1 harvest_change_1 prob_n2 cons_pmt_change_2 plant_change_2 rain_change_2 harvest_change_2 prob_n3 cons_pmt_change_3 plant_change_3 rain_change_3 harvest_change_3), label

### 4.2.2 Code for Hierarchical Mixed-effects Logistic Regression Models

1. Land Conservation Retirement

**Full model with all fixed effects predictors

meqrlogit cons_yes_c cons_pmt_change_ rain_less_ rain_more_ plant_early_ plant_late_ harvest_early_ harvest_late_ prob_n scenario zfuture_wx_conc zlog_cropland_acres zedu livestock_yes zcons_id zprod_id zcornsoy_yield zclay_pct rents_yes insurance_yes zage zcons_pmt_baseline|| group:

*Post-estimation commands; note that these were completed for all models

*linear prediction of fixed portion of model

predict xb, xb

*standard error for fixed-portion linear prediction

predict stdp, stdp

*check that predicted versus error is random-looking
scatter xb stdp

**Reduced model; non-significant parameters eliminated, but still controlling for vignette parameters

meqrlogit cons_yes_c cons_pmt_change_ rain_less_ rain_more_ plant_early_ plant_late_ harvest_early_ harvest_late_ prob_n scenario zfuture_wx_conc zedu zcons_id zprod_id cons_pmt_baseline|| group:

2. No-Till/Conservation Tillage

**Full model with all fixed effects predictors

meqrlogit notill_yes_c cons_pmt_change_ rain_less_ rain_more_ plant_early_ plant_late_ harvest_early_ harvest_late_ prob_n scenario zfuture_wx_conc zlog_cropland_acres zedu livestock_yes zcons_id zprod_id zcornsoy_yield zclay_pct rents_yes insurance_yes zage zcons_pmt_baseline|| group:

**Reduced model; non-significant parameters eliminated, but still controlling for vignette parameters

meqrlogit notill_yes_c cons_pmt_change_ rain_less_ rain_more_ plant_early_ plant_late_ harvest_early_ harvest_late_ prob_n scenario zfuture_wx_conc zlog_cropland_acres zedu livestock_yes zprod_id || group:

**Full model with participant ID as additional random intercept

meqrlogit notill_yes_c cons_pmt_change_ rain_less_ rain_more_ plant_early_ plant_late_ harvest_early_ harvest_late_ prob_n scenario zfuture_wx_conc zlog_cropland_acres zedu livestock_yes zcons_id zprod_id zcornsoy_yield zclay_pct rents_yes insurance_yes zage zcons_pmt_baseline|| group: || Survey_ID:

3. Tile Drainage

**Full model with all fixed effects predictors

meqrlogit tile_yes_c cons_pmt_change_ rain_less_ rain_more_ plant_early_ plant_late_ harvest_early_ harvest_late_ prob_n scenario zfuture_wx_conc zlog_cropland_acres zedu livestock_yes zcons_id zprod_id zcornsoy_yield zclay_pct rents_yes insurance_yes zage zcons_pmt_baseline|| group:

*Save estimates

matrix b= e(b)

*Add random effect of rain

xtmelogit tile_yes_c prob_n cons_pmt_change_ plant_early_ plant_late_ rain_more_ rain_less_ harvest_late_ harvest_early scenario zcons_id zprod_id zfuture_wx_conc zclay_pct zcornsoy_yield zedu zage insurance_yes zcons_pmt_baseline zlog_cropland_acres rents_yes livestock_yes || group: rain_more_ rain_less_, from(b, skip)

*Save estimates again

matrix b= e(b)

**Reduced model; non-significant parameters eliminated, but still controlling for vignette parameters

meqrlogit tile_yes_c cons_pmt_change_ rain_less_ rain_more_ plant_early_ plant_late_ harvest_early_ harvest_late_ prob_n scenario zfuture_wx_conc zlog_cropland_acres zedu livestock_yes zclay_pct zage || group: rain_more_ rain_less_ , from(b, skip)

4. More Irrigation

**Full model with all fixed effects predictors

meqrlogit irrigate_yes_c cons_pmt_change_ rain_less_ rain_more_ plant_early_ plant_late_ harvest_early_ harvest_late_ prob_n scenario zfuture_wx_conc zlog_cropland_acres zedu livestock_yes zcons_id zprod_id zcornsoy_yield zclay_pct rents_yes insurance_yes zage zcons_pmt_baseline|| group:

**Reduced model; non-significant parameters eliminated, but still controlling for vignette parameters

xtmelogit irrigate_yes_c prob_n cons_pmt_change_ plant_early_ plant_late_ rain_more_ rain_less_ harvest_late_ harvest_early zlog_cropland_acres zedu rents_yes zcons_pmt_baseline || group:

# 5. Supporting Information References

Arbuckle, J. G. (2013). Farmer support for extending Conservation Compliance beyond soil erosion: Evidence from Iowa. *Journal of Soil and Water Conservation*, *68*(2), 99–109. https://doi.org/10.2489/jswc.68.2.99

Burnett, E., Wilson, R. S., Heeren, A., & Martin, J. (2018). Farmer adoption of cover crops in the western Lake Erie basin. *Journal of Soil and Water Conservation*, *73*(2), 143-155.

Jose, P. E. (2013). *Doing statistical mediation and moderation*. Guilford Press.

Lavoie, A., & Wardropper, C. B. (2021). Engagement with conservation tillage shaped by “good farmer” identity. *Agriculture and Human Values*, *38*(4), 975-985.

McGuire, J. M., Morton, L. W., Arbuckle, J. G., & Cast, A. D. (2015). Farmer identities and responses to the social–biophysical environment. *Journal of Rural Studies*, *39*, 145–155. https://doi.org/10/gm8d6v

Schreiber, J. B. (2008). Core reporting practices in structural equation modeling. *Research in Social and Administrative Pharmacy*, *4*(2), 83-97.

Valizadeh, N., Bijani, M., Karimi, H., Naeimi, A., Hayati, D., & Azadi, H. (2020). The effects of farmers’ place attachment and identity on water conservation moral norms and intention. *Water Research*, *185*, 116131.

Wallander, S., Smith, D., Bowman, M., & Claassen, R. (2021). Cover crop trends, programs, and practices in the United States. U.S. Department of Agriculture, Economic Research Service. Retrieved 25^th^ April, 2024 from: <https://ageconsearch.umn.edu/record/309562/files/EIB-222-Cover-Crop-Trends-Programs-and-Practices-in-the-United-States.pdf>

West, S. G., Taylor, A. B., & Wu, W. (2012). Model fit and model selection in structural equation modeling. *Handbook of Structural Equation Modeling*, *1*, 209-231.
